# Modelling evolutionary pathways for commensalism and hypervirulence in *Neisseria meningitidis*

**DOI:** 10.1099/mgen.0.000662

**Published:** 2021-10-27

**Authors:** Christopher A. Mullally, August Mikucki, Michael J. Wise, Charlene M. Kahler

**Affiliations:** ^1^​ The Marshall Center for Infectious Diseases Research and Training, School of Biomedical Science, University of Western Australia, Perth, Australia; ^2^​ School of Physics, Mathematics and Computing, University of Western Australia, Perth, Australia; ^3^​ Telethon Kids Institute, Perth Children’s Hospital, Perth, Australia

**Keywords:** clonal complexes, commensalism, competition, pathogenicity

## Abstract

*
Neisseria meningitidis *, the meningococcus*,* resides exclusively in humans and causes invasive meningococcal disease (IMD). The population of *N. meningitidis* is structured into stable clonal complexes by limited horizontal recombination in this naturally transformable species. *N. meningitidis* is an opportunistic pathogen, with some clonal complexes, such as cc53, effectively acting as commensal colonizers, while other genetic lineages, such as cc11, are rarely colonizers but are over-represented in IMD and are termed hypervirulent. This study examined theoretical evolutionary pathways for pathogenic and commensal lineages by examining the prevalence of horizontally acquired genomic islands (GIs) and loss-of-function (LOF) mutations. Using a collection of 4850 genomes from the BIGSdb database, we identified 82 GIs in the pan-genome of 11 lineages (10 hypervirulent and one commensal lineage). A new computational tool, Phaser, was used to identify frameshift mutations, which were examined for statistically significant association with genetic lineage. Phaser identified a total of 144 frameshift loci of which 105 were shown to have a statistically significant non-random distribution in phase status. The 82 GIs, but not the LOF loci, were associated with genetic lineage and invasiveness using the disease carriage ratio metric. These observations have been integrated into a new model that infers the early events of the evolution of the human adapted meningococcus. These pathways are enriched for GIs that are involved in modulating attachment to the host, growth rate, iron uptake and toxin expression which are proposed to increase competition within the meningococcal population for the limited environmental niche of the human nasopharynx. We surmise that competition for the host mucosal surface with the nasopharyngeal microbiome has led to the selection of isolates with traits that enable access to cell types (non-phagocytic and phagocytic) in the submucosal tissues leading to an increased risk for IMD.

## Data Summary

The computer program, Phaser, can be accessed via this DOI: 10.26182/4xm3-2390. We confirm all supporting data, code and protocols have been provided within the article or through supplementary data files.

**Impact Statement**

*
Neisseria meningitidis* is a Gram-negative diplococcus that resides exclusively in humans and is the causative agent of invasive meningococcal disease (IMD). The population of *N. meningitidis* is structured into stable clonal complexes by limited horizontal recombination in this naturally transformable species. *N. meningitidis* is an opportunistic pathogen, with some clonal complexes, such as cc53, effectively acting as commensal colonizers, while other genetic lineages, such as cc11, are rarely colonizers but are over-represented in IMD and are termed hypervirulent. To understand how these lineages evolved, we examined the abundance and association of genomic islands (GIs) from genetic lineages representing commensal colonizers and hypervirulent lineages. This study identified a sub-set of GIs from the accessory meningococcal pan-genome which are present in variable and unique combinations associated with genetic lineages of meningococci. Hypervirulent lineages were enriched for GIs encoding functions associated with meningococcal survival in phagocytic cells. In contrast, commensal colonizing lineages such as cc53 have not acquired GIs associated with survival in phagocytic cells but have accumulated 13 unique loss-of-function loci and seven unique GIs suggesting a pathway of adaptive evolution for the trait of commensalism. This work provides a framework in which to examine the adaptation of a single species towards both commensalism and pathogenicity.

## Introduction

*Neisseria meningitidis* is a Gram-negative diplococcus that resides exclusively in humans and is the causative agent of invasive meningococcal disease (IMD). IMD manifests as two clinical syndromes, systemic bacteraemia resulting in septic shock and meningitis, which may occur separately or together [1]. The meningococcus is acquired by respiratory droplet transmission and leads to localized inflammation, resulting in signalling to attract resident macrophages and dendritic cells [1, 2]. Colonization rates are highest in the 19–25 year old age group and prevalence varies between 5 and 30 % at any given time, dependent upon geographical location and socio-economic conditions such as crowding and smoking [3, 4]. Amongst those who are colonized, persistence depends upon bacterial load and strain, with either continuous colonization or strain replacement occurring over periods of a few weeks to 8 months in any given host [5]. Colonization of the host rarely results in IMD, suggesting that host factors are involved. These include inhibition of meningococcal growth by the nasopharyngeal microbiome [6] and an inability to reach the mucosal cells which are protected by airway mucus [7]. Previous exposure to similar strains induces natural immunity that is protective against IMD [8], while certain immunocompromised conditions of the host, such as defects in the complement pathway and inflammatory cascades, may result in recurrent IMD [9].

Multi-locus sequence typing and core genome phylogenies have shown that *N. meningitidis* forms a structured population of stable clonal complexes (cc) which have been conserved by limited horizontal recombination in this naturally transformable species [10]. Metadata analysis of large population-scale meningococcal carriage studies have shown that meningococcal clonal complexes are not equally associated with IMD [11]. Clonal complexes with a disease to carriage (D/C) ratio greater than 0.5 are considered hyperinvasive, while the least invasive lineage – cc53 (which has a D/C ratio of <0.1) – is considered truly avirulent. It was inferred from these natural history studies that there is a diversity in the pathogenicity of the clonal complexes, and Stollenwerk *et al*. [12] used this as the basis to develop a mathematical model which showed that the variation in meningococcal pathogenesis drives the appearance of meningococcal disease outbreaks.

A broad series of studies have identified many virulence determinants associated with hypervirulent meningococcal lineages [13–17]. However, only two determinants have been found exclusively in the hypervirulent lineages. These are the possession of a capsule polysaccharide synthesis (*cps*) cluster [11] and multiple copies of the meningococcal-disease-associated phage (MDA ϕ) [18]. Advanced studies examining overall population structure and gene diversity driven by the combined forces of recombination and natural transformation, respectively, have inferred that meningococcal virulence is polygenic, being dependent upon not only the presence of the known virulence determinants but also upon other factors such as metabolism and mechanisms of in-host adaptation [19]. Schoen *et al*. [20] presented preliminary evidence that the metabolism of strains from each clonal complex were biologically distinct, in particular noting that both the glutathione metabolism and de-nitrification pathways are linked to pathogenesis.

In this study, we examined the association of genomic islands (GIs) with hypervirulent and non-virulent meningococcal lineages and assessed the correlation of GI presence/absence with D/C ratio in human disease. We used a novel tool, Phaser, to identify genes that were inactivated by simple sequence repeats (SSRs) or indels to identify loss-of-function (LOF) loci associated with the commensal and hypervirulent lineages. Using cc53 as the non-virulent comparator in these datasets, we have been able to infer potential evolutionary pathways for commensalism and invasiveness in this species.

## Methods

### Identification of the core and accessory genomes

In total, 550 whole-genome sequences (WGSs) of *N. meningitidis* from 11 clonal complexes were selected from the BIGSdb database to analyse the core genome [21] (Table S1, available in the online version of this paper). These 11 clonal complexes were selected if they had at least 100 submitted genomes representing a variety of geographical locations over a 5 year period.

Core and accessory genomes were defined as outlined in Fig. S1. Fifty genome sequences were randomly chosen from each clonal complex and compared using the BIGSdb Genome comparator tool within the PubMLST website (http://pubmlst.org/neisseria/) [22], which automatically annotates known loci in *N. meningitidis* and uses these loci for comparison to identify identical, conserved and divergent loci (Table S1). All loci were analysed for putative function using blast with a minimum blast identity of 70 % and a blast word size of 20. The core genome was defined as loci that were present in at least 90 % of isolates, while the accessory genome was defined as loci that were found in at least one isolate but present in fewer than 90 % of all isolates (Fig. S2). Alignments of each locus in the core genome for all 550 isolates were produced using Genome Comparator. These alignments were imported into Geneious v7 and a neighbour-joining tree was created using the Tamura–Nei genetic distance model with 100 bootstraps and a support threshold of 50 % [23] (Fig. S3A). Using the same method, a neighbour-joining (NJ) tree was generated based on alignments of all genes in the accessory genome (Fig. S3B).

### Identification of genomic islands within clonal complexes

The pipeline for determining prevalence of genomic islands in genetic lineages is outlined in Fig. S1. A total of 4850 whole genome sequenced strains were analysed for the presence of loci that were unique to any specific clonal complex. Four common hypervirulent clonal complexes were used: cc11 (*n*=1459), cc41/44 (*n*=833), cc32 (261) and cc269 (*n*=601). Six clonal complexes less often associated with invasive disease were also used: cc213 (*n*=275), cc22 (*n*=141), cc23 (*n*=720), cc461 (*n*=101), cc5 (*n*=142) and cc60 (*n*=103). The genetic lineage cc53 was used to represent an avirulent meningococcal lineage (*n*=52). The prevalence of untagged loci was identified by running nucleotide blast against all 4850 isolates with a blastn word size of 20. Using a distribution analysis (Fig. S2), genes that were detected in >80 % of isolates within a clonal complex were considered present. This cut-off was chosen as the database being searched contained unclosed genomes and hence by chance alone a gene may have been absent from a record due to incomplete loci that are located on the end of a contig. A total of 331 genes found at 63 positions around the genome were found to be associated with at least one clonal complex, suggesting that these loci were GIs.

The loci associated with GIs were numbered from 1 to 63 with the first insertion site located closest to the origin of replication and each locus then numbered sequentially around the genome (Table S2). Where an alternate island existed at the same locus, each alternate was labelled alphabetically. A circular representation of the neisserial genome was generated using the BRIG image generator in order to show the position of each GI within the genome [24].

The GIs were analysed for the presence of tRNAs, tandem repeats and G+C content. The online tool tRNAscan-SE was used to identify tRNAs for each GI using the default parameters for bacterial genomes [25]. The online tool ‘Tandem repeats finder’ was used to identify tandem repeats flanking the GIs using the default search parameters [26]. Finally, the G+C percentage of each coding sequence was determined using Artemis v17 [27].

The genes in the pan-accessory genome (i.e. the combined accessory genome of all 11 clonal complexes) were directly compared between cc11 (*n*=1459) and cc53 (*n*=52) using the presence/absence tool in PubMLST. If genes were found to be missing this was confirmed using a blastn on the sequence with a minimum sequence identity of 70 %. The presence of genes that were not found in cc53 was then detected in the remaining nine lineages: cc41/44 (*n*=833), cc32 (261), cc269 (*n*=601), cc213 (*n*=275), cc22 (*n*=141), cc23 (*n*=720), cc461 (*n*=101), cc5 (*n*=142) and cc60 (*n*=103). A phylogenetic tree based on the relative presence of the different GIs was computed using the Bayesian phylogenetic tree reconstruction program MrBayes [28]. For each clonal complex, the percentage presence of each GI was expressed as a decile. In other words, rather than a sequence of amino acid or nucleotide letters corresponding to a clonal complex, the input was a string of digits, which were assumed to be ordered, with each digit representing the decile presence of the corresponding gene. A uniform clock was assumed, with a gamma distribution used to model rate variation between sites and a proportion of invariant sites. A chain length of 5 000 000 was used. The default all-compatible method was used to compute the consensus tree. A node with posterior probability ≥0.8 is considered to be highly significant.

### Calculation of disease carriage ratio

This ratio was calculated using a dataset of 4183 carriage isolates and 4862 disease isolates from the EUMenNet Study and the UK Carriage study between 1999 and 2002 [29, 30] (Table 1). The D/C ratio was calculated as the proportion of strains within a clonal complex found from disease cases divided by the proportion of strains within a clonal complex found from carriers. A D/C ratio for cc5 was not calculated as insufficient data were available for this lineage.

**Table 1.** Disease/carriage ratio of clonal complexes used in this study

Clonal complex	No. of carriage isolates	No. of disease isolates	Disease/carriage ratio
cc11	81	903	12.59
cc213	806	74	0.37
cc22	1109	67	0.21
cc23	351	68	0.07
cc269	367	256	2.29
cc32	169	707	2.01
cc41/44	1163	1014	0.96
cc461	44	43	1.35
cc60	413	65	0.23

### Identification of non-random phase variation between virulent and non-virulent clonal complexes

The pipeline for the Phaser analysis is shown in Fig. S1. Phaser is a currently unpublished software tool that detects frameshifts in ORFs and applies a *t*-test with a Bonferroni-corrected *p*-value (<0.05) to detect genes with statistically significant distributions of translational frame status between pairwise datasets. A total of 562 isolates representing the 10 most common pathogenic clonal complexes and cc53 were selected from the PubMLST. A pairwise comparison of 50 genomes from each lineage was independently performed with 48 genomes of cc53 (Table S3) to identify genes that had a different translational frame status from the avirulent lineage. The Phaser output generates a list of genes by NEIS code and Uniprot code, and extracts the nucleotide sequence in which the frameshift occurs. This enables the categorization of the mechanism of translational frameshift into two broad categories: those translational frameshifts associated with SSRs which are considered reversible and those caused by transition/inversions resulting in a premature stop codon or an indel that irreversibly results in an loss-of-function (LOF).

## Results

### Distinct sub-sets of the pan-accessory genome of *N. meningitidis* are associated with different clonal complexes

**Fig. 1.**
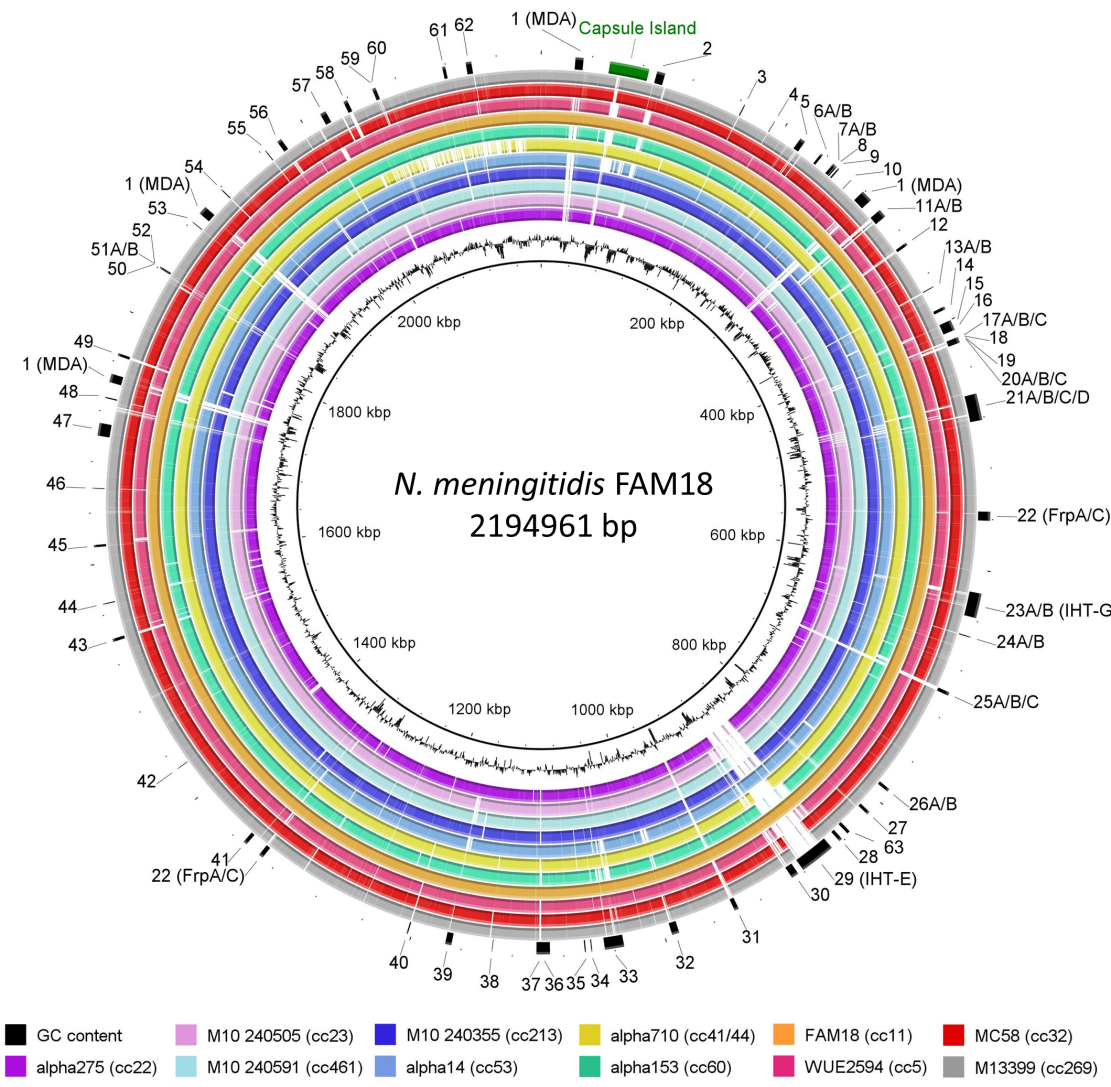
Location of 82 genetic islands (GIs) in 11 representative genomes from different clonal complexes. GIs that were associated with at least one clonal complex are marked numerically on the outer ring from 1 to 63. Where there are multiple islands at one locus the islands are labelled alphabetically as islands A, B, C and D. These flexible loci bring the total to 82 GIs. The closed genome FAM18 was used as the reference genome and is shown as the innermost ring. The capsule island is labelled in green and was not assigned an island number. The image was generated using BRIG [24]. The innermost black trace shows G+C content.

The pan-genome of *N. meningitidis* was analysed in 550 isolates and the frequency at which loci were present was determined for 2562 annotated loci (Fig. S2). Based on this histogram, the cut-off for a locus to be present in any lineage was set at 80 % due to the use of unclosed genomes. An NJ tree based on the 1735 core genes from 550 isolates revealed 11 distinct clusters consistent with the PubMLST scheme (Fig. S3A). The remaining 827 genes were deemed to comprise the accessory genome (Fig. S3B). To examine if accessory genes were associated with clonal complexes, the prevalence of all accessory genes across a subset of 4850 isolates from PubMLST was determined. Genes that were detected in >80 % of isolates within a clonal complex were considered characteristic for the lineage (Fig. S2). Using this method, 496 accessory genes (60%) were sporadically dispersed across all genetic lineages. The remaining 331 accessory genes formed 82 GIs located at 63 distinct loci (Fig. 1, Table S2) and these had a G+C content ranging from 18.1 to 63 %. The G+C content of 66 GIs (80.5%) was significantly different (greater than three standard deviations) from the mean G+C content of the meningococcal genome (Table S2). Using BLASTp, 43 % of genes had high sequence similarity to genes from within the family *Neisseriaceae,* 9 % had high sequence similarity (>70 % identity) to genes found in commensal flora of the nasopharynx, and the remaining 48 % showed similarity to genes from bacteria that are not found in the human nasopharynx and do not belong to the family *Neisseriaceae *.

Using the predicted functionality of the encoded proteins in the GIs (Table S3), the GIs were categorized into eight functional groups: phage, toxin, iron acquisition, membrane proteins, restriction modification, cell wall synthesis/cell division, metabolism and hypothetical proteins (Fig. S4). Cc53 possessed the least number of GIs (*n*=29/81) while cc11 possessed the largest number of GIs (*n*=48/81) (Fig. S4). Thirteen loci had more than one variant of a GI in the same position, and these were termed flexible GI (fGI) loci (Fig. 1, Table 2). In seven loci (6, 7, 8, 15, 27, 31, 37), the alternate GIs appeared to encode different putative functions while the remainder encoded similar putative functions. Of these, three GIs – 8, 15A and 15B – have been functionally verified. GI 8 encodes the adenosine monophosphate-protein transferase NmFic required for regulating cell division by adenylating the GyrB subunit of DNA gyrase [31]. GI 15A encodes a meningococcal fratricidal protein, meningocin, originally described by Allunans *et al*. [32]. 15B encodes a CRISPR-Cas locus which regulates the sensitivity of the bacterial host to phage infection [33, 34].

**Table 2.** fGIs in *N. meningitidis
*

Locus	Variants	Reference
6	A=SMI1/KNR4 family involved in cell division B=single hypothetical CDS	[71]
7	A=type II restriction endonuclease B=zinc-dependent protease	
8	A=adenosine monophosphate-protein transferase NmFic B=hypothetical permease	[31]
15	A=bacteriocin island (BGI-1) B=CRISPR Cas	[93]
25	A=short patch repair endonuclease (Vsr-1) B=putative restriction/modification C=putative restriction/modification	[51]
27	A=a single CDS hypothetical B=four CDS hypothetical	
31	A=WYL-domain protein B=putative DNA helicase C=FunZ protein	[94]
32	A=7 kb P2-like phage B=33 kb P2-like phage	
33 (IHT-E)	A=PNM-2 B=Mu-like MenB phage	[48, 95, 96]
37	A=five CDS B=putative type 1 restriction modification system	
49	A=unknown integral membrane protein B=yfcA-related integral membrane protein	

CDS, coding sequence.

**Fig. 2.**
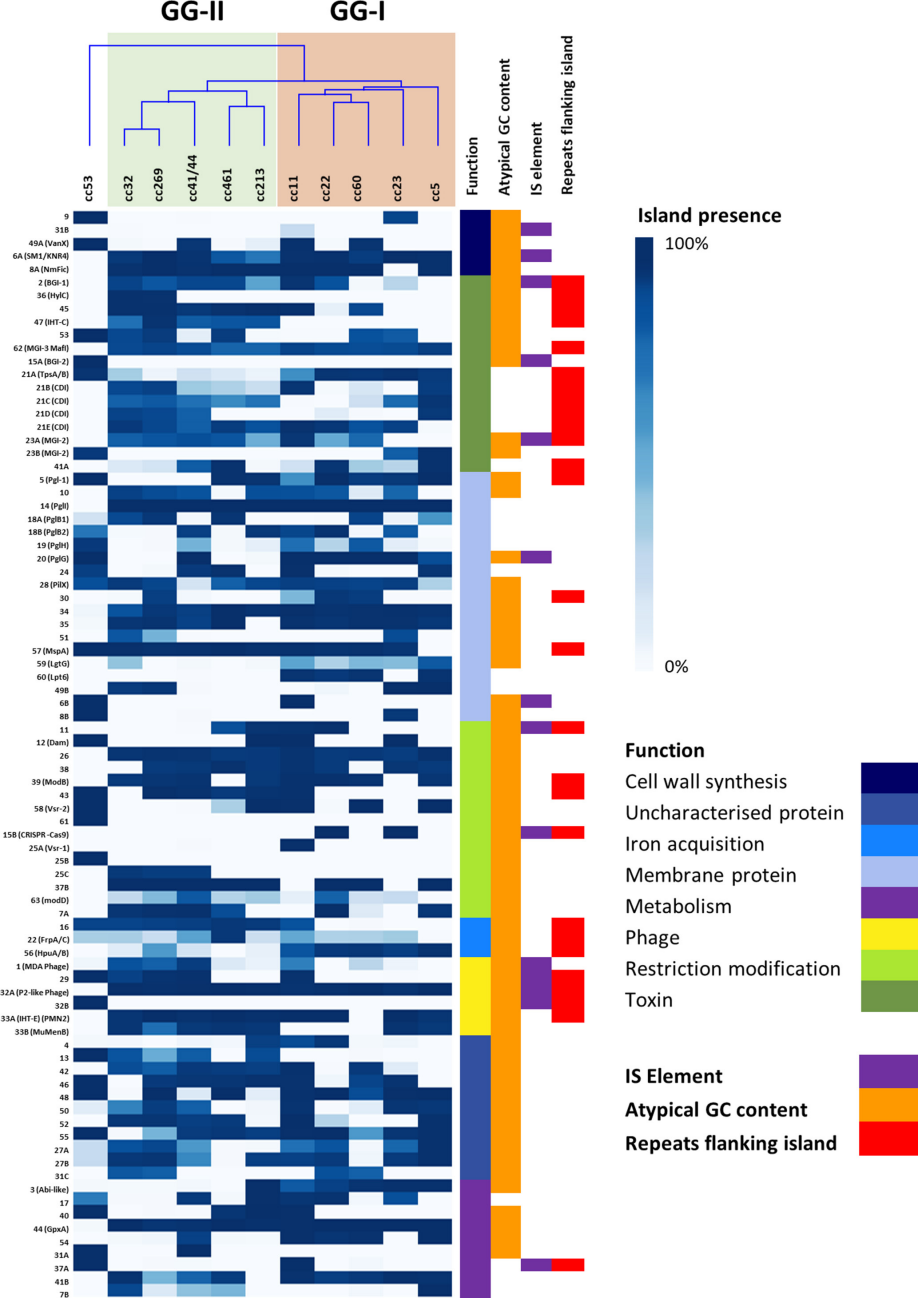
Prevalence of 82 genomic islands within 11 meningococcal clonal complexes. The prevalence of each island within each lineage is shown in blue. The function of each island is shown in a different colour. The presence of IS elements, repeats flanking the island and atypical G+C content is also shown. If a cell is white, the island or feature is absent. The GIs were grouped according to functionality and ranked within each functional group by prevalence. The clonal complexes were grouped based on hierarchical clustering of the presence and absence of the GIs, and a corresponding dendrogram was generated. GG-I is shown in orange and GG-II is shown in green. Outgroup cc53 can be seen on the left hand side of the tree.

Considering that many GIs were found in multiple clonal complexes, GIs were also assessed for elements such as integrases, flanking inverted repeats and IS elements, which may confer mobility to these islands (Fig. 2). Only GIs 25 (Restriction modification island), 27 (Restriction modification island) and 59 (Membrane protein) had an organization consistent with a minimal mobile element (MME) as defined by Saunders and Snyder [35], i.e. a region of DNA that is flanked by two conserved genes with different genomic content found between strains, with no mechanism of horizontal transfer other than homologous recombination.

Thirty-eight per cent (*n*=31/82) of GIs were associated with IS elements and/or direct repeat (DR) flanking sequences. fGIs 6A/B, 15A and 31B were associated with an IS element without a DR while 15B and 32A/B had both IS elements and direct repeats flanking the islands (Table 1). A further two GIs – GIs 1 and 20 – were associated with an IS element without a DR. These GIs encoded the MDA-ϕ and PglG, an accessory glycosyltransferase in the pilin glycosylation operon, respectively [36, 37].

Nineteen GIs had repeat sequences flanking them, but no IS element associated with them. This set of GIs contained 10 GIs of known function. GI 21 encodes the contact-dependent inhibition (CDI) system, TpsAB, inserted downstream of NEIS0441. GI 21 has a mosaic structure, and five variants were identified in this dataset [38]. GI 21A encoded only TpsAB and did not possess the repeating arrays of *tpsC* elements required for antigenic variation of the toxin domain. The remaining variants contained *tpsC* arrays of varying length and composition. A second locus, GI 62, corresponds to the MGI-3 inserted near the *pyrH* locus but in this instance is a modified variant in which the MafI immunity gene is absent [39]. The remaining GIs were: MspA (GI 57), an autotransporter involved in adhesion to epithelial cells [40]; ModB (GI 39), a type III R/M system involved in global regulatory responses via methylation [41]; HpuA/B (GI 56), the haptoglobin utilization system required for scavenging iron from host proteins [42]; and FrpD/C (GI 22), which encodes an RTX-toxin [43, 44]. The remaining nine GIs encoded CDS with a variety of putative functions. GI 5 contained the CDSs encoding proteins from the major facilitator superfamily [45, 46]. GI 30 and GI 36 encoded a putative haemolysin family D protein with an accompanying ABC transporter inserted near NEIS0422 and a haemolysin-activating lysine acyltransferase (HlyC) inserted near NEIS1108, respectively. GI 41A and GI 45 encode a putative addiction module protein and a two-component toxin–antitoxin system of unknown function [47]. GI 47 and GI 33A encode the IHT-C locus initially described by Tettelin *et al*. [48] and PNM2 bacteriophage [49], respectively. The last two GIs from this group encode an R/M system (GI 43) and a homologue of an esterase (GI 16), both of unproven function.

Five GIs (2, 11, 23A, 29, 37A) had both IS elements and direct repeats flanking the islands. These five GIs had atypical G+C content with the exception of GI 37A. Three GIs encoded known virulence determinants. GI 2 encodes a putative bacteriocin-encoding genomic island (BGI-2). GI 23A encodes a second copy of the MGI-3 Maf-based CDI system described by Jamet *et al*. [39] inserted at a different locus, *ntpA* (NEIS0585). GIs 29 and 32B had an organization consistent with that of pathogenicity-associated islands (PAIs), as they were flanked by direct repeats, possessed an integrase and were associated with a tRNA (Fig. S5, Table S2). GI 29 (IHT-E) has previously been identified as a P2-like phage [50]. GI 32B has not been previously identified and appears to possess all the genes necessary for formation of full phage particles (Table S2). GI 11 encodes a known R/M system previously described by Claus *et al*. [51]. The remaining GI, 37A, has an unknown function and encodes six CDSs encoding a β-lactamase fold protein, an MarR family transcriptional regulator, a prokaryotic cytochrome b561 family protein, a NADH dehydrogenase, a monooxygenase and a hypothetical protein.

### A subset of hypervirulence-associated genes are associated with meningococcal invasiveness

**Fig. 3.**
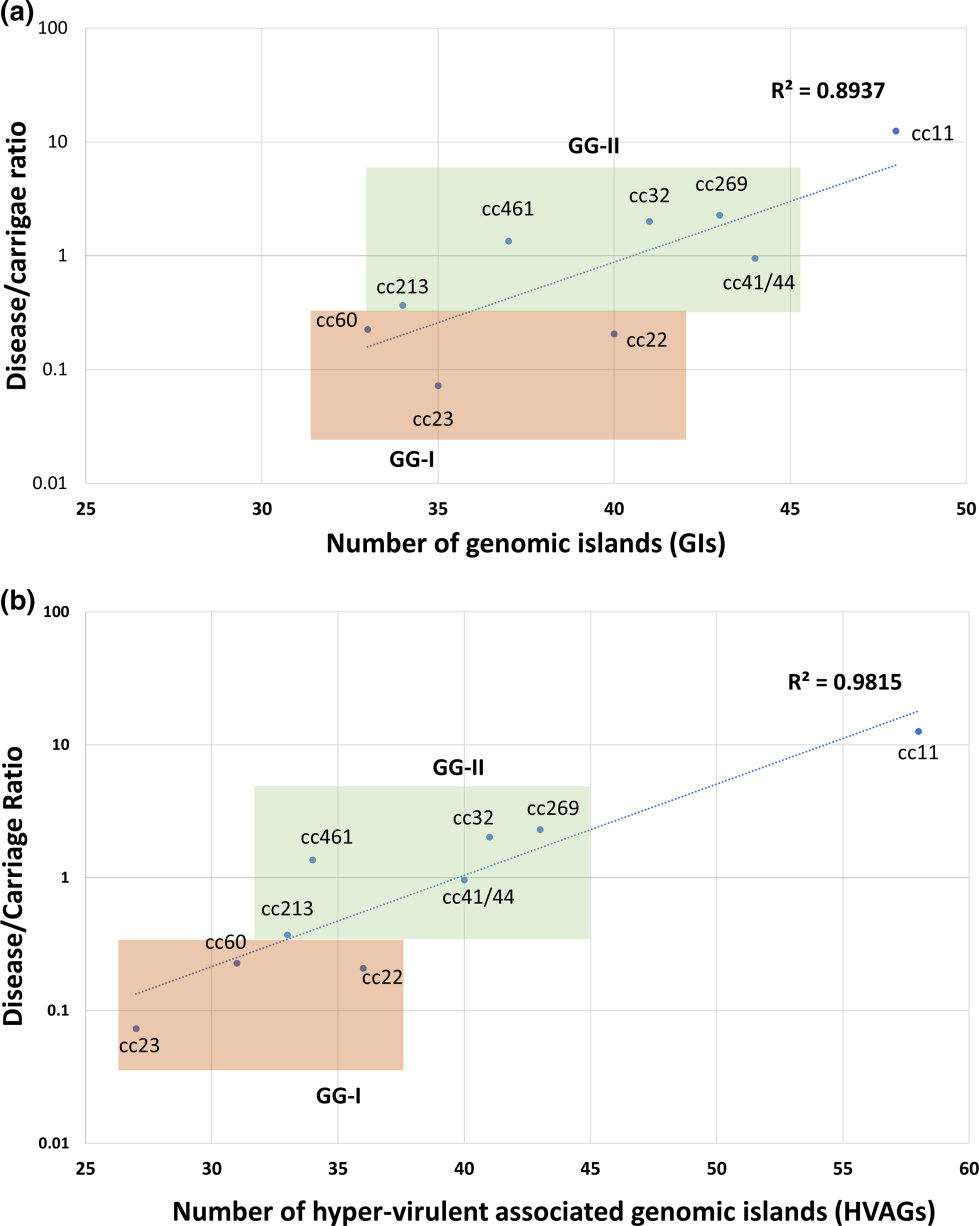
Disease/carriage ratio of each pathogenic clonal complex plotted against all 82 GIs of the accessory genome (a) and hypervirulence-associated genes (HVAGs) consisting of 35 GIs and 26 genes (b). The disease/carriage ratio was an *in silico* calculation (see Methods and Table 1). GG-I is shaded in orange while GG-II is shaded in green.

Unsupervised heircharical clustering of the prevalence of GIs within the clonal complexes revealed that they formed into two genogroups, I (GG-I) and II (GG-II). GG-I consisted of cc11, cc22, cc23 and cc60; and GG-II consisted of cc32, cc269, cc461, cc213 and cc41/44 (Fig. 2). To further investigate whether the formation of GG-I and GG-II was associated with the biology of the pathogen, a linear regression analysis was performed using the D/C ratio as a measurement of invasiveness in humans (Table 1). When the number of pan-accessory genes was plotted against D/C ratio (Fig. 3a), the possession of different subsets of GIs in each clonal complex indicated a moderate effect size *R* ^2^ of 0.8937. However, the clustering of GG-I and GG-II remained intact across the linear regression. It was notable that cc11, which had the highest calculated D/C ratio, did not co-occur with other members of GG-I (Fig. 2). To investigate this further, the list of GIs associated with cc11 was re-examined using a pair-wise comparison with cc53 (Fig. S6). While both clonal complexes shared an accessory genome of 583 genes, cc53 possessed 65 unique genes and cc11 possessed 144 unique genes (Table S4). This subset of 144 genes in cc11 were termed hypervirulence-associated genes (HVAGs) which are not present in cc53 but are variably present in all hypervirulent lineages. The HVAGs comprised 30 GIs that had already been identified, plus an extra 40 genes, 14 of which formed five novel islands: HI-1 to HI-5 (Table S4). A linear regression plot of these 40 genes and five GIs resulted in a stronger positive correlation with D/C ratio (*R* ^2^ of 0.9815) suggesting that the distribution of these HVAGs in each clonal complex was associated with invasive potential (Fig. 3B).

**Fig. 4.**
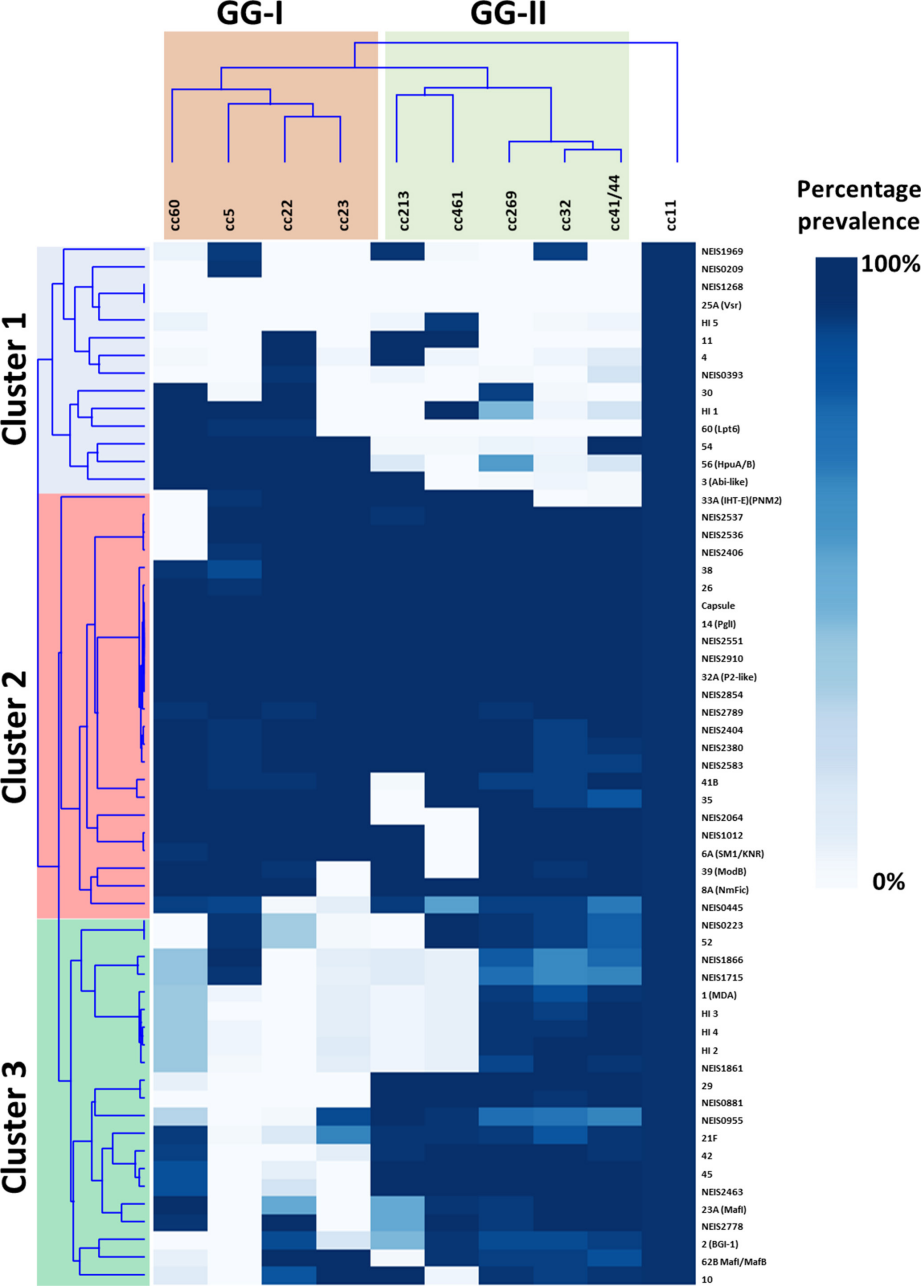
Two-way hierarchical clustering showing the association of hypervirulence-associated genes (HVAGs) with all hypervirulent lineages. Clonal complexes are hierarchically clustered based on the presence of the HVAGs and a corresponding two-way dendrogram was generated. Where a GI has been assigned a number, it has been listed next to the genomic island. Genomic islands comprising hypothetical genes have been denoted as HI 1 to 5.

To visualize the cohorts of GIs that are driving the association with D/C ratio, hierarchical clustering was performed on the prevalence of GIs across all genetic lineages (Fig. 4). Three clusters were observed: Cluster 1 are GIs that are present in cc11 and are to some extent enriched in GG-I but mostly absent in GG-II, Cluster 2 are those GIs common to all hypervirulent lineages and Cluster 3 are GIs common to GG-II and cc11.

**Fig. 5.**
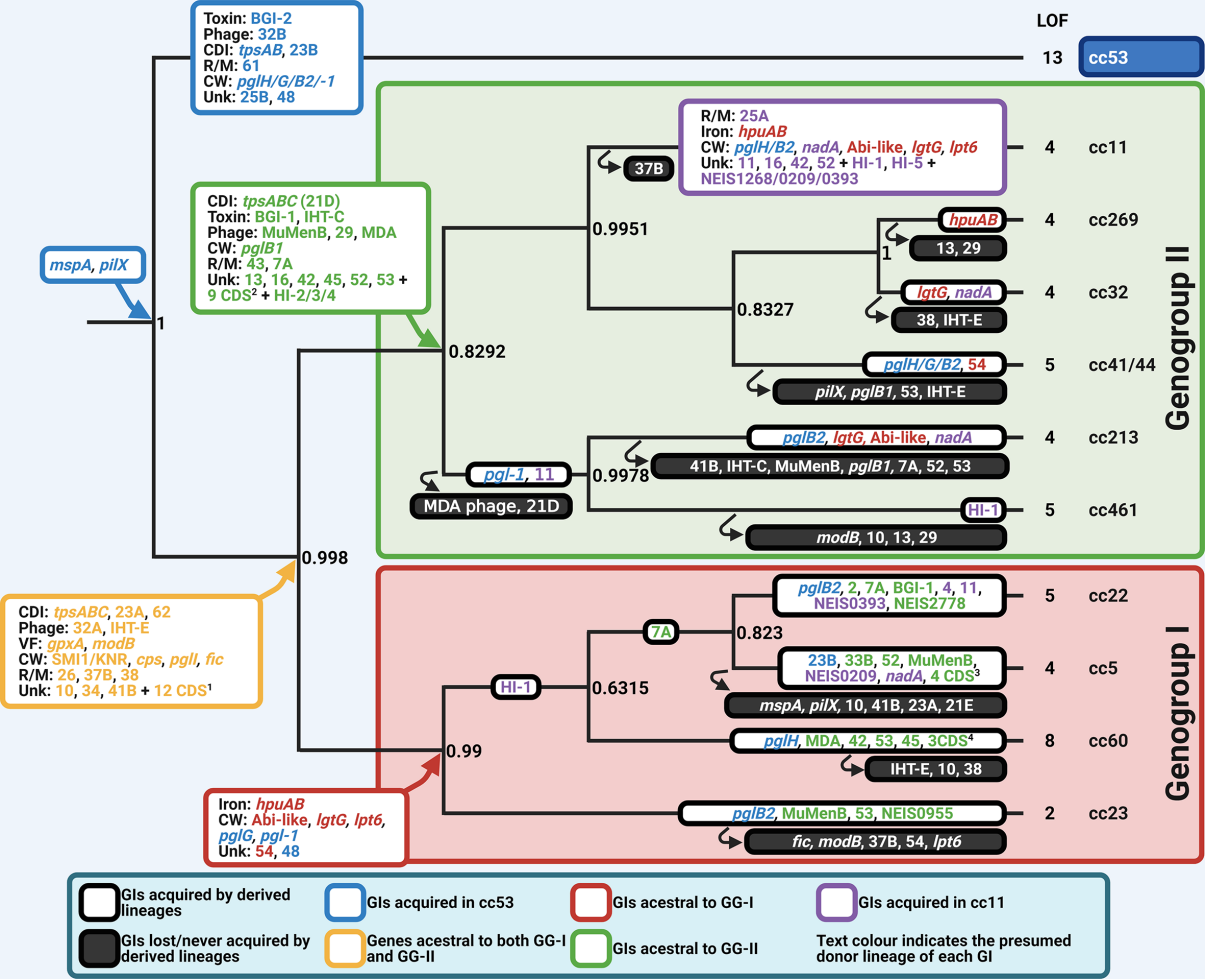
A decision tree model illustrating the putative relationships of modern clonal complexes with the acquisition and loss of genomic islands in ancestral *N. meningitidis *. The combined genomic island dataset from Figs 2 and 4 was input into MrBayes to draw a Bayesian tree (see Methods for parameters). Each node shows the Bayesian posterior probability (0 to 1.0) of the split into the different clusters of GIs that are associated with a clonal complex. A node with posterior probability ≥0.8 is considered to be highly significant. At each node, the GIs that contributed to the formation of each group are shown. The far left hand node is the putative ancestral split between the commensal cc53 (blue text) and the early specialists (yellow text) that evolved to become genogroups I (red text) and II (green text). The GIs that are characteristic of cc11 are indicated in purple text. GIs characteristic of the ancestral node are coloured according to lineage. Absence of GIs in any given lineage relative to the origin node is shown in a solid black box. This model cannot distinguish between whether these GIs were not acquired at the ancestral node by the founding progenitor of the lineage or that the GI was acquired and subsequently lost during the evolution of the clonal complex. Created using Biorender.com. ^1^ = NEIS1012/2064/2583/2380/2404/2854/2551/2910/2789/2406/2536/ 2537. ^2^ = NEIS2778/2463/0955/0881/1861/1866/1715/0223/0445. ^3^ = NEIS1866/1715/0223/0445. ^4^ = NEIS/2778/2463/0445.

To visualize the distribution of the GIs in the commensal and hypervirulent lineages, a presence decile tree was constructed based on the percentage presence or absence of all GIs in each lineage (Fig. 5). This analysis supported three nodes with high probabilities: the commensal cc53 lineage splitting from the hypervirulent lineages (node 0.99), and the formation of s GG-I and GG-II (0.99 and 0.82). The decile percentage tree was overlaid with the identity of the GIs that were present in >80 % of trees at each node. The only GIs common to cc53 and most hypervirulent lineages were MspA and PilX. PilX is least abundant in two hypervirulent lineages – cc41/44 and cc5 – while MspA is absent from cc5. cc53 possessed four unique GIs: GI 15A, encoding a novel bacteriocin synthesis pathway; GI 25B, encoding two hypothetical proteins; GI 32B, encoding a large bacteriophage; and GI 61, an unknown R/M system. These were not shared with any other lineage. cc53 lacked 48 GIs (<20 % prevalence) found in the hypervirulent lineages. However, it shared 30 GIs (>80 % prevalence) and was enriched for three GIs (>50 % prevalence) found in one or more hypervirulent lineage. Of these 33 GIs, only eight have a known function and were involved in pilin glycan synthesis and CDI. These were PglH (GI 19), PglG (GI 20) and PglB2 (GI 18B), all of which are co-located in the *pglFBCD* operon and an island Pgl-1 (GI 5)*,* consisting of three CDSs of unknown function next to *pglA.* cc53 possessed the fGI 21A-encoding TpsAB locus lacking the *tpsC* arrays and an MGI-3 *Maf-*encoding GI at a novel locus*, ntpA* (GI 23B)*.*

Of the 47 GIs not found in cc53, 17 were common to more than eight of 10 hypervirulent lineages and may suggest the formation of an ancestral population in meningococci from which the modern day hypervirulent lineages are descended. Three GIs encoded elements of contact-dependent inhibition systems (GI 23A, GI 21E and GI 62). Four cell wall modification GIs were found. GI 6A encodes a putative cell division scaffold-protein from the SMI1/KNR family and GI 8 encodes NmFic. The remaining two GIs are the capsule biosynthesis island and PglI (GI 14). Of the remaining factors, only two have established roles in virulence. These are GpxA (GI 44), the glutathione peroxidase, required for resistance to hydrogen peroxide stress, a condition encountered inside macrophages [52], and the global transcriptional regulator ModB (GI 39).

From this ancestral core population, the two genogroups diverged and are distinguished by distinct core cohorts of GIs. GG-I is characterized by nine GIs of which seven have putative or known functions. Two GIs are associated with O-linked glycan synthesis. Pgl-1 (GI 5) is an insertion of three CDSs near *pglA* encoding the galactosyltransferase for the O-linked glycan, and the other is *pglG* inserted into the *pglFBCD* operon. GG-I also acquired three GIs associated with lipid A biosynthesis: *lgtG*, a glycosyltransferase [53], and *lpt6*, an O-6 phosphoethanolamine transferase [54, 55], that both modify the inner core heptose residue of endotoxin, in addition to the insertion of the Abi-like CDS (GI 3) in the lipid A biosynthesis operon [56]. GG-I also acquired the haptoglobin utilization operon, HpuAB, from an unknown donor. By comparison, all lineages in GG-II acquired 15 GIs that are not found in GG-I. GG-II contained a variant of the TpsABC locus (GI 21D), the secreted meningocin [32], and the putative RTX-toxin-encoding IHT-C locus [48]. Amongst the remaining 12 GIs, only two have known involvement in virulence: PglB1 and the MDA-ϕ, which appear in three of the clonal complexes in GG-II (cc32, cc41/44 and cc269).

Mapping the HVAGs across the decile percentage tree revealed that most of these loci are associated with the GG-II ancestral node and are scattered across the GG-I genetic lineages. It is this cohort of genes which has driven the relationship of cc11 with GG-II.

### Loss-of-function in 24 loci is associated with cc53 but not hypervirulent lineages

During evolutionary pathways for adapting to a new host there are two forces: the acquisition of new traits which must be accompanied by mechanisms to integrate the new genes into a regulatory framework, but also to modify existing pathways to conserve energy and reconcile metabolic pathways to enable a return to fitness [57]. We hypothesized that if cc53 had diverged towards a commensal lifestyle earlier than hypervirulent lineages, it may possess a cohort of LOF loci that would be more likely to be intact in the hypervirulent lineages.

To address this hypothesis, we used the ‘Phaser’ computational tool to detect differences in the translational frame of genes between datasets. This tool detects a change in translational frame by multiple mechanisms including SSRs or point mutations/indels. SSRs typically result in reversible LOF, with the frequency of these events varying between loci and isolates. Thus, some SSRs may be associated with an intact state in some genetic lineages but not others. In contrast, point mutations that introduce stop codons or indels in the absence of an SSR were considered to be irreversible LOF mutations.

**Fig. 6.**
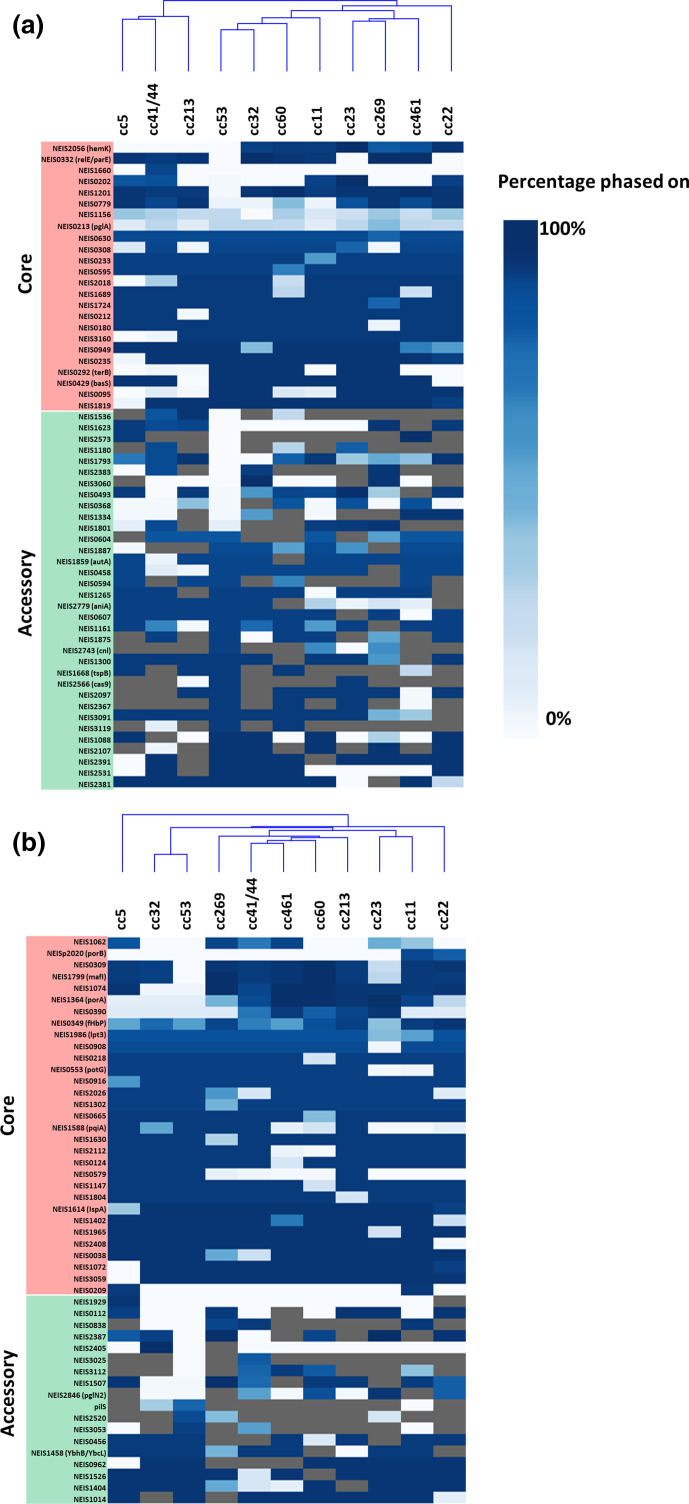
Heatmap of phase state of LOF loci by SSR (a) and indels (b). Clonal complexes were hierarchically clustered based on similarity of the phase state of each LOF loci and a corresponding dendrogram was generated. If a gene is always intact, it is shown in dark blue. If it is always interrupted, it is shown in white. If the gene is absent in the clonal complex, it is shaded grey. The phase state for a gene was considered significantly different between each clonal complex and cc53 if the corrected *p*-value was <0.05.

Phaser detected a total of 144 LOF loci, 105 of which were shown to have a statistically significant non-random distribution in phase status (Table S5). Of the 42 genes that were not statistically significant, 25 were shifted due to SSRs and 17 were due to indels. The 25 genes that were shifted by SSRs contained several known phase-variable genes including *pglA, pglI, pglG, pglB2* and *hpuA*. Of the remainder, 17 genes were shifted due to indels, including known phase-variable genes such as *lot, fhbp* and *lgtA*. Of the 105 non-random LOF loci, 47 were shifted due to indels and 58 were shifted due to SSRs (Fig. 6), and were evenly distributed between the core and accessory genome (52 and 53 respectively) (Table S5).

A pairwise comparison of cc53 with cc11 revealed LOF in 13 core genes and 21 accessory genes in cc53 that were present as an intact ORFs at <10 % frequency in the hypervirulent lineages. The 13 core loci were inactivated by indels (*n*=7/13) and SSRs (*n*=6/13). The genes inactivated by indels were NEIS1062, NEIS0309, NEIS1799 (*mafI-*MGI-1), NEIS1074, *porA* and NEIS0390. The genes inactivated by SSRs were NEIS2056 (*hemK*), NEIS0332 (*relE/parE*), NEIS1660, NEIS0202, NEIS1201 and NEIS0779. Apart from *porA* (NEIS1364), a known non-functional orthologue of the gonococcal locus, none of the loci have known functions. NEIS0202 was identified as a hypermutable locus during persistent nasopharyngeal carriage [58], which may support the hypothesis for its involvement in colonization. Of the remaining genes with hypothetical functions, NEIS0332, encoding a type II toxin-antitoxin, could be involved in initiating cell division while NEIS2056 may encode *hemK*, a peptide methyltransferase that controls translation termination [59]. Of the 21 accessory loci, 11 were inactivated by indels and 10 by SSRs. Only two named alleles were identified in this group: NEIS2846, which encodes PglN2, a variant glycosyltransferase for modifying the proteoglycome, and NEIS1180, encoding a restriction enzyme NalVM.

To examine if the LOF loci in cc53 were active in other hypervirulent lineages, a pair-wise comparison in Phaser revealed that the 13 core genes were present in all hypervirulent lineages and were intact at a frequency above 80 % in at least two or more of any of the hypervirulent lineages (Fig. 5). In terms of the accessory LOF loci, when present in the accessory genomes of a hypervirulent lineage, any single locus could be found intact at a frequency above 80 % in any other hypervirulent lineage. Unsupervised hierarchical clustering analysis of the number of LOF loci that were intact in each clonal complex did not result in the formation of GG-I and GG-II (Fig. 6). A linear regression analysis with D/C ratio did not detect any association with invasiveness (data not shown).

## Discussion

The genus *Neisseria* contains many species of environmental, mammalian and reptilian origin [60], and it has been hypothesized that a transition to humans occurred in the distant past. Priniski and Seifert [61] presented a model in which they hypothesized that an early ancestor evolved into all 11 of the obligate human colonizing *Neisseria* spcies. Vazquez *et al*. [62] had advanced an earlier model implying that *N. meningitidis* and *N. gonorrhoeae* share a common ancestor and recent work from Vigue and Eyre-Walker [63] substantiated this further, showing evidence that both species have diverged through adaptive evolution propelled by lateral gene transfer. In the current study, we extended this theory to examine the role of GIs associated with commensalism and pathogenesis in *N. meningitidis *. This study used the power of large genome libraries (in this case 4850 genomes) to identify GIs in a much larger pan-genome and to calculate lineage-associated prevalence in both commensal and hypervirulent lineages. Relationships of co-occurrence of GIs within each genetic lineage provided the rationale for interpreting traits associated with niche adaptation, virulence and competition in different genetic lineages and putative common ancestral populations.

**Fig. 7.**
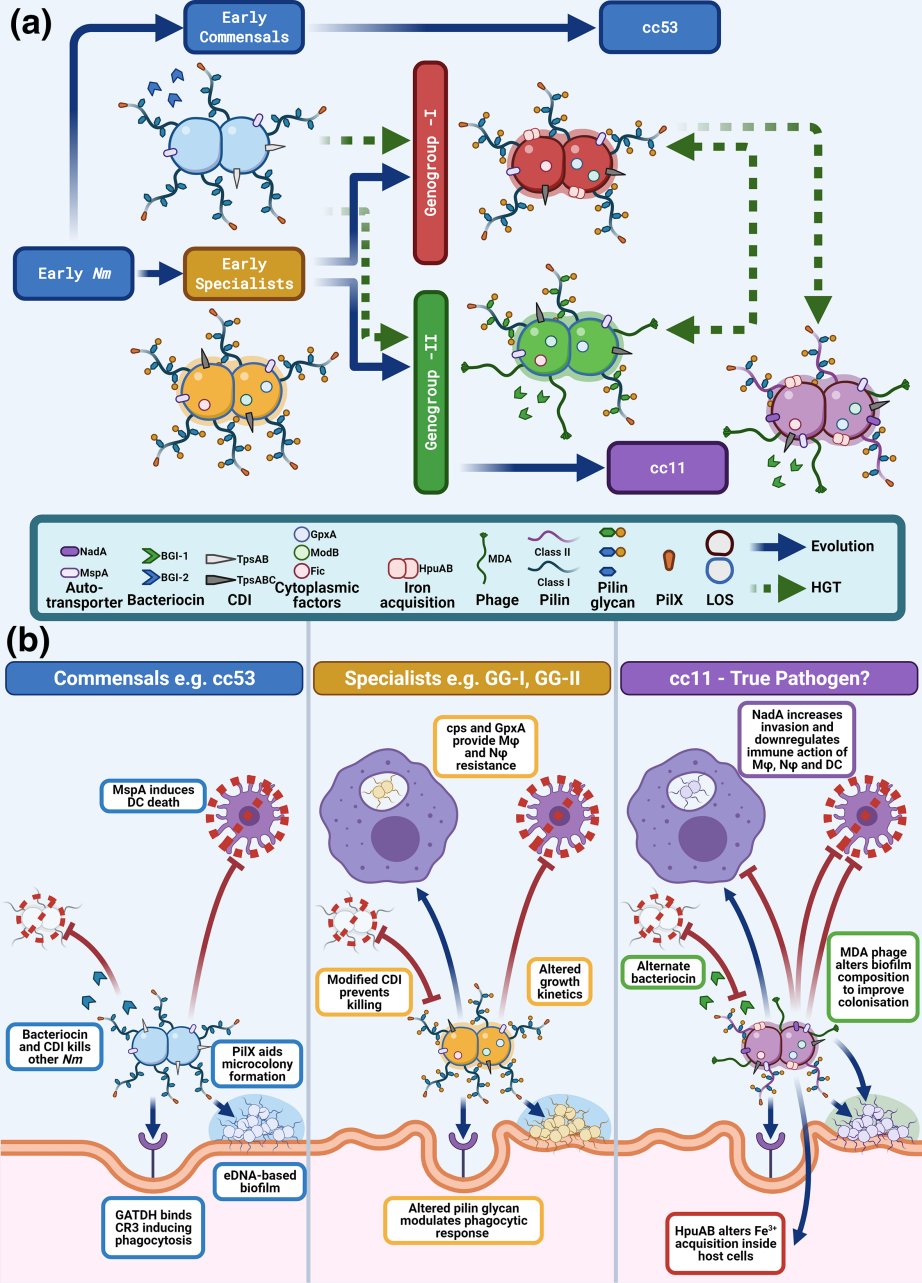
Putative biological functions of ancestral meningococci: commensals, early specialists and modern IMD lineage cc11. (a) Proposed stages in the evolution of the meningococcus. The early meningococcus, the commensal lineage (cc53, blue), an early specialist population (yellow node), GG-I (red), GG-II (green) and cc-11 (mauve). These colour codes correspond to the nodes in Fig. 5. Known virulence determinants are represented in the bottom panel by icons which appear in relationship to the cartoon representation of the meningococcus. Pilin glycan is represented by two different colour codes corresponding to: Hex_(2-4)_-GATDH O-linked glycan (blue) and O-acetyl-Hex _(1-2)_ diNAcBac glycan (green). A yellow circle represents O-acetylation of the glycan. Type IV pili are shown as two classes: class I (blue) and II (purple). (b) Proposed interactions of cc53, early specialists and cc11 with host cells derived from the known functions of GIs enriched in each population (Fig. 5). Abbreviations: DC = dendritic cells, Mφ = macrophages, Nφ = neutrophils. Figure created using Biorender.com.

Assuming that a presence prevalence of a GI >80 % in a given lineage is associated with an ancestral founder with improved fitness, the presence decile tree of GIs in *N. meningitidis* (Fig. 5) can be loosely interpreted as a model of the ancestral evolutionary pathway of adaptation to the human host. Hung and Christodoulides [64] have recently summarized an extensive list of adhesins that are important for meningococcal adherence to human cells. Modern cc53 and all of the hypervirulent lineages possess the same adhesins PorB, TspA and NnhA [64] in their core genomes. In contrast, autotransporter MspA and minor pilin PilX were placed in the accessory genome due their low prevalence in cc5 and cc41/44 (Figs. 2 and 5). PilX is a minor pilin required for aggregation of the pili, improving micro-colony formation on epithelial cells in the early stages of nasopharyngeal colonization [65]. MspA is a type Va secreted autotransporter that binds to mannose receptor and transferrin receptor 1 of dendritic cells initiating apoptotic cell death, thus supressing the immune response [66]. Because of the conservation of PilX and MspA across all lineages except for cc5 and cc41/44, we have proposed that these GIs entered the early ancestral population from which both the commensal and the hypervirulent lineages were derived (Figs. 5 and 7).

A comparison of all GIs present in hypervirulent lineages with cc53 led to the observation that these populations obtained distinct sets of GIs (Figs. 2 and 5). The commensal population, represented by cc53, is characterized by the possession of GIs that are known to improve colonization and are predicted to enhance competition against the evolving microbiome of the host. Of the factors with known attributes, the pilin glycosylation system of commensal cc53 is composed of *pglFGH(B_2_)CD* [67]. PglB2 is necessary for the synthesis of glyceroamido-acetamido trideoxyhexose (GATDH). PglH and PglG add glucose or *N*-acetyl-glucosamine residues to undecaprenyl-GATDH to create O-linked di-, tri- or tetra-saccharide subunits that decorate pilin and the proteoglycome [68]. Based upon the *pgl* loci, the predicted proteoglycome of cc53 is Hex_(2-4)_-GATDH O-linked glycan. When attached to pilin, the glycan binds the CR3-receptor to initiate endocytosis into host cells [69] and functions as an immune evasion mechanism to mask dominant protein epitopes [70]. cc53 has also acquired two factors that support improved competition with the nasopharyngeal microbiome: a putative secreted bacteriocin (BGI-2) and the TpsAB system for contact-dependent inhibition of other meningococcal strains [38]. TpsAB form a two-partner secretion system that exports TpsA to the bacterial surface where it has multiple roles in adherence, biofilm formation and contact-dependent inhibition of fraternal cells by interacting with the BamA outer membrane protein in lipopolysaccharide transport. A second contact-dependent inhibition system, multiple adhesion family (Maf) (GI 23B), was also present which encodes MafB, a secreted EndoU ribonuclease which acts as a toxin in non-immune bacteria [39]. cc53 also possessed 13 unique LOF loci, which have been inactivated relative to the IMD isolates. As these loci have no proven functions, their relevance is currently unknown, but presumably these loci are likely to modify fitness through changes in metabolism. Together, the attributes of the GIs suggest further niche adaptation to the human nasopharynx by improving the ability of the commensal lineages to compete against other microflora.

In contrast to the cc53 lineage, all hypervirulent lineages evolved from a common ancestral node that was characterized by 17 GIs and 12 CDSs (Fig. 5, yellow node). This putative common ancestor may represent an early specialist population (Fig. 7). The early specialists possess a putative SMI1/KNR protein and NmFic which regulate cell division processes. SMI1/KNR proteins have been shown to co-ordinate cell wall synthesis [71] while NmFic is an adenylyltransferase controlling DNA gyrase activity and hence chromosome replication [31]. These factors could be involved in changing growth rate, and indeed it has been observed by Schoen *et al*. [20] that hypervirulent lineages grow faster than commensal lineages. ModB, a global epigenetic regulator, is also present in this group and absent from cc53, suggesting global regulatory changes in gene expression have occurred [72]. The early specialists have acquired known virulence factors that enable evasion of phagocytic cell killing, including capsule biosynthesis islands and the glutathione peroxidase [73]. This population also had acquired PglI, which further modifies the pilin glycan with an O-acetyl group that is known to affect the chain length of the glycan [70]. Lastly, the TpsAB system evolved with the appearance of the repeat cassettes containing *tpsC* tracts and immunity ORFs (IORFs). Functional studies by Arenas *et al*. [38] suggest that the numbers of repeat cassettes are related to the dose dependency of the IORFs, which may improve immunity against fraternal isolates expressing antigenically variable TpsA toxins. The Maf system has also changed with a second variant, GI 23B, replacing the original island in the same locus. It is thought that switching the highly polymorphic N-terminal regions of MafB is a mechanism to escape cross-immunity in a bacterial population [39], again hinting at increased competition against cc53.

From this common pool of ancestral GIs, two branches have elaborated, termed GG-I and GG-II, which encompass the modern hypervirulent lineages (Fig. 5). GG-I is enriched for the presence of an Abi-like protein predicted to be involved in cell division, the haptoglobin iron uptake system HpuAB, a modified endotoxin inner core via LgtG and Lpt6, and retains the O-acetyl-Hex_(2-4)_-GATDH O-linked glycan characteristic of the ancestral node of the putative early specialists. Haptoglobin synthesis is induced by endotoxin and macrophage-produced inflammatory cytokines such as IL-1, IL-6 and tumour necrosis factor alpha (TNF-α) [74]. This suggests that GG-I may have evolved to unlock a necessary iron source in the human host to enable rapid growth. In contrast, GG-II is enriched for traits that are involved in adherence and competition with the microbiome. GG-II has switched from a GATDH-linked glycan to the *N,N*′-diacetylbacillosamine (diNAcBac) [75], creating an O-acetyl-Hex _(1-2)_ diNAcBac glycan, which may function as an immune evasion mechanism [76] against cross-reacting antibodies raised against the commensal lineage and GG-I. It has acquired a secreted meningocin fratricidal bacteriocin (BGI-1) [32] and the MDA-ϕ, both of which could improve colonization by killing competing microflora and forming DNAase-resistant biofilms [77, 78]. Consistent with the observations by Wanford *et al*. [79, 80], there was no apparent relationship between the LOF loci, either derived by SSRs or indels, with GG-I and GG-II, reinforcing their conclusion that these events are more recent characteristics of modern clonal complexes. However, there was considerable exchange and absence/presence of the GIs amongst the modern lineages (Fig. 5). As an example, cc5 is the only lineage in which both PilX and MspA are absent, but it possesses a second autotransporter, NadA, and a phage MuMenB, a combination that is unique to this lineage. Thus, it is unclear if the absence of GIs in any given modern lineage is a result of the progenitor of this lineage never having gained the GI, or that it has been gained and subsequently lost through selective pressure or the presence of an alternative GI with similar functionality.

Meningococcal invasiveness is regarded as an evolutionary dead-end as this outcome from colonization in the human host does not promote further transmission. In our study, GG-I had a calculated D/C range of 0.07–0.2 while GG-II had a calculated range of 0.37–12. This is reasonably concordant with the original observations by Caugant and Maiden [11]. The most invasive clonal complex in both studies is cc11. Biological studies have confirmed that this lineage results in higher rates of fatal outcome in human infections, higher virulence in mice and greater damage to epithelial cells than other lineages [81]. Deghmane *et al*. [82] also demonstrated that cc11 induced a greater level of apoptosis and induced higher levels of TNF-α from macrophages than exemplar isolates from cc5 and cc32, suggesting a potent immunosuppressive phenotype. Although cc11 initially appeared associated with GG-1 (Fig. 2), a secondary analysis of the pan-genome of cc11 compared to cc53 revealed a further subset of GIs which firmly placed cc11 within GG-II (Fig. 5). This secondary analysis revealed that cc11 had received a third wave of GIs, most of which are hypothetical except for the autotransporter NadA. NadA increases invasiveness into host epithelial cells and has a strong immunomodulatory role on the responses of macrophages and dendritic cells to infection [83]. NadA exerts its affects by binding αβ1 integrins of M-cells, dendritic cells, macrophages and neutrophils of the submucosa to initiate invasion into these cell types. However, it is unclear if NadA is the only hypervirulence-associated factor as there were two other hypothetical GIs and four other ORFs that demonstrated similar associations with cc11. Lastly, cc11 is enriched for the possession of a class II Type IV pili (~70 % of all isolates in PubMLST, data not shown) [84]. While class I and class II pili variants are both involved in attachment to host cells [85], Class II pili lack the necessary motifs required for pilin antigenic variation, a mechanism used by class I pili to evade the antibody response [86]. Instead multisite glycosylation of class II pili is used as the immune evasion strategy by these isolates [76].

In summary, this study has identified a subset GIs of the accessory meningococcal pan-genome that are present in variable and unique combinations associated with genetic lineages of meningococci. Presumptively, these early events provide the selective pressure to acquire traits mediating meningococcal survival in these cell types [87]. The majority of the GIs identified had an origin within *Neisseriaceae* (principally *Kingella, Microvirgula* and *Simonsiella *, which are human colonizers), suggesting that the traits are shared amongst these species by natural transformation. Although very little is known about how commensal cc53 lineages suppress the host response to enable persistent carriage, it is known that their endotoxin has a lower inflammatory potential than virulent isolates [88], thus reducing macrophage and dendritic cell signalling and relieving the selective pressure to acquire traits related to evasion of these innate immune responses. Not only has the cc53 lineage not acquired GIs associated with survival in phagocytic cells such as the capsule island, but it has accumulated 13 unique LOF loci and seven unique GIs, suggesting a pathway of adaptive evolution for the trait of commensalism. The functional categories of the GIs also hint at mechanisms involved in competition between lineages such as the CDIs and secreted bacteriocins. Recent work has focused on the interactions of the commensal *Neisseria* species and their antagonistic relationship with pathogenic meningococci [84, 89], but it remains unknown if the factors possessed by cc53 would provide it with any advantage against these species.

This work provides a framework in which to examine the adaptation of a single species towards both commensalism and pathogenicity. The strength of study lies in the use of a large public database of unclosed genomes, which has enabled the creation of a presence decile tree to examine the relatedness of clustered GIs. We also used a novel computational search engine, Phaser, which examined translated ORFs to identify LOF loci by any mechanism. The model presented here has many limitations, including the observation that known virulence loci that have multiple copies (pilin, opacity proteins, phages to name a few) could not be included in this analysis as they are not assembled accurately or frequently in unclosed genomes [86, 90]. This model did not attempt to examine changes in function attributed to allelic variation, which is known to affect endotoxin structure [67, 91] and anaerobic respiration [92]. This model cannot provide any concept of the order in which traits were acquired or lost from individual lineages and will require further analysis using the techniques established by Vigue and Eyre-Walker [63] preferentially using a large library of closed genomes.

## Supplementary Data

Supplementary material 1
