# Supplementary material for: Modelling evolutionary pathways for commensalism and hypervirulence in Neisseria meningitidis
Source: Microb Genom. 2021 Oct 27;7(10):000662. doi: 10.1099/mgen.0.000662 (PMC8627216; doi:10.1099/mgen.0.000662)
Supplement: Supplementary material 1 [file mgen-7-0662-s001.pdf]

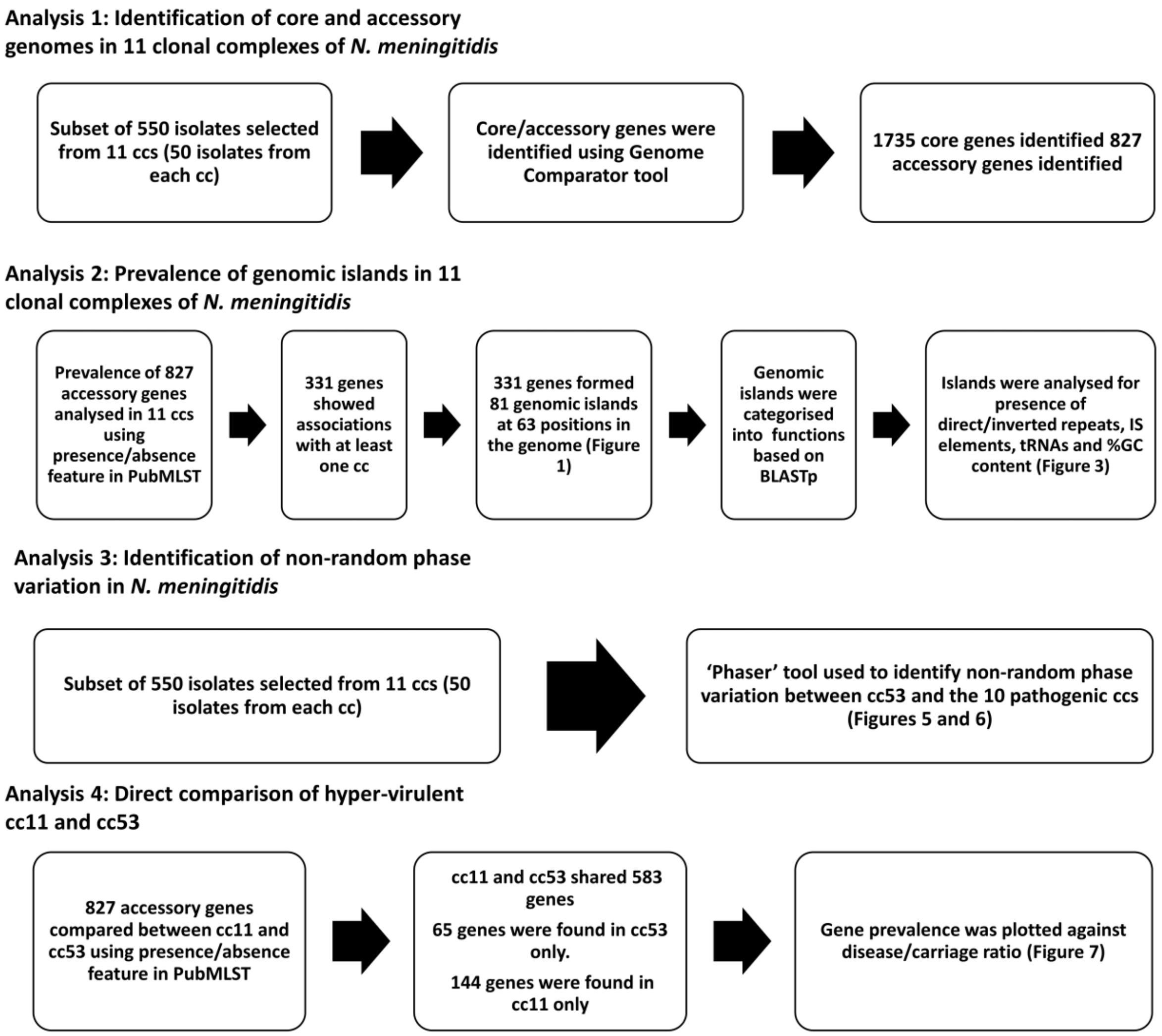

Supplementary Figure 1. Summary of methodology used in this study.

The five different analysis pipelines used in this study are summarised including the species, number of isolates, and clonal complexes analysed.

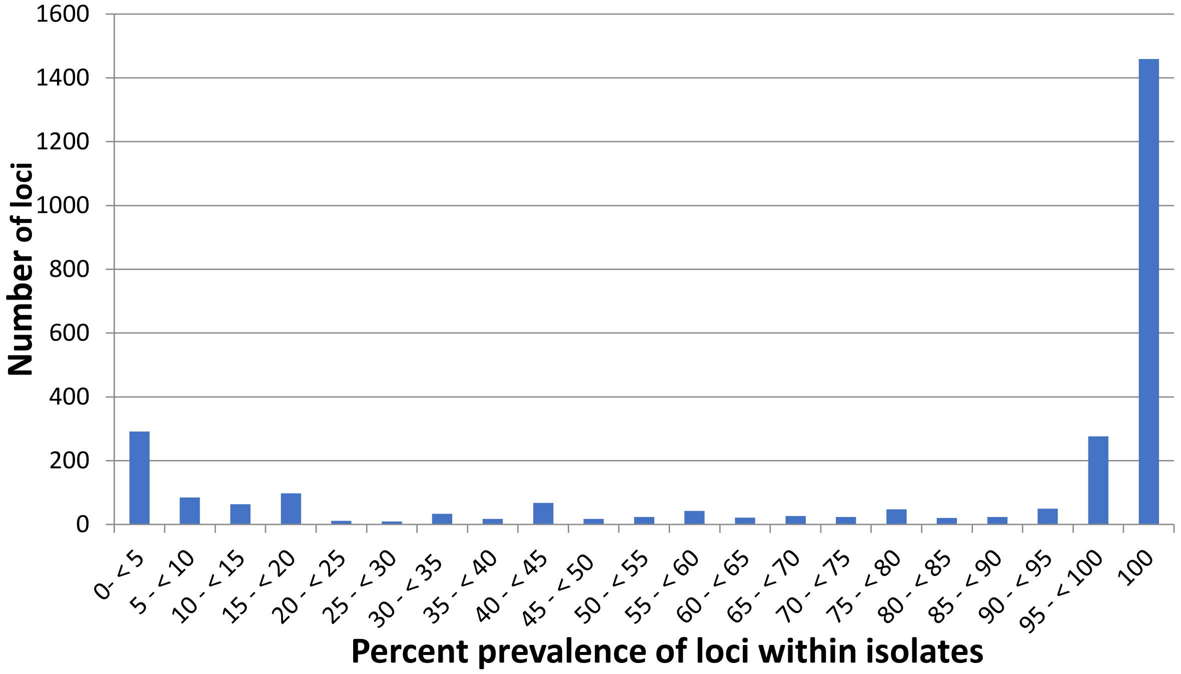

Supplementary Figure 2.

Locus presence frequency of pangenome of *N. meningitdis* consisting of 2562 genes in 550 whole genome sequences.

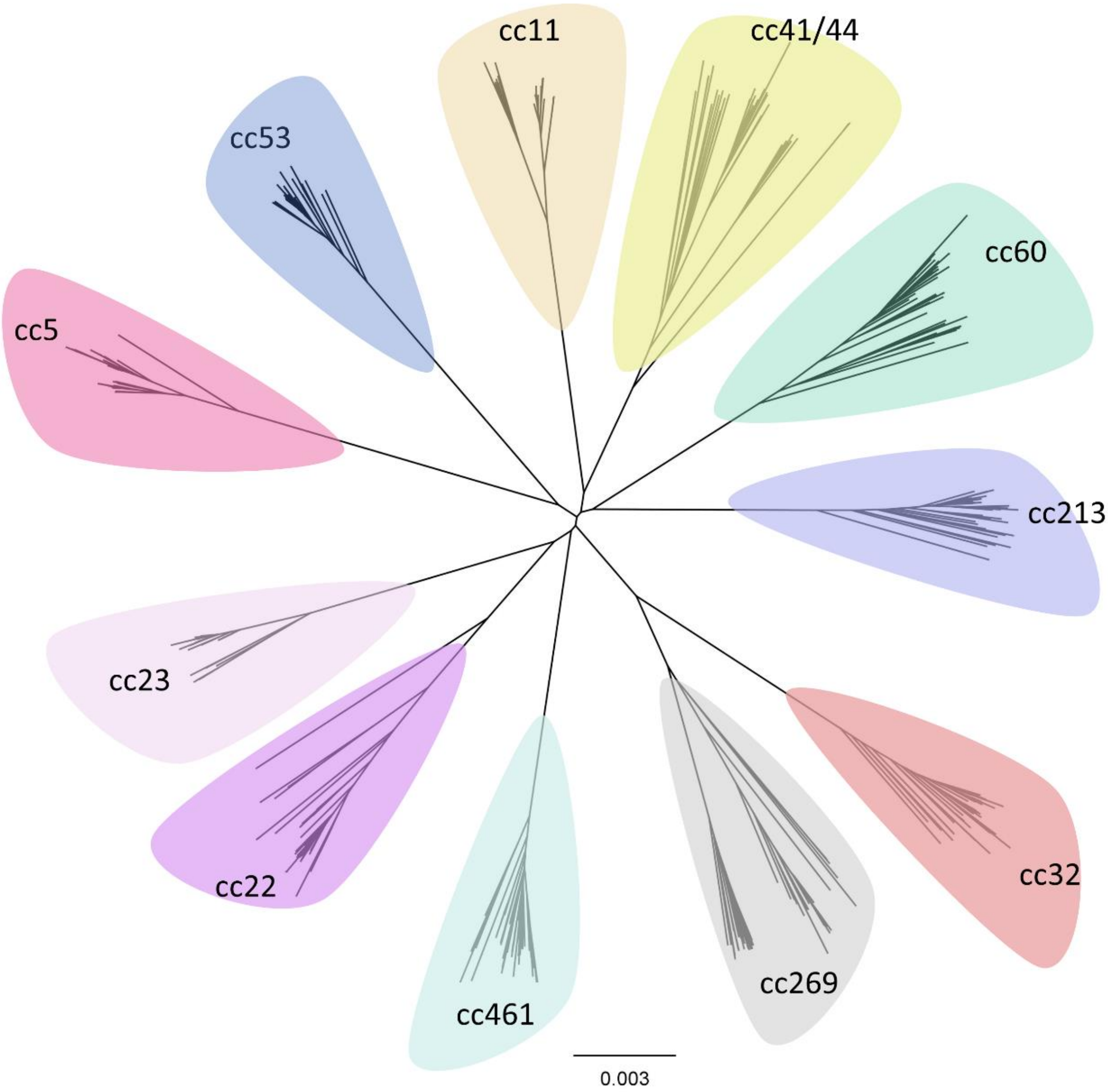

Supplementary Figure 3A. Evolutionary tree of *N. meningitidis* based on concatenated sequences from the 1735 genes that make up that core genome. The tree was generated from 550 isolates from 11 clonal complexes using the Neighbour-Joining method with the Tamura-Nei genetic distance model. Each of the 11 clonal complexes is shown in a different colour.

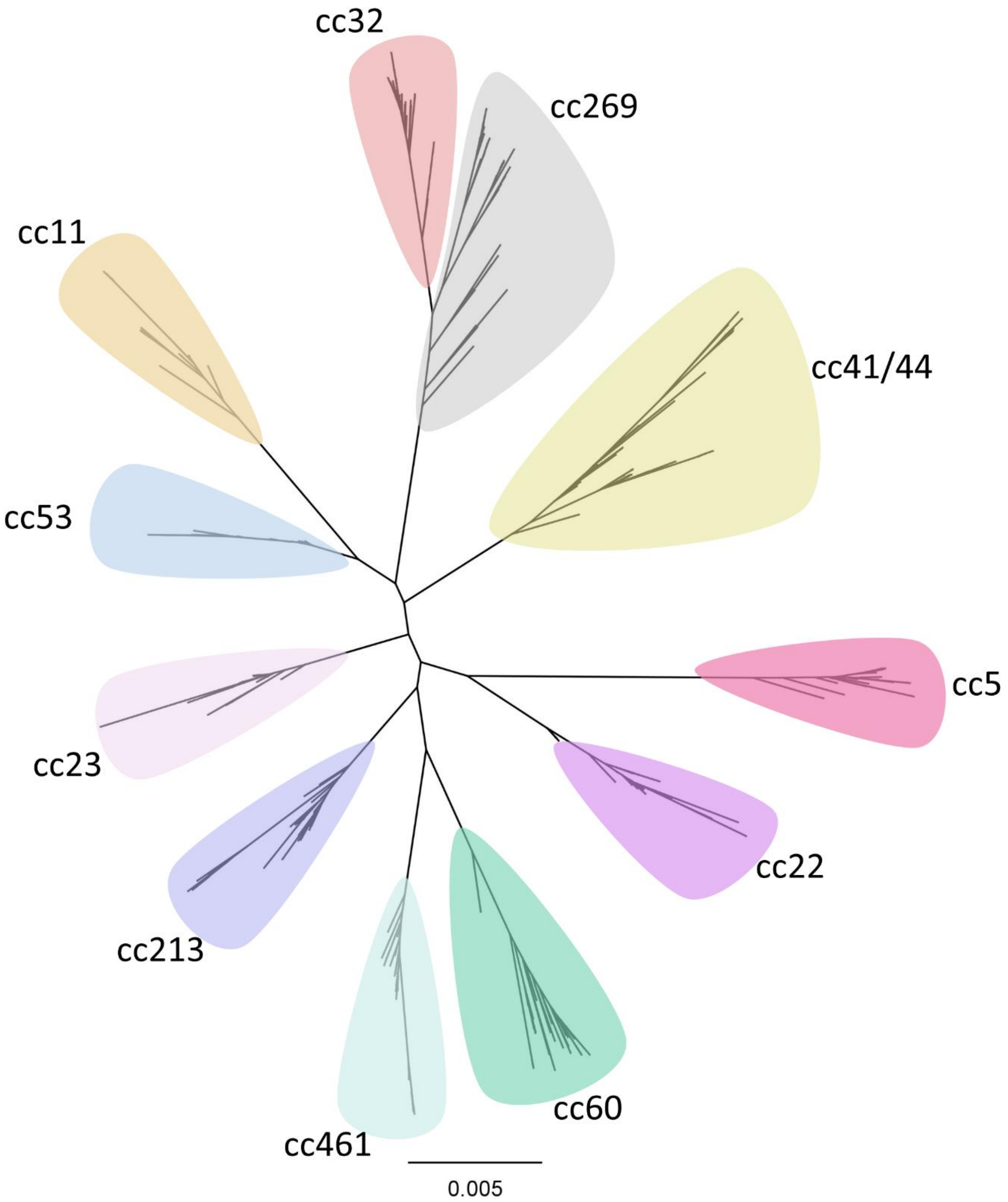

Supplementary Figure 3B. Evolutionary tree of *N. meningitidis* based on concatenated sequences from the 827 genes that make up the pan-accessory genome.

The tree was generated from 550 isolates from 11 clonal complexes using the Neighbor-Joining method with the Tamura-Nei genetic distance model. Each of the 11 clonal complexes is shown in a different colour.

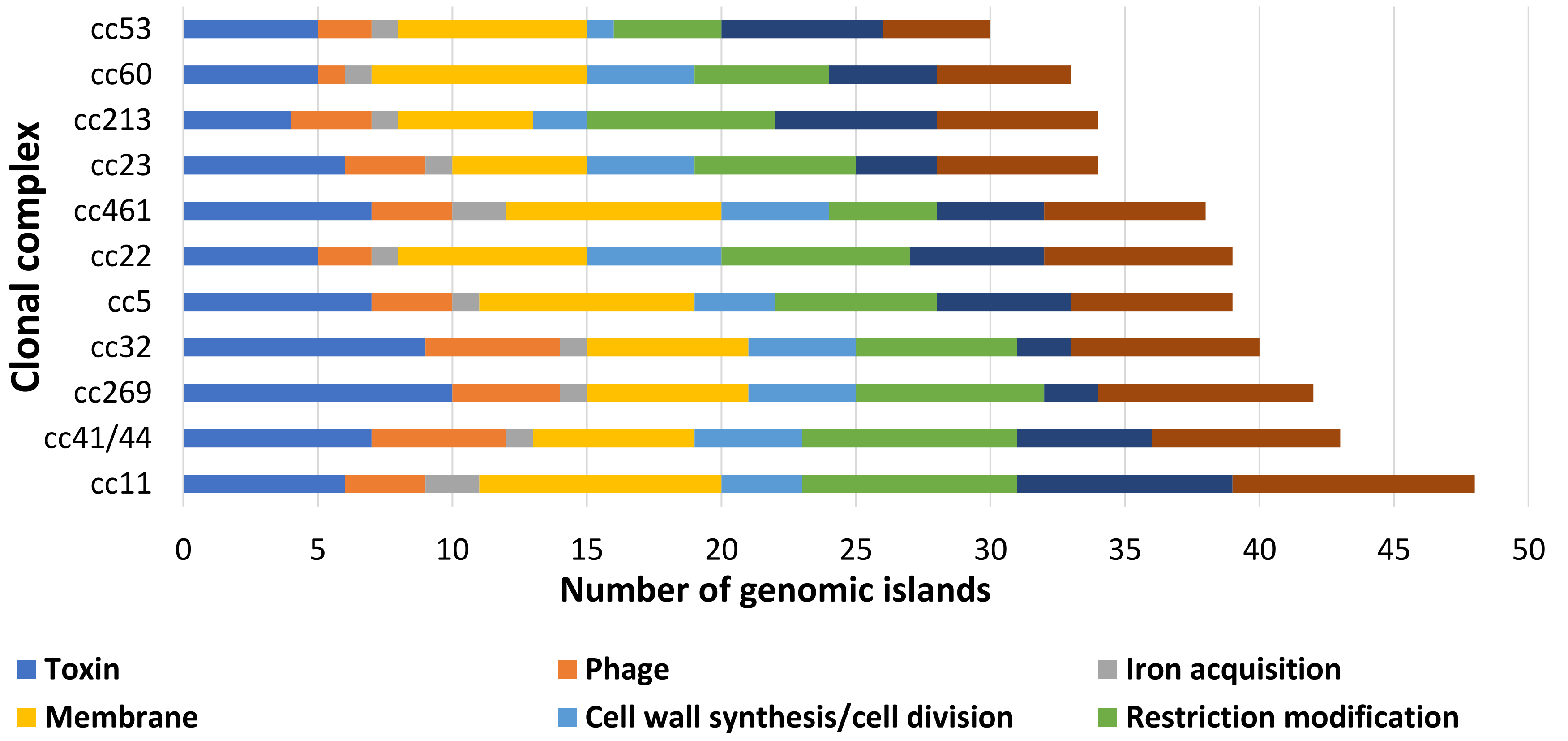

Supplementary Figure 4. Frequency of the 81 genomic islands amongst the 11 clonal complexes, by function.

Each functional category has been assigned a separate colour. If a GI was present in greater than 80% of isolates in a cc, it was counted as present.

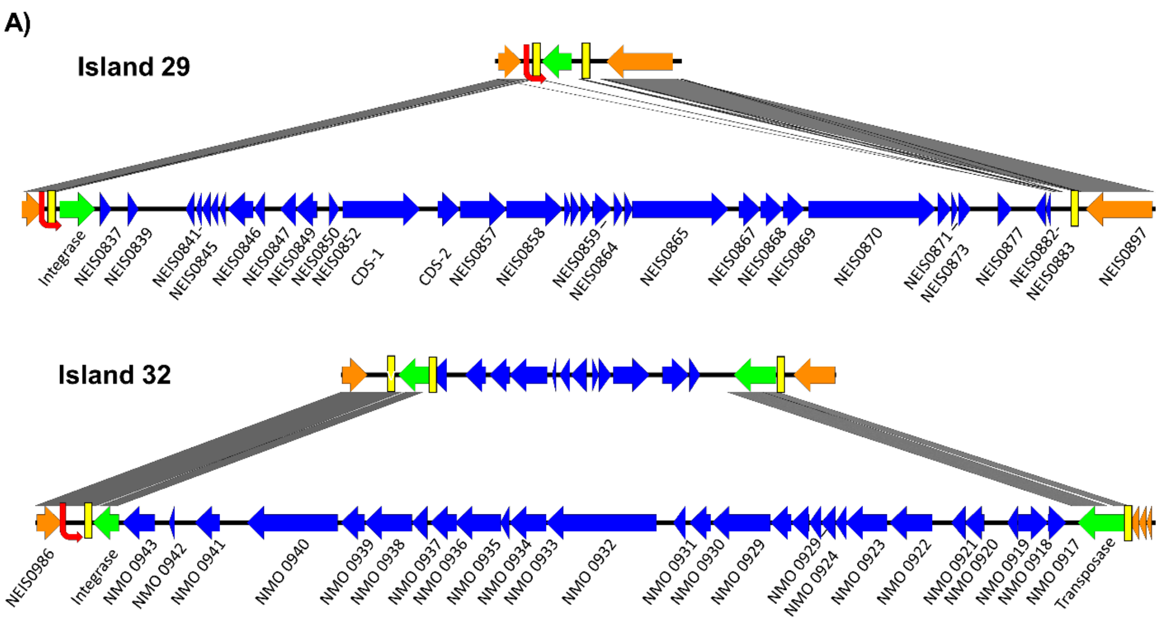

Supplementary Figure 5. Genomic islands that show evidence of a mechanism of horizontal transfer.

Genes within the island are depicted by blue arrows, green arrows represent transposable genetic elements, and the orange arrows represent the core genes flanking the island. tRNAs are depicted by red arrows. Direct repeats are shown in yellow boxes. The shaded grey regions between islands show regions of homology.

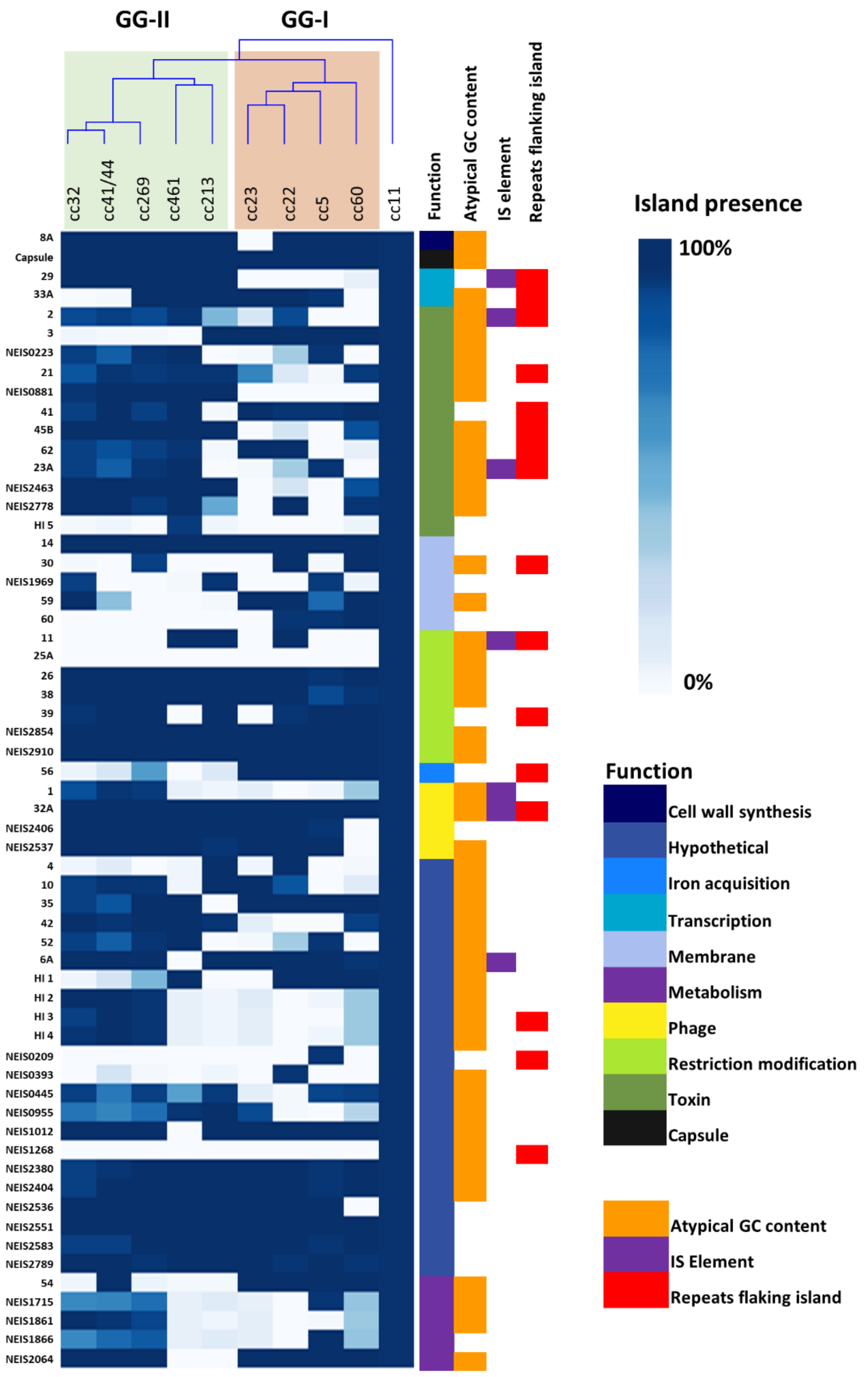

Supplementary Figure 6. Heatmap of hypervirulent associated genes (HVAGs) in 11 clonal complexes.

Each functional category has been assigned a different colour. Clonal complexes and are hierarchically clustered based on the presence of the pathogen associated islands and a corresponding dendrogram was generated. Genomic islands and single genes have been clustered according to putative function. Where a GI has been assigned a number, it has been listed next to the island. Island comprising hypothetical genes have been denoted as HI. Genomic islands with atypical GC content are coloured in orange, islands associated with an IS element are coloured in purple, and islands with flanking repeats are denoted in red. A dendrogram has been used to cluster clonal complexes into genogroup I (GG-I, orange) and II (GG-II, green).

Supplementary Table 1. Isolates used in this study from PubMLST.

PubMLST ID	Isolate name	Country of isolation	Collection date	Clonal complex
7	7891	Finland	1975	ST-5 complex
24	S4355	Denmark	1974	ST-5 complex
30	14	Germany	1999	ST-53 complex
82	11-004	China	1984	ST-5 complex
84	IAL2229	Brazil	1976	ST-5 complex
120	F4698	Saudi Arabia	1987	ST-5 complex
128	F6124	Chad	1988	ST-5 complex
210	H1964	UK	1987	ST-5 complex
237	H44/76	Norway	1976	ST-32 complex
238	153	China	1966	ST-5 complex
239	154	China	1966	ST-5 complex
240	MC58	UK	1983	ST-32 complex
299	80049	China	1963	ST-5 complex
314	D1	Mali	1989	ST-11 complex
340	196/87	Norway	1987	ST-32 complex
343	500	Italy	1984	ST-11 complex
349	38VI	USA	1964	ST-11 complex
369	M597	Israel	1988	ST-11 complex
391	90/18311	UK [Scotland]	1990	ST-11 complex
400	BZ 83	The Netherlands	1984	ST-32 complex
403	BZ 147	The Netherlands	1963	ST-41/44 complex
408	BZ 169	The Netherlands	1985	ST-32 complex
409	BZ 198	The Netherlands	1986	ST-41/44 complex
415	EG 329	Germany	1985	ST-32 complex
419	NG 6/88	Norway	1988	ST-269 complex

PubMLST ID	Isolate name	Country of isolation	Collection date	Clonal complex
420	NG F26	Norway	1988	ST-269 complex
421	NG H15	Norway	1988	ST-41/44 complex
427	NG E30	Norway	1988	ST-41/44 complex
428	NG H36	Norway	1988	ST-41/44 complex
430	NG 080	Norway	1981	ST-32 complex
431	NG144/82	Norway	1982	ST-32 complex
434	NG PB24	Norway	1985	ST-32 complex
441	8680	Chile	1987	ST-32 complex
451	14/1455	Russia	1970	ST-5 complex
468	BRAZ10	Brazil	1976	ST-11 complex
507	MA-5756	Spain	1985	ST-11 complex
597	92001	China	1992	ST-5 complex
644	L93/4286	UK [England]	1993	ST-11 complex
645	204/92	Cuba	1992	ST-32 complex
646	400	Austria	1991	ST-41/44 complex
647	AK50	Greece	1992	ST-41/44 complex
648	M-101/93	Iceland	1993	ST-41/44 complex
649	50/94	Norway	1994	ST-41/44 complex
650	M40/94	Chile	1994	ST-41/44 complex
651	931905	The Netherlands	1993	ST-41/44 complex
652	N45/96	Norway	1996	ST-41/44 complex
653	91/40	New Zealand	1991	ST-41/44 complex
659	A22	Norway	1986	ST-22 complex
660	71/94	Norway	1994	ST-23 complex
662	2837	UK	1997	ST-11 complex
665	2845	UK	1997	ST-11 complex
666	2843	UK	1997	ST-11 complex
667	2842	UK	1997	ST-11 complex

PubMLST ID	Isolate name	Country of isolation	Collection date	Clonal complex
669	2846	UK	1997	ST-11 complex
670	2840	UK	1997	ST-11 complex
671	2844	UK	1997	ST-11 complex
672	2847	UK	1997	ST-11 complex
684	0259/93	Czech Republic	1993	ST-11 complex
698	FAM18	USA	1983	ST-11 complex
939	0022/93	Czech Republic	1993	ST-11 complex
940	0023/93	Czech Republic	1993	ST-11 complex
942	0025/93	Czech Republic	1993	ST-11 complex
946	0033/93	Czech Republic	1993	ST-11 complex
948	0035/93	Czech Republic	1993	ST-11 complex
949	0036/93	Czech Republic	1993	ST-11 complex
950	0037/93	Czech Republic	1993	ST-11 complex
952	0039/93	Czech Republic	1993	ST-11 complex
954	0042/93	Czech Republic	1993	ST-11 complex
955	0043/93	Czech Republic	1993	ST-11 complex
957	0045/93	Czech Republic	1993	ST-11 complex
958	0046/93	Czech Republic	1993	ST-11 complex
968	0061/93	Czech Republic	1993	ST-22 complex
969	0062/93	Czech Republic	1993	ST-32 complex
972	0066/93	Czech Republic	1993	ST-53 complex
973	0067/93	Czech Republic	1993	ST-41/44 complex
975	0069/93	Czech Republic	1993	ST-41/44 complex
977	0071/93	Czech Republic	1993	ST-41/44 complex
978	0073/93	Czech Republic	1993	ST-11 complex
979	0074/93	Czech Republic	1993	ST-11 complex
982	0079/93	Czech Republic	1993	ST-11 complex
984	0083/93	Czech Republic	1993	ST-53 complex
991	0091/93	Czech Republic	1993	ST-41/44 complex

PubMLST ID	Isolate name	Country of isolation	Collection date	Clonal complex
992	0093/93	Czech Republic	1993	ST-11 complex
993	0095/93	Czech Republic	1993	ST-11 complex
994	0096/93	Czech Republic	1993	ST-11 complex
1168	BM33	Greece	1996	ST-32 complex
1169	BM45	Greece	1996	ST-11 complex
1170	BM45a	Greece	1996	ST-11 complex
1178	W72	Greece	1997	ST-11 complex
1179	W72a	Greece	1997	ST-11 complex
1180	W72b	Greece	1997	ST-11 complex
1181	W138	Greece	1998	ST-11 complex
1212	0099/93	Czech Republic	1993	ST-11 complex
1570	0101/93	Czech Republic	1993	ST-11 complex
1571	0102/93	Czech Republic	1993	ST-11 complex
1572	0103/93	Czech Republic	1993	ST-11 complex
1573	0104/93	Czech Republic	1993	ST-11 complex
1578	0109/93	Czech Republic	1993	ST-11 complex
1585	0117/93	Czech Republic	1993	ST-41/44 complex
1588	0120/93	Czech Republic	1993	ST-41/44 complex
1597	0206/93	Czech Republic	1993	ST-53 complex
1601	0211/93	Czech Republic	1993	ST-269 complex
1603	0213/93	Czech Republic	1993	ST-41/44 complex
1604	0214/93	Czech Republic	1993	ST-41/44 complex
1605	0215/93	Czech Republic	1993	ST-41/44 complex
1606	0216/93	Czech Republic	1993	ST-41/44 complex
1607	0217/93	Czech Republic	1993	ST-41/44 complex
1615	0225/93	Czech Republic	1993	ST-41/44 complex
1616	0226/93	Czech Republic	1993	ST-41/44 complex
1617	0227/93	Czech Republic	1993	ST-41/44 complex
1618	0228/93	Czech Republic	1993	ST-41/44 complex

PubMLST ID	Isolate name	Country of isolation	Collection date	Clonal complex
1633	0244/93	Czech Republic	1993	ST-41/44 complex
1639	0252/93	Czech Republic	1993	ST-53 complex
1640	0253/93	Czech Republic	1993	ST-41/44 complex
1642	0255/93	Czech Republic	1993	ST-41/44 complex
1645	0258/93	Czech Republic	1993	ST-41/44 complex
1649	0269/93	Czech Republic	1993	ST-53 complex
1654	0277/93	Czech Republic	1993	ST-53 complex
1656	0280/93	Czech Republic	1993	ST-41/44 complex
1949	0306/93	Czech Republic	1993	ST-22 complex
1955	0011/93	Czech Republic	1993	ST-41/44 complex
1958	0307/93	Czech Republic	1993	ST-41/44 complex
2077	153	Germany	1999	ST-60 complex
2216	0190/93	Czech Republic	1993	ST-269 complex
2220	0312/93	Czech Republic	1993	ST-22 complex
2222	0314/93	Czech Republic	1993	ST-53 complex
2233	0531/93	Czech Republic	1993	ST-32 complex
8139	0320/93	Czech Republic	1993	ST-41/44 complex
8144	0325/93	Czech Republic	1993	ST-41/44 complex
8156	0342/93	Czech Republic	1993	ST-41/44 complex
8164	0404/93	Czech Republic	1993	ST-41/44 complex
8168	0409/93	Czech Republic	1993	ST-41/44 complex
8169	0410/93	Czech Republic	1993	ST-53 complex
8171	0412/93	Czech Republic	1993	ST-41/44 complex
8173	0414/93	Czech Republic	1993	ST-41/44 complex
8176	0421/93	Czech Republic	1993	ST-41/44 complex
8177	0422/93	Czech Republic	1993	ST-53 complex
8181	0426/93	Czech Republic	1993	ST-53 complex
9756	275	Germany	2000	ST-22 complex
10705	N 25/99	Norway	1999	ST-41/44 complex

PubMLST ID	Isolate name	Country of isolation	Collection date	Clonal complex
10735	N 56/99	Norway	1999	ST-41/44 complex
10756	8/00	Norway	2000	ST-41/44 complex
10758	10/00	Norway	2000	ST-41/44 complex
10812	64/00	Norway	2000	ST-41/44 complex
10830	MK 66/96	Norway	1996	ST-41/44 complex
10837	MK 75/96	Norway	1996	ST-41/44 complex
14480	M5178	USA	1998	ST-32 complex
15249	0379/93	Czech Republic	1993	ST-32 complex
15313	0469/93	Czech Republic	1993	ST-32 complex
18968	M10 240474	UK [England]	2010	ST-269 complex
19023	M10 240473	UK [England]	2010	ST-269 complex
19028	M10 240485	UK [England]	2010	ST-269 complex
19029	M10 240487	UK [England]	2010	ST-269 complex
19030	M10 240489	UK [England]	2010	ST-461 complex
19031	M10 240490	UK [England]	2010	ST-269 complex
19260	WUE 2594	Germany	1991	ST-5 complex
19264	M04-240196	UK	2004	ST-269 complex
19265	M01-240355	UK	2001	ST-213 complex
19359	M13399	Unknown	2005	ST-269 complex
19362	CU385	Unknown	1980	ST-32 complex
19366	M10699	USA	2003	ST-32 complex
19368	M13220	Philippines	2005	ST-5 complex
19372	M15293	USA	2006	ST-32 complex
19958	M10 240476	UK [England]	2010	ST-213 complex
19959	M10 240499	UK [England]	2010	ST-269 complex
19962	M10 240503	UK [England]	2010	ST-23 complex
19963	M10 240505	UK [England]	2010	ST-23 complex
19964	M10 240507	UK [England]	2010	ST-23 complex
19969	M10 240515	UK [England]	2010	ST-269 complex

PubMLST ID	Isolate name	Country of isolation	Collection date	Clonal complex
19970	M10 240520	UK [England]	2010	ST-23 complex
19973	M10 240525	UK [England]	2010	ST-32 complex
19974	M10 240527	UK [England]	2010	ST-269 complex
19975	M10 240528	UK [England]	2010	ST-269 complex
19976	M10 240530	UK [England]	2010	ST-23 complex
19977	M10 240531	UK [England]	2010	ST-23 complex
19979	M10 240534	UK [England]	2010	ST-23 complex
19980	M10 240536	UK [England]	2010	ST-23 complex
19981	M10 240540	UK [England]	2010	ST-23 complex
19982	M10 240546	UK [England]	2010	ST-213 complex
19983	M10 240547	UK [England]	2010	ST-32 complex
19986	M10 240550	UK [England]	2010	ST-269 complex
19987	M10 240553	UK [Wales]	2010	ST-269 complex
19991	M10 240566	UK [England]	2010	ST-213 complex
19992	M10 240568	UK [England]	2010	ST-213 complex
19995	M10 240572	UK [England]	2010	ST-269 complex
19996	M10 240578	UK [England]	2010	ST-32 complex
19998	M10 240580	UK [England]	2010	ST-23 complex
20000	M10 240582	UK [England]	2010	ST-32 complex
20006	M10 240590	UK [England]	2010	ST-23 complex
20007	M10 240591	UK [England]	2010	ST-461 complex
20009	M10 240595	UK [England]	2010	ST-213 complex
20012	M10 240602	UK [England]	2010	ST-269 complex
20015	M10 240606	UK [England]	2010	ST-213 complex
20017	M10 240611	UK [England]	2010	ST-32 complex
20018	M10 240612	UK [England]	2010	ST-269 complex
20019	M10 240613	UK [England]	2010	ST-269 complex
20021	M10 240616	UK [England]	2010	ST-22 complex
20022	M10 240617	UK [England]	2010	ST-213 complex

PubMLST ID	Isolate name	Country of isolation	Collection date	Clonal complex
20023	M10 240618	UK [England]	2010	ST-60 complex
20024	M10 240619	UK [England]	2010	ST-213 complex
20025	M10 240621	UK [Wales]	2010	ST-213 complex
20027	M10 240623	UK [England]	2010	ST-269 complex
20028	M10 240624	UK [England]	2010	ST-269 complex
20029	M10 240626	UK [England]	2010	ST-269 complex
20030	M10 240627	UK [England]	2010	ST-32 complex
20031	M10 240631	UK [England]	2010	ST-269 complex
20032	M10 240632	UK [England]	2010	ST-23 complex
20033	M10 240633	UK [England]	2010	ST-23 complex
20036	M10 240638	UK [England]	2010	ST-269 complex
20039	M10 240641	UK [England]	2010	ST-22 complex
20041	M10 240643	UK [England]	2010	ST-23 complex
20042	M10 240644	UK [England]	2010	ST-22 complex
20043	M10 240645	UK [England]	2010	ST-269 complex
20044	M10 240649	UK [England]	2010	ST-269 complex
20046	M10 240651	UK [England]	2010	ST-213 complex
20047	M10 240652	UK [England]	2010	ST-269 complex
20050	M10 240661	UK [England]	2010	ST-269 complex
20052	M10 240665	UK [England]	2010	ST-213 complex
20056	M10 240670	UK [England]	2010	ST-269 complex
20064	M10 240682	UK [Northern Ireland]	2010	ST-269 complex
20067	M10 240685	UK [England]	2010	ST-23 complex
20068	M10 240687	UK [England]	2010	ST-60 complex
20071	M10 240693	UK [England]	2010	ST-60 complex
20074	M10 240700	UK [England]	2010	ST-23 complex
20075	M10 240701	UK [England]	2010	ST-213 complex
20077	M10 240703	UK [England]	2010	ST-213 complex

PubMLST ID	Isolate name	Country of isolation	Collection date	Clonal complex
20078	M10 240704	UK [England]	2010	ST-269 complex
20081	M10 240708	UK [England]	2010	ST-269 complex
20082	M10 240709	UK [England]	2010	ST-269 complex
20083	M10 240711	UK [England]	2010	ST-269 complex
20084	M10 240712	UK [England]	2010	ST-213 complex
20085	M10 240713	UK [England]	2010	ST-269 complex
20086	M10 240716	UK [England]	2010	ST-461 complex
20087	M10 240717	UK [England]	2010	ST-213 complex
20088	M10 240718	UK [England]	2010	ST-60 complex
20089	M10 240719	UK [England]	2010	ST-32 complex
20090	M10 240720	UK [England]	2010	ST-269 complex
20095	M10 240728	UK [England]	2010	ST-269 complex
20096	M10 240729	UK [England]	2010	ST-269 complex
20099	M10 240732	UK [England]	2010	ST-23 complex
20101	M10 240737	UK [England]	2010	ST-22 complex
20102	M10 240742	UK [England]	2010	ST-213 complex
20103	M10 240743	UK [England]	2010	ST-269 complex
20104	M10 240745	UK [England]	2010	ST-23 complex
20110	M10 240751	UK [England]	2010	ST-213 complex
20111	M10 240752	UK [England]	2010	ST-269 complex
20112	M10 240753	UK [England]	2010	ST-32 complex
20114	M10 240756	UK [England]	2010	ST-22 complex
20119	M10 240766	UK [England]	2010	ST-269 complex
20120	M10 240767	UK [Wales]	2010	ST-32 complex
20121	M10 240768	UK [England]	2010	ST-269 complex
20122	M10 240770	UK [England]	2010	ST-269 complex
20124	M10 240772	UK [England]	2010	ST-269 complex
20126	M10 240774	UK [Wales]	2010	ST-269 complex
20128	M10 240776	UK [England]	2010	ST-23 complex

PubMLST ID	Isolate name	Country of isolation	Collection date	Clonal complex
20129	M10 240777	UK [England]	2010	ST-23 complex
20130	M10 240778	UK [Wales]	2010	ST-213 complex
20131	M10 240779	UK [England]	2010	ST-269 complex
20132	M10 240781	UK [England]	2010	ST-22 complex
20133	M10 240783	UK [England]	2010	ST-269 complex
20135	M10 240786	UK [England]	2010	ST-23 complex
20136	M10 240787	UK [England]	2010	ST-23 complex
20138	M10 240789	UK [Wales]	2010	ST-269 complex
20139	M10 240790	UK [England]	2010	ST-32 complex
20141	M10 240794	UK [England]	2010	ST-32 complex
20143	M10 240798	UK [England]	2010	ST-23 complex
20144	M10 240799	UK [England]	2010	ST-213 complex
20146	M10 240804	UK [England]	2010	ST-23 complex
20159	M10 240822	UK [England]	2010	ST-22 complex
20161	M10 240824	UK [England]	2010	ST-32 complex
20162	M10 240825	UK [England]	2010	ST-60 complex
20164	M11 240000	UK [England]	2011	ST-23 complex
20165	M11 240001	UK [England]	2011	ST-461 complex
20169	M11 240005	UK [England]	2011	ST-23 complex
20170	M11 240006	UK [Wales]	2011	ST-32 complex
20171	M11 240007	UK [England]	2011	ST-23 complex
20176	M11 240013	UK [England]	2011	ST-32 complex
20177	M11 240014	UK [England]	2011	ST-461 complex
20179	M11 240016	UK [Wales]	2011	ST-32 complex
20183	M11 240021	UK [England]	2011	ST-32 complex
20189	M11 240027	UK [England]	2011	ST-60 complex
20190	M11 240028	UK [England]	2011	ST-60 complex
20191	M11 240029	UK [England]	2011	ST-22 complex
20193	M11 240031	UK [England]	2011	ST-23 complex

PubMLST ID	Isolate name	Country of isolation	Collection date	Clonal complex
20197	M11 240036	UK [Wales]	2011	ST-23 complex
20198	M11 240037	UK [England]	2011	ST-32 complex
20203	M11 240042	UK [England]	2011	ST-461 complex
20204	M11 240043	UK [England]	2011	ST-23 complex
20207	M11 240046	UK [England]	2011	ST-213 complex
20209	M11 240048	UK [England]	2011	ST-60 complex
20210	M11 240050	UK [England]	2011	ST-213 complex
20211	M11 240052	UK [Wales]	2011	ST-461 complex
20220	M11 240061	UK [England]	2011	ST-213 complex
20224	M11 240065	UK [England]	2011	ST-23 complex
20233	M11 240074	UK [Wales]	2011	ST-213 complex
20238	M11 240082	UK [England]	2011	ST-32 complex
20239	M11 240083	UK [England]	2011	ST-461 complex
20245	M11 240096	UK [England]	2011	ST-23 complex
20250	M11 240109	UK [England]	2011	ST-32 complex
20252	M11 240111	UK [England]	2011	ST-213 complex
20254	M11 240113	UK [England]	2011	ST-213 complex
20256	M11 240116	UK [England]	2011	ST-23 complex
20257	M11 240117	UK [England]	2011	ST-60 complex
20259	M11 240121	UK [England]	2011	ST-213 complex
20264	M11 240126	UK [England]	2011	ST-23 complex
20270	M11 240134	UK [England]	2011	ST-213 complex
20271	M11 240137	UK [England]	2011	ST-213 complex
20275	M11 240147	UK [England]	2011	ST-213 complex
20281	M11 240157	UK [England]	2011	ST-23 complex
20286	M11 240166	UK [England]	2011	ST-23 complex
20287	M11 240167	UK [England]	2011	ST-60 complex
20292	M11 240175	UK [England]	2011	ST-32 complex
20295	M11 240181	UK [England]	2011	ST-213 complex

PubMLST ID	Isolate name	Country of isolation	Collection date	Clonal complex
20297	M11 240183	UK [England]	2011	ST-32 complex
20302	M11 240192	UK [Wales]	2011	ST-23 complex
20306	M11 240206	UK [England]	2011	ST-461 complex
20309	M11 240210	UK [England]	2011	ST-23 complex
20313	M11 240214	UK [England]	2011	ST-461 complex
20314	M11 240215	UK [England]	2011	ST-23 complex
20315	M11 240216	UK [England]	2011	ST-23 complex
20317	M11 240227	UK [England]	2011	ST-23 complex
20318	M11 240231	UK [England]	2011	ST-22 complex
20328	M11 240246	UK [England]	2011	ST-60 complex
20335	M11 240258	UK [England]	2011	ST-23 complex
20337	M11 240261	UK [England]	2011	ST-22 complex
20338	M11 240262	UK [England]	2011	ST-5 complex
20339	M11 240263	UK [England]	2011	ST-23 complex
20341	M11 240266	UK [England]	2011	ST-213 complex
20342	M11 240268	UK [England]	2011	ST-213 complex
20346	M11 240277	UK [England]	2011	ST-23 complex
20347	M11 240278	UK [England]	2011	ST-23 complex
20350	M11 240282	UK [England]	2011	ST-213 complex
20351	M11 240283	UK [England]	2011	ST-23 complex
20353	M11 240285	UK [England]	2011	ST-32 complex
20357	M11 240290	UK [England]	2011	ST-213 complex
20361	M11 240296	UK [England]	2011	ST-32 complex
20362	M11 240297	UK [England]	2011	ST-213 complex
20363	M11 240298	UK [England]	2011	ST-23 complex
20365	M11 240302	UK [England]	2011	ST-22 complex
20372	M11 240312	UK [England]	2011	ST-23 complex
20373	M11 240313	UK [England]	2011	ST-23 complex
20382	M11 240324	UK [England]	2011	ST-32 complex

PubMLST ID	Isolate name	Country of isolation	Collection date	Clonal complex
20389	M11 240337	UK [England]	2011	ST-213 complex
20391	M11 240339	UK [England]	2011	ST-60 complex
20393	M11 240342	UK [England]	2011	ST-32 complex
20395	M11 240344	UK [England]	2011	ST-213 complex
20398	M11 240348	UK [England]	2011	ST-213 complex
20399	M11 240349	UK [England]	2011	ST-22 complex
20403	M11 240356	UK [England]	2011	ST-32 complex
20409	M11 240366	UK [Northern Ireland]	2011	ST-32 complex
20422	M11 240387	UK [England]	2011	ST-22 complex
20427	M11 240392	UK [England]	2011	ST-32 complex
20428	M11 240394	UK [England]	2011	ST-60 complex
20438	M11 240406	UK [England]	2011	ST-32 complex
20441	M11 240412	UK [England]	2011	ST-60 complex
20457	M11 240440	UK [England]	2011	ST-60 complex
20477	H44/76	Norway	1976	ST-32 complex
20564	Nm6938	Canada	2001	ST-22 complex
20565	Nm2732	Canada	2007	ST-22 complex
20741	M09 240064	UK	2013	ST-60 complex
20753	M09 240242	UK	2013	ST-461 complex
20759	M09 240296	UK	2013	ST-213 complex
20763	M09 240440	UK	2013	ST-213 complex
20769	M09 240659	UK	2013	ST-213 complex
20771	M09 240681	UK	2013	ST-213 complex
20790	M10 240127	UK	2013	ST-60 complex
20793	M10 240140	UK	2013	ST-461 complex
20899	M05 240362	UK	2013	ST-60 complex
20900	M05 240370	UK	2013	ST-60 complex
20903	M05 241347	UK	2013	ST-22 complex

PubMLST ID	Isolate name	Country of isolation	Collection date	Clonal complex
20904	M05 241348	UK	2013	ST-22 complex
20912	M05 241259	UK	2013	ST-213 complex
20921	M03 240607	UK	2013	ST-213 complex
20922	M03 240609	UK	2013	ST-213 complex
20923	M03 240829	UK	2013	ST-213 complex
20924	M03 240830	UK	2013	ST-213 complex
20925	M03 241030	Ireland	2013	ST-213 complex
20926	M03 241054	Ireland	2013	ST-213 complex
20971	M08 240422	Isle of Man	2008	ST-60 complex
20972	M08 240423	Isle of Man	2013	ST-60 complex
21092	M11 240443	UK [England]	2011	ST-461 complex
21112	M11 240471	UK [England]	2011	ST-60 complex
21132	M11 240497	UK [England]	2011	ST-22 complex
21139	M11 240508	UK [England]	2011	ST-22 complex
21156	M11 240716	UK [England]	2011	ST-22 complex
21175	M11 240741	UK [England]	2011	ST-22 complex
21193	M11 240780	UK [England]	2011	ST-22 complex
21228	M11 240988	UK [England]	2011	ST-461 complex
21246	M11 241031	UK [England]	2011	ST-461 complex
21271	M11 241064	UK [England]	2011	ST-22 complex
21280	M11 241075	UK [Wales]	2011	ST-60 complex
21290	M12 240008	UK [England]	2012	ST-22 complex
21300	M12 240019	UK [England]	2012	ST-461 complex
21349	M12 240088	UK [England]	2012	ST-22 complex
21352	M12 240093	UK [England]	2012	ST-60 complex
21371	M12 240121	UK [England]	2012	ST-22 complex
21380	M12 240132	UK [England]	2012	ST-22 complex
21396	M12 240158	UK [England]	2012	ST-60 complex
21432	M12 240222	UK [England]	2012	ST-60 complex

PubMLST ID	Isolate name	Country of isolation	Collection date	Clonal complex
21454	M12 240251	UK [England]	2012	ST-22 complex
21576	SA_serogroup W_NM4	South Africa	2012	ST-22 complex
26067	959000095	Sweden	1995	ST-22 complex
26253	T97	UK	2011	ST-53 complex
26263	V259	UK	2011	ST-53 complex
26264	V302	UK	2012	ST-60 complex
26267	T98	UK	2011	ST-53 complex
26271	BB66	UK	2012	ST-53 complex
26273	BB58	UK	2012	ST-22 complex
26274	v191	UK	2012	ST-53 complex
26275	BB242	UK	2012	ST-22 complex
26278	BB122	UK	2012	ST-60 complex
26283	R121	UK	2011	ST-53 complex
26287	V129	UK	2011	ST-22 complex
26297	T36	UK	2011	ST-60 complex
26303	T287	UK	2011	ST-22 complex
26306	R279	UK	2011	ST-60 complex
26308	x268	UK	2012	ST-60 complex
26314	z54	UK	2012	ST-60 complex
26320	R136	UK	2011	ST-53 complex
26325	x83	UK	2012	ST-53 complex
26326	T304	UK	2011	ST-53 complex
26327	R122	UK	2011	ST-60 complex
26329	BB130	UK	2012	ST-60 complex
26332	BB111	UK	2012	ST-461 complex
26336	v216	UK	2012	ST-53 complex
26344	R243	UK	2011	ST-53 complex
26349	BB279	UK	2012	ST-60 complex

PubMLST ID	Isolate name	Country of isolation	Collection date	Clonal complex
26350	T3	UK	2011	ST-53 complex
26352	BB36	UK	2012	ST-60 complex
26355	R263	UK	2011	ST-53 complex
26360	R221	UK	2011	ST-60 complex
26365	z191	UK	2012	ST-53 complex
26370	R129	UK	2011	ST-22 complex
26372	V304	UK	2012	ST-53 complex
26381	R210	UK	2011	ST-22 complex
26382	X136	UK	2011	ST-53 complex
26383	R216	UK	2011	ST-53 complex
26390	BB210	UK	2012	ST-22 complex
26394	T121	UK	2011	ST-53 complex
26399	R155	UK	2011	ST-53 complex
26402	R83	UK	2011	ST-53 complex
26406	T122	UK	2011	ST-60 complex
26408	BB287	UK	2012	ST-22 complex
26409	R222	UK	2011	ST-60 complex
26411	R36	UK	2011	ST-60 complex
26414	R4	UK	2011	ST-60 complex
26415	T191	UK	2011	ST-53 complex
26418	x222	UK	2012	ST-60 complex
26419	T119	UK	2011	ST-53 complex
26425	R256	UK	2011	ST-60 complex
26432	T210	UK	2011	ST-22 complex
26434	T302	UK	2011	ST-60 complex
26444	R97	UK	2011	ST-53 complex
26445	Z119	UK	2012	ST-53 complex
26450	BB159	UK	2012	ST-60 complex
26451	r119	UK	2011	ST-53 complex

PubMLST ID	Isolate name	Country of isolation	Collection date	Clonal complex
26457	R191	UK	2011	ST-53 complex
26464	Z221	UK	2012	ST-60 complex
26466	X119	UK	2012	ST-53 complex
26474	T216	UK	2011	ST-53 complex
26476	R58	UK	2011	ST-22 complex
26498	T279	UK	2011	ST-60 complex
26500	BB83	UK	2012	ST-53 complex
26515	BB54	UK	2012	ST-60 complex
26518	v119	UK	2012	ST-53 complex
26519	T53	UK	2011	ST-60 complex
26522	x121	UK	2012	ST-53 complex
26529	BB228	UK	2012	ST-53 complex
26530	V210	UK	2012	ST-22 complex
26839	12005_2011	Ireland	2011	ST-461 complex
26847	12018_2011	Ireland	2011	ST-461 complex
26888	12029_2012	Ireland	2012	ST-22 complex
26900	12004_2013	Ireland	2013	ST-22 complex
26922	10215v3	UK	2013	ST-22 complex
26923	10215v4	UK	2013	ST-22 complex
27782	M12 240347	UK [England]	2012	ST-461 complex
27814	M12 240704	UK [England]	2012	ST-461 complex
27839	M12 240763	UK [England]	2012	ST-461 complex
27843	M12 240778	UK [England]	2012	ST-461 complex
27849	M12 240787	UK [Wales]	2012	ST-461 complex
27864	M12 240819	UK [England]	2012	ST-461 complex
27871	M12 240844	UK [England]	2012	ST-461 complex
27887	M12 240873	UK [Wales]	2012	ST-461 complex
27907	M13 240006	UK [England]	2013	ST-461 complex
27914	M13 240021	UK [England]	2013	ST-461 complex

PubMLST ID	Isolate name	Country of isolation	Collection date	Clonal complex
27927	M13 240045	UK [England]	2013	ST-461 complex
27933	M13 240057	UK [England]	2013	ST-461 complex
27934	M13 240063	UK [England]	2013	ST-461 complex
27938	M13 240074	UK [England]	2013	ST-461 complex
27941	M13 240078	UK [England]	2013	ST-461 complex
28017	M13 240248	UK [England]	2013	ST-461 complex
28043	M13 240406	UK [England]	2013	ST-461 complex
29291	15003	South Africa	2004	ST-5 complex
29304	27937	South Africa	2010	ST-5 complex
29312	5957	South Africa	2006	ST-53 complex
29434	139	South Africa	2005	ST-5 complex
30269	M99 243594	UK	1999	ST-5 complex
30466	NMA510612	China	2006	ST-5 complex
30697	12011-10	Ireland	2010	ST-461 complex
30708	12021-13	Ireland	2013	ST-461 complex
30760	M14.1	Ireland	2013	ST-461 complex
31208	NM10853	UK [England]	2013	ST-53 complex
31215	LIM534	France	2006	ST-5 complex
34572	75643	Brazil	1975	ST-5 complex
34573	75689	Brazil	1975	ST-5 complex
34578	88050	Chad	1988	ST-5 complex
34583	96023	Niger	1996	ST-5 complex
34584	96024	Niger	1996	ST-5 complex
34587	97008	Niger	1997	ST-5 complex
34589	97018	Mali	1997	ST-5 complex
34590	97020	Mali	1997	ST-5 complex
34594	98005	Niger	1998	ST-5 complex
34595	98008	France	1998	ST-461 complex
34597	2000063	Niger	2000	ST-5 complex

PubMLST ID	Isolate name	Country of isolation	Collection date	Clonal complex
34598	2000080	Algeria	2000	ST-5 complex
34599	2001212	Burkina Faso	2001	ST-5 complex
34600	2002007	Niger	2002	ST-5 complex
34604	2003022	Niger	2003	ST-5 complex
34606	2004085	Niger	2004	ST-5 complex
34607	2004090	Niger	2004	ST-5 complex
34610	2007056	Burkina Faso	2007	ST-5 complex
34654	NM3642	Bangladesh	2003	ST-5 complex
34655	NM3652	Bangladesh	2006	ST-5 complex
34663	NM604	USA	2000	ST-5 complex
34665	NM607	USA	2001	ST-5 complex
34805	ERR051676	Ghana	2008	ST-5 complex
34806	ERR051677	Ghana	2009	ST-5 complex
34807	ERR051678	Ghana	2009	ST-5 complex
34808	ERR052799	Burkina Faso	2006	ST-5 complex
34809	ERR052812	Burkina Faso	2006	ST-5 complex
34810	ERR052817	Burkina Faso	2006	ST-5 complex
34811	ERR052816	Burkina Faso	2007	ST-5 complex
34812	ERR052813	Burkina Faso	2006	ST-5 complex
35330	12.1192.H	UK [Scotland]	2012	ST-461 complex
35345	13.2973.F	UK [Scotland]	2013	ST-461 complex
35346	13.3065.D	UK [Scotland]	2013	ST-461 complex
35427	M13 240534	UK [England]	2013	ST-461 complex
35436	M13 240554	UK [England]	2013	ST-461 complex
35448	M13 240581	UK [England]	2013	ST-461 complex
35480	M13 240644	UK [England]	2013	ST-461 complex
35577	M14 240083	UK [Northern Ireland]	2014	ST-461 complex
35580	M14 240088	UK [England]	2014	ST-461 complex

PubMLST ID	Isolate name	Country of isolation	Collection date	Clonal complex
35612	M14 240133	UK [Wales]	2014	ST-461 complex
36135	12010_2012	Ireland	2012	ST-53 complex
38889	P27	Italy	2012	ST-53 complex
38943	P106	Italy	2013	ST-53 complex
38946	P109	Italy	2013	ST-53 complex
38953	P117	Italy	2013	ST-53 complex

Supplementary Table 2. Genomic islands from the pan-accessory genome of *N. meningitidis* identified in this study.

Island number/function	NEIS Code	BLASTP ID	GC%	GC percent different from mean	Closest non-Neisseria hit (ID and origin)	BLASTP %ID of non-Neisseria hit	Repeats flanking island	tRNA	Reference
Island 1 (MDA-ϕ) Phage		**IS110**					DR	No	Proximal core gene is *pilA* (NEIS0021) (Bille et al., 2008)
	NEIS0023	Zonular occludens toxin (Zot) family protein	44.53	Yes	Hypothetical protein - *Helicobacter pylori*	99%			
	NEIS0024	Putative integral membrane protein	44.91	Yes	Hypothetical protein - *Kingella sp.*	45%			
	NEIS0025	T cell/B cell stimulating protein TspB	47.69	Yes	None				
	NEIS0027	Membrane protein (*Neisseria meningtidis)*	43.67	Yes	Hypothetical protein - *Helicobacter pylori*	96%			
	NEIS0028	Membrane protein (*N. meningitidis*)	54.63	Yes	Membrane protein - *Proteobacteria*	93%			
	NEIS0029	Hypothetical protein	53.84	No	Hypothetical protein - *Kingella kingae*	51%			
	NEIS0030	Hypothetical protein	53.58	No	Hypothetical protein - *Helicobacter pylori*	97%			
	NEIS0031	Replication initiation factor (*N.meningitidis)*	52.23	No	None				
Island 2 (Bacteriocin encoding GI-BGI 1) Toxin		IS1016							Proximal core gene is *pykA* (NEIS0074) (Allunans et al., 2008)
	NEIS0080	Bacteriocin transporter (*N.meningitidis)*	38.4	Yes	Hypothetical protein - *Simonsiella muelleri*	76%	IR	No	
	NEIS0081	Hypothetical protein (*N.meningitidis)*	33.77	Yes	Hypothetical protein - *Simonsiella muelleri*	70%			
	NEIS0083	Multispecies membrane protein (*Nesseria)*	22.87	Yes	NADH dehydrogenase - *Leptospira santarosai*	40%			
	NEIS0084	Membrane protein (*Neisseria meningitidis)*	26.81	Yes	None				
	NEIS0085	Colicin V secretion protein (*Neisseria)*	37.44	Yes	Transporter protein - *Acinetobacter sp*	47%			

	NEIS0086	Colicin V processing peptidase (*Neisseria meningitdis)* Phase variable	34.24	Yes	Colicin V processing peptidase - *Acinetobacter sp*	56%			
	NEIS0089	Hypothetical protein	34.69	Yes	None				
		IS1016							
Island 3 (Abi-like) Metabolism	NEIS0169	Abi-like family protein (bacteriocin self-immunity protein)	35.41	Yes	CAAX protease - *Mesonia mobilis*	80%	No	No	Proximal core gene is *lpxA* (NEIS0168) (Kjos et al., 2010)
Island 4 Hypothetical	NEIS0193	Hypothetical protein(*Neisseria meningitidis)*	28.72	Yes	Hypothetical protein - *Bibersteinia trehalosi*	34%	No	No	Proximal core gene is *lpxB* (NEIS0191)
Island 5 (Pgl-1) Membrane	NEIS2516	Hypothetical protein	50	No	Gylcosyltransferase *N. gonorrhoeae* (all other hits are for Neisseria)	86%	IR	No	Proximal core gene is *pglA* (NEIS0213) (Dinh et al., 1994, Yan, 2013)
	NEIS0214	Major Facilitator Superfamily transporter (*Neisseria meningitdis)*	60.34	Yes	MFS transporter - *Kingella dentrificans*	58%			
	NEIS0215	bile acid:sodium symporter*(Neisseria meningitdis)*	57.26	Yes	bile acid:sodium symporter - *Alysiella crassa*	80%	DR		
Island 6A (SMI1/KNR4) Cell wall synthesis	NEIS0231	SMI1 / KNR4 family protein (cell division co-ordination)	45.93	Yes	SMI1 / KNR4 family protein - *Chryseobacterium sp*	53%	No	No	Proximal core gene is NEIS0230 (Basmaji et al., 2006)
	NEIS0232	Hypothetical protein	30.56	Yes	pyruvate, phosphate dikinase - *Propionibacterium acnes*	40%			
	NEIS0233	Hypothetical protein	27.5	Yes	Immunity protein 47 family protein - *Serratia symbiotica*	36%			
	CDS	septum formation inhibitor Maf	36.6	Yes	Transposase - *Kingella kingae*	92%			
		IS1595							
Island 6B Membrane	CDS	Membrane protein	41.67	Yes	Membrane protein - *Actinobacillus muris*	42%	No	No	See above
	CDS	IS5							

Island 7A Restriction Modification	NEIS2477	putative type II restriction endonuclease DpnC (dam replacing protein)	42.8	Yes	Hypothetical protein - *Eikenella corrodens*	60%	No	No	Proximal core gene is NEIS0326 (Jolley et al., 2004)
Island 7B Metabolism	NEIS2475	Zn-dependent protease with chaperone function	54	No	deoxyribonuclease HsdR - *Stenoxybacter acetivorans*	54%	No	No	Proximal core gene is NEIS0326
Island 8A (NmFic) Cell wall synthesis	NEIS0249	adenosine monophosphate-protein transferase NmFic (Neisseria)	46.01	Yes	Fic Family protein - *Aggregatibacter sp*	85%	No	No	Proximal core gene is NEIS0258 (Stanger et al., 2016)
	NEIS0250	Hypothetical protein	39.77	Yes	Hypothetical protein – *Vibrio gazogenes*	51%			
Island 8B Membrane	NEIS2517	Putative permease	35.97	Yes	permease - *Dickeya zeae*	48%	No	No	Proximal core gene is NEIS0258
Island 9 Cell wall synthesis	NEIS2518	HKD family nuclease	33.93	Yes	Hypothetical protein - *Vibrio harveyi*	47%	No	No	Proximal core gene isNEIS0251
Island 10 Membrane	NEIS0267	Membrane protein	22.22	Yes	Membrane protein *Kingella kingae*	43%	No	No	Proximal core gene is NEIS0265
Island 11 Restriction modification	NEIS0295	Modification methylase HgaIA	38.32	Yes	restriction endonuclease Eco57I *Flavobacterium psychrophilum*	63%	IR	No	Proximal core gene is NEIS0294 (Claus et al., 2000)
	CDS	Hin4II restriction endonuclease	34.83	Yes	hypothetical protein *Brachyspira hyodysenteriae*	47%			
	NEIS0298	Hypothetical protein	33.33	Yes	Hypothetical protein *Gilliamella apicola*	51%			
		IS5							
Island 12 (Dam) Restriction modification	NEIS0327	DNA adenine methylase	41.08	Yes	DNA adenine methylase - *Kingella oralis*	68%	No	No	Proximal core gene is NEIS0326 (Jolley et al., 2004)
	NEIS0328	modification methylase DpnIIB	39.8	Yes	DNA methylase N-4 *Kingella oralis*	78%			
	NEIS0329	hypothetical protein	34.95	Yes	Hypothetical protein - *Alysiella crassa*	72%			
Island 13 Hypothetical	NEIS2506	Hypothetical protein	29.44	Yes	Hypothetical protein - *Aggregatibacter aphrophilus*	52%	No	No	Proximal core gene is NEIS0367

Island 14 (PglI) Membrane	NEIS0380	PglI	53.61	No	None		No	No	Proximal gene NEIS0381 (tyrosyl-tRNA synthetase) (Warren et al., 2004)
Island 15A (BGI-2) Toxin	CDS	Initiator RepB protein	36.34	Yes	Initiator RepB protein - *Moraxella bovoculi*	70%	No	No	Proximal core gene is NEIS0388
			0	Yes					
	CDS	Cupin Superfamily protein	32.74	Yes	Cupin - *Acinetobacter baumannii*	52%			
	CDS	Cupin Superfamily protein	36.86	Yes	Cupin - *Simonsiella muelleri*	68%			
	NEIS0891	Cupin	32.58		cupin - *Simonsiella muelleri*	65%			
	CDS	Bacteriocin transporter	39.29	Yes	hypothetical protein - *Acinetobacter guillouiae*	34%			
	NEIS0890	Membrane protein	18.1	Yes	Organic cation transporter like protein - *Papilio polytes*	29%			
	NEIS0889	Membrane protein	46.13	Yes	hypothetical protein - *Oceanospirillum maris*	41%			
	CDS	Bacteriocin transporter	39.4	Yes	hypothetical protein - *Acinetobacter guillouiae*	34%			
	CDS	putative bacteriocin processing protease	45.21	Yes	ABC transporter - *Burkholderia ubonensis*	52%			
			0	Yes					
		IS1016							
Island 15B (CRISPR Cas9) Restriction modification	NEIS2568	CRISPR protein Cas2	44.34	Yes	subtype II CRISPR-associated endonuclease Cas1- *Pastuerella multocida*	64%	DR	No	Proximal core gene is NEIS0385 (Zhang et al., 2011)
	NEIS2567	CRISPR protein Cas1	49.73	No	type II CRISPR RNA-guided endonuclease Cas -*Actinobacillus minor*	68%			
	CDS	CRISPR protein Cas9	48.72	Yes	CRISPR protein Cas9 - *Actinobacillus minor*	67%			
		IS1106					IR		
Island 16 Iron Acquisition	NEIS0387	TonB-dependent receptor	55.38	Yes	TonB-dependent receptor - *Morococcus cerebrosus*	84%	IR		Proximal core gene is NEIS0386
	NEIS0388	esterase	56.48	Yes	Esterase - *Morococcus cerebrosus*	55%			

Island 17 (Pgl-2) Metabolism	NEIS0398	haloacid dehalogenase	50.51	No	haloacid dehalogenase - *Pseudoalteromonas sp.*	45%	No	No	Proximal core gene is NEIS0397 in the pilin glycan biosynthesis locus (Kahler et al., 2001)
Island 18A (PglB1) Membrane	NEIS0399	PglB1	50.79	No	UDP-N-acetylgalactosaminyltransferase - *Pseudomonadaceae*	58%	No	No	Proximal core gene is NEIS0398 (Kahler et al., 2001)
Island 18B (PglB2) Membrane	NEIS2838	PglB2	50.79	No	UDP-N-acetylgalactosaminyltransferase - *Pseudomonadaceae*				
Island 19 (PglH) Membrane	NEIS0400	glycosyltransferase family 1 (PglH)	52	No	None		No	No	Proximal core gene is NEIS0399<br><br>(Power et al., 2003); (Hadjineophytou et al., 2019)
Island 20 (PglG) Membrane	NEIS0401	glycosyl transferase 4-like domain protein (PglG)	56.16	Yes	None		No	No	Proximal core gene is NEIS0400 (Power et al., 2003); (Hadjineophytou et al., 2019)
Island 21A (TpsA/B) Toxin	NEIS0443	Hemin transporter TpsB	45.96	Yes	None		IR	No	Proximal core gene is NEIS0441 (Arenas et al., 2013)
	CDS	hemagglutinin/hemolysin-related protein TpsA	48.64	Yes	pre-toxin domain with VENN motif family protein- *Morococcus cerebrosus*	96%			
Island 21B (CDI) Toxin	NEIS0453	Hypothetical protein	30.47	Yes	Hypothetical protein - *Enterobacteriaceae*	37%			
	NEIS0454	Hypothetical protein	32.23	Yes	Hypothetical protein - *Bacillus sp.*				
Island 21C (CDI)	CDS	hemagglutinin	35.19	Yes	Hemagglutinin - *Morococcus cerebrosus*				

Toxin	NEIS2490	Chain A, The Crystal Structure Of An Immunity Protein Nmb0503	25.69	Yes	Hypothetical protein - *Morococcus cerebrosus*	99%			
Island 21D (CDI) Toxin	CDS	hemagglutinin	47.86	Yes	None				
	NEIS2472	Chain A, The Crystal Structure Of Functionally Unknown Protein From Neisseria Meningitidis Mc58	28.1	Yes	hypothetical protein, partial - *Photorhabdus luminescens*	37%			
			0	Yes					
			0	Yes					
	NEIS2471	hypothetical protein NM96060_2231	30.13	Yes	hypothetical protein - *Enterobacter lignolyticus*	32%			
	CDS	pre-toxin domain with VENN motif family protein	48.8	Yes	None				
Island 21E (CDI) Toxin	NEIS0449	Hypothetical protein	31.82	Yes	MULTISPECIES: 3'-5' exoribonuclease - *Bacillus cereus group*	55%			
	CDS	pre-toxin domain with VENN motif family protein	54.55	Yes	None				
	NEIS0456	pre-toxin domain with VENN motif family protein	43.55	Yes	pre-toxin domain with VENN motif family protein - *Acinetobacter baumannii*	51%			
Island 22 (FrpD/C) Iron acquisition	NEIS0526	FrpC Operon Protein FrpD	38.97	Yes	None		DR	No	Proximal core gene is NEIS0524 (Prochazkova et al., 2005, Thompson et al., 1993)
	NEIS0527	Iron regulated FrpA/C	45.5	Yes	None		IR	No	
Island 23A (MGI-2) Toxin	NEIS0586	MafB1 MGI-2	49.21	Yes	None		IR		Proximal core gene is *ntpA* (NEIS0585) (Jamet et al., 2015);
	NEIS0587	Hypothetical protein	28.39	Yes	O-succinylbenzoic acid--CoA ligase -*Cyclobacteriaceae bacterium*	28%			
	NEIS0588	Putative lipoprotein	43.92	Yes	None				
	NEIS0589	MafI1_MGI-2 (PUBMLST)	25.69	Yes	Barnase inhibitor *Coromonas testosterone*	34%			

	NEIS0591	Hypothetical protein	31.9	Yes	None				
	NEIS0592	Putative immunity protein	25.93	Yes	Immunity protein – *Kingella negevensis*	93.75%			
	NEIS0593	HNH endonuclease	44.31	Yes<br>Yes	HNH endonuclease – *Acinetobacter baumanii*	70.13%			
	NEIS0594	Hypothetical protein	32.04	Yes	Hypothetical protein – *Morococcus cerobrosus*	65%			
	NEIS0595	Hypothetical protein	33.08	Yes	None				
	NEIS0596	MafA – MGI -2	59.13	Yes	None				
	NEIS0597	MafB – Found in both MGI-1 and MGI-2	52.4	No	None				
	NEIS0598	Hypothetical protein	33.96	Yes	Hypothetical protein – *Kingella potus*	58.33%			
	NEIS0601	MafS2 – MGI11 or MGI-2	44.99	No	None				
	NEIS0602	Hypothetical protein	35.19	Yes	None				
	NEIS0603	Hypothetical protein	39.87	Yes	None				
	NEIS0604	Suppressor of fused family protein (SUFU)	59.27	Yes	SUFU protein – *Leptospira licerasiae*	44%			
	NEIS0605	Suppressor of fused family protein (SUFU)	38.3	Yes	SUFU protein – *Kingella sp*				
	NEIS0606	Hypothetical protein	52.78	Yes	None				
	NEIS0607	Hypothetical protein	25.37	Yes	None				
	NEIS0608	Hypothetical protein	30.26	Yes	Nona				
		IS1016							
Island 23B (MGI-2) Toxin	NEIS0586	MafB1 MGI-2	49.21	Yes	None				Proximal core gene is *ntpA* (NEIS0585) (Jamet et al., 2015);
	NEIS0591	Hypothetical protein	31.44	Yes	None				
	NEIS0592	Putative immunity protein	25.93	Yes	Immunity protein – *Kingella negevensis*	93.75%			
	NEIS0593	HNH endonuclease	44.31	Yes<br>Yes	HNH endonuclease – *Acinetobacter baumanii*	70.13%			
	NEIS0594	Hypothetical protein	32.04	Yes	Hypothetical protein – *Morococcus cerobrosus*	65%			

	NEIS0595	Hypothetical protein	33.08	Yes	None				
	NEIS0596	MafA – MGI -2	59.13	Yes	None				
	NEIS0597	MafB – Found in both MGI-1 and MGI-2	52.4	No	None				
	NEIS0598	Hypothetical protein	33.96	Yes	Hypothetical protein – *Kingella potus*				
	NEIS2694	Hypothetical protein	35.57	Yes	None				
	NEIS1791	Hypothetical protein	32.51	Yes	Hypothetical protein - *Vibrio splendicus*	38%			
	NEIS1792	Hypothetical protein	34.62	Yes	Hypothetical protein - *Tolumonas sp.*	58%			
	NEIS1800	MafB- MGI-1	45.87	No	None				
	NEIS1795	Hypothetical protein	32.19	Yes					
		IS1016							
Island 24 Membrane	NEIS0632	lipoprotein	52.33<br>0	No	Hypothetical protein – *Dyella japonica*	44%	No	No	Proximal gene is NEIS0631
Island 25A (Vsr-1) Restriction modification	NEIS0678	HpaII very short patch repair endonuclease	45.86	Yes	*Aggregatibacter actinomycetemcomitans*	95%	No	No	Proximal core gene is *rplT* (NEIS0675) (Claus et al., 2000)
	CDS-1	putative modification methylase NmeDI	41.43	Yes	Modification methylase – *Morococcus cerebrosus*	99%			
	NEIS0680	type II restriction endonuclease NmeDIP	42.99	Yes	Restriction endonuclease NmeDIP - *Morococcus cerebrosus*	99%			
Island 25B Restriction modification	CDS-1	Unnamed protein product (top hit)	32.2	Yes	Restriction endonuclease subunit M - *Kingella kingae*	80%	No	No	As above
	CDS-2	Unnamed protein product (top hit)	27.74	Yes	Restriction endonuclease subunit M - *Kingella kingae*	80%			
Island 25C Restriction modification	NEIS2377	modification methylase HgaI-1,restriction endonuclease	33.62	Yes	Restriction endonuclease subunit M - *Alysiella crassa*	75%			As above
	NEIS2378	type II restriction enzyme HgaI	31.02	Yes	Restriction endonuclease – *Alysiella crassa*	79%			
	NEIS2379	N-6 adenine-specific DNA methylase	32.72	Yes	DNA methyltransferase - *Alysiella crassa*	85%			
Island 26	NEIS0771	DNA methyltransferase	33.58	Yes	DNA methyltransferase *Haemophilus influenzae*	88%			

Restriction modification	NEIS0772	type II restriction endonuclease	31.59	Yes	restriction endonuclease - *Paenibacillus sophorae*	82%			Proximal core gene is *hldA* (NEIS0769) (Shih et al., 2001)
Island 27A Hypothetical	NEIS0796	Hypothetical protein	26.28	Yes	None	100%			Proximal core gene is NEIS0795
Island 27B Hypothetical	NEIS2392	Hypothetical protein	38.1	Yes	Hypothetical protein - *Chryseobacterium gallinarum*	62%			See above
	NEIS0798	Hypothetical protein	42.49	Yes	Hypothetical protein - *Moraxella caprae*	47%			
	NEIS0800	Hypothetical protein	35.52	Yes	hypothetical protein - *Alysiella crassa*	41%			
	NEIS0801	Membrane protein	46.99	Yes	RHS repeat-associated core domain protein -*Alcanivorax pacificus*	63%			
Island 28 (PilX) Membrane	NEIS0831	type IV pilin PilX	42.74	Yes	None	100%			Proximal core gene is *pilK* (NEIS0830) (Helaine et al., 2005)
	NEIS0832	branched-chain amino acid ABC transporter permease	50.35	No	branched-chain amino acid ABC transporter permease - *Morococcus cerbrosus*	83%			
Island 29 Phage	NEIS0836	Phage integrase	55.89	Yes	Hypothetical protein - *Eikenella sp.*	39%	IR	Yes (2 tRNAs before integrase)	Proximal core gene is NEIS0835<br><br>This locus is not the core locus *ldhA* described by Sigurlasdottir et al. (Sigurlasdottir et al., 2019)
	NEIS0837	mRNA interferase PemK	49.87	No	Hypothetical protein - *Haemophilus parasuis*	64%			
	NEIS0838	Hypothetical protein	58.2	Yes	ABC transporter permease - *Bifidobacterium cuniculi*	39%			
	NEIS0839	DUF4760 domain protein	47.62	Yes	DUF4760 domain protein - *Moraxella caprae*	62%			
	NEIS0840	DNA binding protein	52.94	No	Hypothetical protein - *Eikenella sp.*	60%			
	NEIS0841	Lactate dehydrogenase	54.39	Yes	D-isomer specific 2-hydroxyacid dehydrogenase - *Amycolatopsis decaplanina*	43%			
	NEIS0842	Hypothetical protein	46.88	Yes	Hypothetical protein - *Eikenella corrodens*	63%			

	NEIS0843	Hypothetical protein	50.34	No	Hyothetical protein - *Nonomuraea coxensis*	33% (e-value of 3)			
	NEIS0844	Hypothetical protein	55.56	Yes	Dystonin like protein - *Pygoscelis adeliae*	30%			
	NEIS0845	Hypothetical protein	53.7	No	LSM domain containing protein - *Cryptosporidium muris*	45%			
	NEIS0846	Hypothetical protein	47.02	Yes	Hypothetical protein - *Microvirgula aerodenitrificans*	44%			
	NEIS0847	Hypothetical protein	47.22	Yes	Methyltransferase small domain protein -*Snodgrassella alvi*	59%			
	NEIS0848	Hypothetical protein	42.79	Yes	Hypothetical protein - *Haemophilus ducreyi*				
	NEIS0849	Hypothetical protein	45.9	Yes	Hypothetical protein -*Kingella deritrificans*	38%			
	NEIS0850	Transcriptional regulator	54.25	No	Repressor - *Kingella oralis*	55%			
	NEIS0852	Hypothetical protein	50.86	No	Thiol reductase thioredoxin - *Porphyromonas*	*35%*			
	NEIS0853	Primase	48.3	Yes	Hypothetical protein - *Simonsiella muelleri*	43%			
	NEIS0854	Hypothetical protein	45.71	Yes	Al hits are for Neisseria	100%			
	NEIS0855	Hypothetical protein	48.32	Yes	Hypotherical protein - *Pseudomonas nitroducens*	68%			
	NEIS0856	Hypothetical protein	53.73	No	Hypothetical protein - *Chitinilyticum aquatile*	45%			
	NEIS0857	Phage portal protein	57.01	Yes	Hypothetical protein - *Pseudohodoferax sp.*	54%			
	NEIS0858	Peptidase	53.88	No	Capsid protein - *Thioalkalivibrio sp.*	45%			
	NEIS0859	Hypothetical protein	54.39	Yes	Hypothetical protein - *Fischerella sp.*	30%			
	NEIS0860	Hypothetical protein	51.16	No	Hypothetical protein - *Nitrafractor salsuginis*	34%			
	NEIS0861	Hypothetical protein	52.91	No	alpha -2 - macroglobulin - *Shewanella algae*	33%			
	NEIS0862	Phage tail protein	48.75	Yes	Phage tail protein - *Alysiella crassa*	49%			
	NEIS0863	Hypothetical protein	49.68	No	Hypothetical protein - *Acromobacter sp.*	48%			
	NEIS0864	Hypothetical protein	49.82	No	Phage protein - *Pseudomonas aeruginosa*	43%			

	NEIS0865	Phage tail protein	53.18	No	Hypothetical protein - *Conchiformibius kuhniae*	40%			
	NEIS0866	Phage minor tail family protein	50	No	Minor tail family protein - *Pseudomonas protegens*	43%			
	NEIS0867	tail protein	56.43	Yes	Tail protein - *Simonsiella muelleri*	64%			
	NEIS0868	alkaline phosphatase	55.56	Yes	Alkaline phosphatase - *Vitreoscilla sp.*	54%			
	NEIS0869	Phage tail assembly protein	55.74	Yes	Phage tail assembly protein - *Conchiformibius kuhniae*	55%			
	NEIS0870	Hypothetical protein	51.83	No	Hypothetical protein - *Acinetbacter gyllenbergii*	24%			
	NEIS0871	Hypothetical protein	44.97	Yes	Hypothetical protein - *Plesiocystic pacifica*	28%			
	NEIS0872	membrane protein	48.31	Yes	Hypothetical protein - *Kingella kingae*	40%			
	NEIS0873	Hypothetical protein	51.82	No	Hypothetical protein - *Conchiformibius kuhniae*	35%			
	NEIS0877	ATP-Binding protein	55.7	Yes	ATP-Binding protein - *Moraxella bovoculi*	59%			
	NEIS0878	Phage associated membrane protein	52.55	No	Hypothetical protein - *Kingella oralis*	34%			
Island 30 Membrane		IS5	51.53	No	DDE transposase - *Eikenella corrodens*	98%	IR	No	Proximal core gene is NEIS0422
	CDS-1	Hypothetical protein	53.05	No	None				
	NEIS0887	Hemolysin D family secretion protein	36.9	Yes	Transporter - *Acinetobacter nosocomialis*	44%			
	NEIS0888	ABC transporter	40.3	Yes	ABC transporter - *Acinetobacter baumannii*	54%			
	NEIS0889	membrane protein	44.64	Yes	Hypothetical protein - *Oceanospirillum maris*	35%			
	NEIS0890	Membrane protein	19.76	Yes	Organic cation transporter like protein-F150 *Papilio polytes*	29%			
	NEIS0891	Cupin family protein	32.03	Yes	Cupin - *Simonsiella muelleri*	58%			
	NEIS0892	Hypothetical protein	27.35	Yes	None				
	NEIS0893	ParB (chromosome partitioning protein)	35.29	Yes	ParB protein - *Haemophilus influenzae*	69%			
		IS5	53.46	No	DDE transposase - *Helicobacter pylori*	96%			

Island 31A Metabolism	CDS-1	Transcriptional regulator	51.59	No	WYL domain containing protein - *Gammaproteobacteria*	44%	No	No	Proximal core gene is NEIS0938
Island 31B Cell wall synthesis		IS200							See above
	NEIS0940	ATP-dependent helicase	28.42	Yes	DNA helicase - *Rhodivulum sulfidophilum*	44%			
Island 31C Hypothetical	NEIS2397	FunZ family protein	28.38	Yes	FunZ protein - *Klebsiella oxytoca*	59%	No	No	See above
							IR	No	Proximal core gene is NEIS0986
Island 32A (P2-like) Phage	NEIS2552	Putative phage associated protein	57.28	Yes	Putative phage associated protein - *Morococcus cerebrosus*	69%			
	NEIS0989	Putative phage integrase	54.35	Yes	Putative phage integrase - *Morococcus cerebrosus*	69%			
	NEIS0992	Putative phage integrase	62.33	Yes	Putative phage integrase	37%			
	NEIS0993	Phage protein	54.41	Yes	Hypothetical protein - *Kingella kingae*	70%			
	NEIS0995	DNA phage binding protein	53.69	No	DNA phage binding protein - *Alysiella crassa*	71%			
	NEIS0996	BcepMu gp16 family phage associated protein	51.92	No	Hypothetical protein - *Simonsiella muelleri*	78%			
	NESI0997	Phage repressor	41.27	Yes	Transcriptional regulator - *Actinobacillus capsulatus*	45%			
	NEIS0998	Hypothetical protein	41.46	Yes	SAM-dependent methyltransferase - *Steptococcus castoreus*	25%			
	NEIS0999	Hypothetical protein	44.09	Yes	None	100%			
	NEIS1000	Hypothetical protein	37.94	Yes	Hypothetical protein - *Peptoclostridium difficile*	98%			
	NEIS1001	Membrane protein	32.51	Yes	Hypothetical protein - *Haemophilus sp.*	47%			
	NEIS1002	Hypothetical protein	56.75	Yes	Peptidoglycan domain protein - *Morococcus cerebrosus*	92%			
Island 32B		Bacteriophage transposase					IR	Yes	
	CDS-1	Phage protein - Integrase NMO_0917	57.37	Yes	Integrase - *Pseudomonas sp.*	45%			See above
	CDS-2	putative phage associated conserved protein NMO_0918	56.62	Yes	Hypothetical protein - *Burkholderia cenocepacia*	42%			

	CDS-3	putative phage associated membrane protein NMO_0919	43.8	Yes	None				
	CDS-4	Rha family transcriptional regulator NMO_0920	57.65	Yes	Hypothetical protein - *Eikenella corrodens*	66%			
	CDS-5	putative bacteriophage transcriptional regulator NMO_0921	54.68	Yes	DNA binding protein - *Morococcus cerebrosus*	89%			
	CDS-6	ATPases with chaperone activity ATP-binding subunit NMO_0922	57.27	Yes	2-oxoacid:acceptor oxoreductase - *Alysiella crassa*	54%			
	CDS-7	Isocitrate/isopropylmalate dehydrogenase NMO_0923	56.83	Yes	Hypothetical protein *Morococcus cerebrosus*	95%			
	CDS-8	putative phage associated hypothetical protein NMO_0924	51.11	No	Hypothetical protein *Kingella oralis*	45%			
	CDS-9	putative phage associated hypothetical protein NMO_0925	57.82	Yes	Hypothetical protein *Eikenella corrodens*	68%			
	CDS-10	putative phage associated hypothetical protein NMO_0926	55.56	Yes	Hypothetical protein *Morococcus cerebrosus*	91%			
	CDS-11	putative phage associated protein NMO_0927	58.45	Yes	Hypothetical protein *Kingella kingae*	72%			
	CDS-12	putative phage associated protein NMO_0928	55.02	Yes	Hypothetical protein *Kingella kingae*	68%			
	CDS-13	putative phage major tail sheath protein NMO_0929	56.77	Yes	phage tail protein *Morococcus cerebrosus*	88%			
	CDS-14	Phage tail tube protein FII NMO_0930	50.78	No	tail protein *Morococcus cerebrosus*	95%			
	CDS-15	putative phage associated hypothetical protein NMO_0931	52.19	No	Hypothetical protein *Kingella kingae*	56%			
	CDS-16	Phage-related tail protein NMO_0932	54.86	Yes	Phage tail tape measure protein *Moraxella caprae*	43%			

	CDS-17	putative phage tail assembly protein NMO_0933	57.4	Yes	tail protein *Morococcus cerebrosus*	88%			
	CDS-18	putative phage tail protein NMO_0934	54.34	Yes	membrane protein *Kingella kingae*	80%			
	CDS-19	putative bacteriophage regulatory protein NMO_0935	53.24	No	Hypothetical protein *Morococcus cerebrosus*	88%			
	CDS-20	Phage P2 baseplate assembly protein gpV NMO_0936	56.76	Yes	Phage baseplate protein *Morococcus cerebrosus*	87%			
	CDS-21	Phage baseplate assembly protein W NMO_0937	54.5	Yes	Phage baseplate protein *Morococcus cerebrosus*	92%			
	CDS-22	putative phage-related baseplate assembly protein NMO_0938	57.58	Yes	Baseplate assembly protein *Alysiella crassa*	63%			
	CDS-23	Bacteriophage P2-related tail formation protein NMO_0939	57.17	Yes	Tail fiber protein - *Morococcus cerebrosus*	79%			
	CDS-24	putative phage tail fibre protein NMO_0940	59.53	Yes	None				
	CDS-25	putative phage fibre-spike protein NMO_0941	56.22	Yes	Hypothetical protein - *Kingella kingae*	50%			
	CDS-26	putative phage associated hypothetical protein NMO_0942	42.96	Yes	None				
		putative phage associated type II DNA-methyltransferase NMO_0943	50.64	No	DNA methyltransferase - *Morococcus cerebrosus*	95%			
		Bacteriophage transposase							
Island 33A (IHT-E)(PNM2) Phage	NEIS1041	ABC transporter, ATP binding	58.74	Yes	ABC transporter permease – *Moroccocus cerebrosus*	92%	IR/DR	No	(Masignani et al., 2001)
	NEIS1042	Hypothetical protein	46.67	Yes	None	100%			
	NEIS1044	Transcriptional regulator/DNA binding protein	38.89	Yes	Putative DNA binding protein (fragment) - *Peptoclostridium difficile*	99%			

	NEIS1045	transcriptional regulator/DNA binding protein	38.49	Yes	XRE family transcriptional regulator - *Alysiella crassa*	60%			Proximal core gene is NEIS1040<br><br>(Joseph et al., 2011)
	NEIS1046	Phage transposase	54.34	Yes	Putative phage transposase - *Snodgrasella alvi*	51%			
	NEIS1048	Phage transposase	50.82	No	XRE family transcriptional regulator - *Citrobacter freundii*	45%			
	NEIS1049	Hypothetical protein	48.45	Yes	None				
	NEIS1050	Hypothetical protein	44.44	Yes	Hypothetical protein - *Peptoclostridium difficile*	97%			
	NEIS1051	Hypothetical protein	52.87	No	None				
	NEIS1052	Hypothetical protein	52.38	No	None				
	NEIS1053	Hypothetical protein	35.53	Yes	None				
	NEIS1054	Host nuclease inhibitor (gam)	52.22	No	Host nuclease inhibitor (gam) - *Kingella oralis*	51%			
	NEIS1055	transposase	51.35	No	None				
	NEIS1057	Phage tail protein	61.68	Yes	Phage tail protein - *Kingella kingae*	59%			
	NEIS1058	tail protein	58.21	Yes	Phage tail fibre protein - *Haemophilus aegypticus*	59%			
	NEIS1059	Hypothetical protein	59.24	Yes	Hypothetical protein - *Haemophilus haemolyticus*	59%			
	NEIS1060	DNA helicase UrvD	58.92	Yes	DNA helicase UrvD - *Haemophilus influenzae*	74%			
	NEIS1062	ABC transporter, ATP binding	52.27	No	ABC transporter, ATP binding - *Kingella oralis*	77%			
		Transposase					IR/DR	No	
Island 33B (MuMenB)	NEIS2412	Hypothetical protein	35.53	Yes	Hypothetical protein - *Kingella kingae*	42%			(Masignani et al., 2001)
	NEIS2413	Phage protein	56.28	Yes	Phage protein - *Avibacterium paragallinarum*	71%			
	NEIS2414	Mu bacteriophage protein gp29	56.21	Yes	Mu bacteriophage protein gp29 - *Kingella kingae*	64%			
	NEIS2415	Phage head morphogenesis protein	48.84	Yes	Phage head morphogenesis protein - *Kingella kingae*	66%			
	NESI2416	Bacteriophage Mu I protein GP32	54.14	No	Hypothetical protein - *Kingella kingae*	74%			
	NEIS2417	Hypothetical protein	57.76	Yes	Hypothetical protein - *Kingella kingae*	56%			

	NEIS2418	Hypothetical protein	58.39	Yes	Hypothetical protein - *Kingella kingae*	72%			
	NESI2419	Mu-like prophage FlmU protein gp37	57.41	Yes	Hypothetical protein - *Kingella kingae*	64%			
	NEIS2420	Hypothetical protein	54.55	Yes	Hypothetical protein - *Kingella kingae*	62%			
	NEIS2421	tail sheath protein	57.09	Yes	Tail sheath protein - *Kingella kingae*	73%			
	NEIS2422	Phage tail protein	51.85	No	Phage tail protein - *Kingella kingae*	79%			
	NEIS2423	Hypothetical protein	59.29	Yes	Hypothetical protein - *Kingella kingae*	51%			
	NEIS2424	lipoprotein	38.81	Yes	Transporter - *Photobacterium ganghwense*	25%			
	NEIS2425	PRTRC system protein F	53.13	No	Hypothetical protein - *Kingella oralis*	41%			
	NEIS2426	Phage morphogenesis protein	58.39	Yes	Phage morohogenesis protein - *Kingella kingae*	54%			
	NEIS2427	Phage tail protein	57.85	Yes	Tail protein - *Kingella kingaae*	72%			
	NEIS2428	Baseplate assembly protein	56.35	Yes	Baseplate assembly protein - *Haemophilus parasuis*	58%			
	NEIS2429	Hypothetical protein	58.62	Yes	Hypothetical proten - *Kingella kingae*	70%			
	NEIS2430	Hypothetical protein	52.48	No	Hypothetical protein - *Haemophilus influenzae*	59%			
	NEIS2431	Hypothetical protein	45.14	Yes	Hypothetical protein - *Gallibacterium anatis*	68%			
Island 34 Membrane	NEIS1074	Amino acid permease	55.78	Yes	Hypothetical protein - *Morococcus cerbrosus*	36%	No	No	Proximal core gene is NEIS1073
Island 35 Membrane	NEIS1082	lipoprotein	42.62	Yes	hypothetical protein - *Marinobacterium rhizophilum*	36%	No	No	Proximal core gene is NEIS1081
	NEIS1083	Hypothetical protein	36.72	Yes	Hypothetical protein - *Pasteurella multocida*	40%			
Island 36 (HlyC) Toxin	NEIS2434	hemolysin-activating lysine-acyltransferase HlyC	47.01	Yes	HlyC - *Acinetobacter guillouiae*	46%	IR	No	Proximal core gene is NEIS1108
	NEIS2435	Hypothetical protein	38.27	Yes	Hypothetical protein - *Cardiobacterium hominis*	33%			

	NEIS2436	Lipoprotein	47.2	Yes	Hypothetical protein - *Haemophilus influenza*	41%			
	NEIS2437	Lipoprotein	44.9	Yes	Hypothetical protein - *Haemophilus influenza*	36%			
	NEIS2438	Hemagglutinin	50.6	No	Hemagglutinin –*Haemophilus haemolyticus*	40%			
Island 37A Metabolism	NEIS1117	Beta-lactamase fold protein	50.37	No	Hypothetical protein - *Eikenella corrodens*	87%	IR	No	Proximal core gene is NEIS1116 which is the site for the T4SS islands in lineage 5 (Harrison et al., 2015)
	NEIS1118	MarR family transcriptional regulator	52.44	No	MarR family transcriptional regulator - *Eikenella corrodens*	96%			
	CDS-1	prokaryotic cytochrome b561 family protein	52.44	No	cytochrome B561 - *Cardiobacterium valvarum*	62%			
	NEIS1121	NADH dehydrogenase	51.32	No	cytochrome B561 - *Cardiobacterium valvarum*	55%			
	NEIS1122	antibiotic biosynthesis monooxygenase	53.55	No	polysaccharide biosynthesis protein - *Phaeobacter gallaeciensis*	60%			
	CDS-2	Hypothetical protein	53.01	No	DNA binding response regulator	27%			
		IS630							
Island 37B Restriction modification	CDS-1	restriction endonuclease subunit M	51.39	No	type I restriction-modification protein subunit M - *M.catarrhalis*	87%	No	No	See above
	CDS-2	N-6 DNA Methylase family protein	50.21	No	type I restriction-modification protein subunit M - *M.catarrhalis*	74%			
	CDS-3	type I restriction-modification system DNA-methyltransferase	43.55	Yes	Hypothetical protein - *Gallibacterium anatis*	77%			
	CDS-4	myosin VC domain protein	50	No	type I restriction-modification protein subunit M - *M.catarrhalis*	51%			
Island 38 Restriction modification	NEIS1158	type I restriction enzyme system modification protein"	41.28	Yes	DNA methyltransferase *Cardiobacterium hominis*	89%	No	No	Proximal core gene is NEIS1157
Island 39 (ModB) Restriction modification	NEIS1193	type III restriction-modification system endonuclease protein"	48.37	Yes	type III restriction-modification system endonuclease - *Mannheimia succiniciproducens*	73%	DR	No	Proximal core gene is NEIS1195 (*mip*) (Srikhanta et al., 2009)
	NEIS1194	Type III restriction/modification system methyltransferase (ModB)	45.11	Yes	type III restriction endonuclease subunit M -*Haemophilus influenza*	66%			

Island 40 Metabolism	NEIS1225	CAAX amino protease	41.45	Yes	CAAX amino protease - *Morococcus cerebrosus*	94%	No	No	Proximal core gene is NEIS1223
Island 41A Toxin	NEIS1357	addiction module protein	50.62	No	Addicton module protein - *Alysiella crassa*	77%	IR	No	Proximal core gene is *fis* (NEIS1353) (Lehnherr et al., 1993)
Island 41B Metabolism	NEIS1358	peptidase	53.47	No	Hypothetical protein - *Moraxella boevrei*	32%			
Island 42 Hypothetical	NEIS1424	hypothetical protein	47.58	Yes	Hypothetical protein - *Kingella sp.*	59%	No	No	Proximal core gene is NEIS1423
Island 43 Restriction modification	NEIS1519	Type II restriction nuclease	44.57	Yes	Type II restriction endonuclease - *Methylococcus capsulatus*	58%	IR	No	Proximal core gene is NEIS1518
	NEIS1520	C-5 cytosine-specific DNA methylase	42.89	Yes	DNA methyltransferase - *Methylococcus capsulatus*	70%			
Island 44 GpxA Metabolism	NEIS1547	glutathione peroxidase GpxA	48.5	Yes	Glutathione peroxidase - *Kingella sp.*	71%	No	No	Proximal core gene is NEIS1546 (Moore and Sparling, 1995)
Island 45 Toxin	NEIS1583	excinuclease ATPase subunit	52.79	No	Excinuclease - *Conchiformibius steedae*	55%	No	No	Proximal core gene is NEIS1582<br><br>(Liu et al., 2008)
	NEIS1584	Death on curing protein, Doc toxin, partial	42.64	Yes	DNA-binding protein - *Serratia marcescens*	60%	IR	No	
	NEIS1585	prevent-host-death protein	39.36	Yes	Prevent-host-death protein - *Serratia marcescens*	52%			
Island 46 Hypothetical	NEIS1626	Hypothetical protein	31.71	Yes	Hypothetical protein - *Streptococcus sp.*	99%	No	No	Proximal core gene is NEIS1625
Island 47 (IHT-C) Toxin	NEIS2495	Virulence associated protein VapD (CRISPR associated protein)	39.95	Yes	VapD - *Kingella dentrificans*	91%	IR	No	Proximal gene is *tspB* (NEIS1715)
	NEIS2496	Hypothetical protein	39.13	Yes	acetyl-CoA carboxylase carboxyl transferase subunit alpha – *Methylibium sp*	34%			
	NEIS2497	Hypothetical protein	40	Yes	None				
	NEIS2470	Hypothetical protein	34.74	Yes	None				

	NEIS2498	Fic family protein	43.96	Yes	Fic family protein - *Actinobacillus sp*	64%			(Tettelin et al., 2000)
	NEIS2499	Hypothetical protein	40.83	Yes	Hypothetical protein - *Actinobacillus ureae*	83%			
	NEIS2500	HipA family protein	42.34	Yes	Phosphatidylinositol kinase - *Polarmonas sp*	33%			
	NEIS2501	hemin transporter	47.99	Yes	Hemin transporter - *Kingella sp.*	76%			
	NEIS2502	HlyC protein (RTX toxin)	45.32	Yes	Hypothetical protein - *Acinetobacter sp.*	46%			
	NEIS2503	lipoprotein	37.14	Yes	Hypothetical protein - *Histophilus somni*	50%			
	NEIS2504	Hypothetical protein	31.67	Yes	Hypothetical protein - *Neisseria flavescens*	28%			
	NEIS2505	Hemagglutinin	37.57	Yes	Hemagglutinin - *N. lactamica*	82%			
	CDS-1	Hemagglutinin	46.19	Yes	Hemagglutinin - *N. lactamica*	92%			
Island 48 Hypothetical	NEIS1695	hypothetical protein	44.65	Yes	Cell division protein - *Campylobacter fetus*	43%	No	No	Proximal core gene is NEIS1694
Island 49A VanX Cell wall synthesis	NEIS1730	Putative integral membrane protein	37.68	Yes	Membrane protein - *Xenorhabdus bovienii*	63%	No	No	Proximal core gene NEIS1729 (Deghorain et al., 2007, Joseph et al., 2010)
	NEIS1731	ddpX D-alanyl-D-alanine dipeptidase (VanX)	42.01	Yes	Putative D-alanyl-D-alanine dipeptidase - *Xenorhabdus cabanillasii*	46%			
Island 49B Membrane	NEIS2488	YfcA inner membrane protein	49.68	No	Membrane protein - *Acinetobacter calcoaceticus*	70%	No	No	As above
Island 50 Hypothetical	NEIS1801	hypothetical protein	33.95	Yes	hypothetical protein - *Kiloniella laminariae*	27%	No	No	Proximal region is Island 23 preceded by NEIS1788
Island 51 Membrane	NEIS1802	lipoprotein	26.11	Yes	hypothetical protein - *Aneurinibacillus migulanus*	38%	No	No	Proximal region is NEIS1801 (Island 50)
Island 52 Hypothetical	NEIS1803	hypothetical protein	38.69	Yes	hypothetical protein - *Rothia dentocariosa*	41%	No	No	Proximal region is NEIS1802 (Island 51)

Island 53 Toxin	NEIS2370	Immunity protein	31.95	Yes	None		No	No	Proximal core gene is NEIS1853
Island 54 Metabolism	NEIS1886	Helicase	39.55	Yes	Helicase - *Methylomicrobium agile*	61%	No	No	Proximal core gene is NEIS1885
Island 55 Hypothetical	NEIS1940	Hypothetical protein	25.64	Yes	hypothetical protein - *Ralstonia solanacearum*	55%	No	No	Proximal core gene is NEIS1939
	NEIS1941	Hypothetical protein	40.03	Yes	hypothetical protein, partial - *Cardiobacterium hominis*	61%			
Island 56 (HpuA/B) Iron acquisition	NEIS1946 (*hpuA*)	hemoglobin receptor component HpuA	52.53	No	None		IR/DR	No	Proximal core gene is NEIS1945 (Lewis et al., 1997)
	NEIS1947 (*hpuB*)	hemoglobin-haptoglobin utilization protein HpuB	55.03	Yes	hemoglobin-haptoglobin utilization protein - *Morococcus cerebrosus*	78%			
Island 57 (MspA) Membrane	CDS	immunoglobulin A1 protease	52.87	No	None		DR	No	Proximal core gene is NEIS1973
	NEIS1974	MspA	56.94	Yes	Hypothetical protein - *Helicobacter pylori*	99%			(Turner et al., 2006)
Island 58 (Vsr-2) Restriction modification	NEIS1990	patch repair protein (Vsr)	37.73	Yes	Very short patch repair protein - *Actinobacillus suis*	66%	No	No	Proximal core gene is NEIS1987 (Bazlekowa et al., 2017)
	NEIS1991	GHKL domain containing protein	36.9	Yes	Molecular chaperone - *Chlenobacter oris*	57%			
	NEIS1992	cytosine-specific DNA methylase	40.72	Yes	Modification methylase - *Haemophilus influenzae*	79%			
Island 59 (LgtG) Membrane	NEIS2011	glycosyltransfersase family 1 (LgtG)	56.37	Yes	None		No	No	Proximal core gene is NEIS2010 (Banerjee et al., 1998) (Kahler et al., 2005)
Island 60 (Lpt6) Membrane	NEIS2012	lipopolysaccharide inner-core distal heptose 6-phosphoethanolamine transferase (Lpt6)	53.96	No	Membrane protein - *Haemophilus influenzae*	69%	No	No	See above (Wright et al., 2004, Kahler et al., 2005)
Island 61 Restriction modification	(Preceded by NEIS2062) CDS-1	Restriction endonuclease	42.03	Yes	Modification methylase - *Haemophilus influenzae*	77%	No	No	Proximal core gene is NEIS2061

	CDS -2	Restriction endonuclease	37.74	Yes	Restriction endonuclease - *Haemophilus influenzae*	67%			
Island 62 (MGI-3 MafI) Toxin	NEIS2085	MafI immunity protein	25.47	Yes	Hypothetical protein - *Eikenella corrodens*	45%	IR/DR	No	Proximal gene for this *maf* locus is 2082<br><br>(Jamet et al., 2015)
Island 63 (modD)	NEIS2364	Type II Restriction Modification Enzyme	49.7	No	DNA methyltransferase – *Bergeriella denitfificans*	69.88%	No	No	(Seib et al., 2011)

REFERENCES for Supplementary Table 2

ALLUNANS, J., KRISTIANSEN, K. I., ASSALKHOU, R. & BJORAS, M. 2008. Bacteriocins (meningocins) in Norwegian isolates of Neisseria meningitidis: possible role in the course of a meningococcal epidemic. *APMIS,* 116**,** 333-44.

ARENAS, J., SCHIPPER, K., VAN ULSEN, P., VAN DER ENDE, A. & TOMMASSEN, J. 2013. Domain exchange at the 3' end of the gene encoding the fratricide meningococcal two-partner secretion protein A. *BMC Genomics,* 14**,** 622.

BANERJEE, A., WANG, R., ULJON, S. N., RICE, P. A., GOTSCHLICH, E. C. & STEIN, D. C. 1998. Identification of the gene (lgtG) encoding the lipooligosaccharide beta chain synthesizing glucosyl transferase from Neisseria gonorrhoeae. *Proc Natl Acad Sci U S A,* 95**,** 10872-7.

BASMAJI, F., MARTIN-YKEN, H., DURAND, F., DAGKESSAMANSKAIA, A., PICHEREAUX, C., ROSSIGNOL, M. & FRANCOIS, J. 2006. The 'interactome' of the Knr4/Smi1, a protein implicated in coordinating cell wall synthesis with bud emergence in Saccharomyces cerevisiae. *Mol Genet Genomics,* 275**,** 217-30.

BAZLEKOWA, M., ADAMCZYK-POPLAWSKA, M. & KWIATEK, A. 2017. Characterization of Vsr endonucleases from Neisseria meningitidis. *Microbiology (Reading),* 163**,** 1003-1015.

BILLE, E., URE, R., GRAY, S. J., KACZMARSKI, E. B., MCCARTHY, N. D., NASSIF, X., MAIDEN, M. C. & TINSLEY, C. R. 2008. Association of a bacteriophage with meningococcal disease in young adults. *PLoS One,* 3**,** e3885.

CLAUS, H., FRIEDRICH, A., FROSCH, M. & VOGEL, U. 2000. Differential distribution of novel restriction-modification systems in clonal lineages ofNeisseria meningitidis. *Journal of Bacteriology,* 182**,** 1296-1303.

DEGHORAIN, M., GOFFIN, P., FONTAINE, L., MAINARDI, J. L., DANIEL, R., ERRINGTON, J., HALLET, B. & HOLS, P. 2007. Selectivity for D-lactate incorporation into the peptidoglycan precursors of Lactobacillus plantarum: role of Aad, a VanX-like D-alanyl-D-alanine dipeptidase. *J Bacteriol,* 189**,** 4332-7.

DINH, T., PAULSEN, I. T. & SAIER, M. 1994. A family of extracytoplasmic proteins that allow transport of large molecules across the outer membranes of gram-negative bacteria. *Journal of Bacteriology,* 176**,** 3825-3831.

HADJINEOPHYTOU, C., ANONSEN, J. H., WANG, N., MA, K. C., VIBURIENE, R., VIK, A., HARRISON, O. B., MAIDEN, M. C. J., GRAD, Y. H. & KOOMEY, M. 2019. Genetic determinants of genus-level glycan diversity in a bacterial protein glycosylation system. *PLoS Genet,* 15**,** e1008532.

HARRISON, O. B., BRAY, J. E., MAIDEN, M. C. & CAUGANT, D. A. 2015. Genomic Analysis of the Evolution and Global Spread of Hyper-invasive Meningococcal Lineage 5. *EBioMedicine,* 2**,** 234-243.

HELAINE, S., CARBONNELLE, E., PROUVENSIER, L., BERETTI, J. L., NASSIF, X. & PELICIC, V. 2005. PilX, a pilus-associated protein essential for bacterial aggregation, is a key to pilus-facilitated attachment of *Neisseria meningitidis* to human cells. *Mol Microbiol,* 55**,** 65-77.

JAMET, A., JOUSSET, A. B., EUPHRASIE, D., MUKORAKO, P., BOUCHARLAT, A., DUCOUSSO, A., CHARBIT, A. & NASSIF, X. 2015. A new family of secreted toxins in pathogenic *Neisseria* species. *PLoS Pathog,* 11**,** e1004592.

JOLLEY, K. A., SUN, L., MOXON, E. R. & MAIDEN, M. C. 2004. Dam inactivation in Neisseria meningitidis: prevalence among diverse hyperinvasive lineages. *BMC microbiology,* 4**,** 1-7.

JOSEPH, B., SCHNEIKER-BEKEL, S., SCHRAMM-GLÜCK, A., BLOM, J., CLAUS, H., LINKE, B., SCHWARZ, R. F., BECKER, A., GOESMANN, A. & FROSCH, M. 2010. Comparative genome biology of a serogroup B carriage and disease strain supports a polygenic nature of meningococcal virulence. *Journal of bacteriology,* 192**,** 5363-5377.

JOSEPH, B., SCHWARZ, R. F., LINKE, B., BLOM, J., BECKER, A., CLAUS, H., GOESMANN, A., FROSCH, M., MULLER, T., VOGEL, U. & SCHOEN, C. 2011. Virulence evolution of the human pathogen Neisseria meningitidis by recombination in the core and accessory genome. *PLoS One,* 6**,** e18441.

KAHLER, C. M., DATTA, A., TZENG, Y. L., CARLSON, R. W. & STEPHENS, D. S. 2005. Inner core assembly and structure of the lipooligosaccharide of Neisseria meningitidis: capacity of strain NMB to express all known immunotype epitopes. *Glycobiology,* 15**,** 409-19.

KAHLER, C. M., MARTIN, L. E., TZENG, Y.-L., MILLER, Y. K., SHARKEY, K., STEPHENS, D. S. & DAVIES, J. K. 2001. Polymorphisms in pilin glycosylation locus of *Neisseria meningitidis* expressing class II pili. *Infection and immunity,* 69**,** 3597-3604.

KJOS, M., SNIPEN, L., SALEHIAN, Z., NES, I. F. & DIEP, D. B. 2010. The abi proteins and their involvement in bacteriocin self-immunity. *J Bacteriol,* 192**,** 2068-76.

LEHNHERR, H., MAGUIN, E., JAFRI, S. & YARMOLINSKY, M. B. 1993. Plasmid addiction genes of bacteriophage P1: doc, which causes cell death on curing of prophage, and phd, which prevents host death when prophage is retained. *J Mol Biol,* 233**,** 414-28.

LEWIS, L. A., GRAY, E., WANG, Y. P., ROE, B. A. & DYER, D. W. 1997. Molecular characterization of hpuAB, the haemoglobin–haptoglobin-utilization operon of *Neisseria meningitidis*. *Molecular microbiology,* 23**,** 737-749.

LIU, M., ZHANG, Y., INOUYE, M. & WOYCHIK, N. A. 2008. Bacterial addiction module toxin Doc inhibits translation elongation through its association with the 30S ribosomal subunit. *Proc Natl Acad Sci U S A,* 105**,** 5885-90.

MASIGNANI, V., GIULIANI, M. M., TETTELIN, H., COMANDUCCI, M., RAPPUOLI, R. & SCARLATO, V. 2001. Mu-like prophage in serogroup B Neisseria meningitidis coding for surface-exposed antigens. *Infection and immunity,* 69**,** 2580-2588.

MOORE, T. & SPARLING, P. F. 1995. Isolation and identification of a glutathione peroxidase homolog gene, gpxA, present in *Neisseria meningitidis* but absent in *Neisseria gonorrhoeae*. *Infection and immunity,* 63**,** 1603-1607.

POWER, P. M., RODDAM, L. F., RUTTER, K., FITZPATRICK, S. Z., SRIKHANTA, Y. N. & JENNINGS, M. P. 2003. Genetic characterization of pilin glycosylation and phase variation in *Neisseria meningitidis*. *Mol Microbiol,* 49**,** 833-47.

PROCHAZKOVA, K., OSICKA, R., LINHARTOVA, I., HALADA, P., SULC, M. & SEBO, P. 2005. The Neisseria meningitidis outer membrane lipoprotein FrpD binds the RTX protein FrpC. *J Biol Chem,* 280**,** 3251-8.

SEIB, K. L., PIGOZZI, E., MUZZI, A., GAWTHORNE, J. A., DELANY, I., JENNINGS, M. P. & RAPPUOLI, R. 2011. A novel epigenetic regulator associated with the hypervirulent Neisseria meningitidis clonal complex 41/44. *Faseb Journal,* 25**,** 3622-3633.

SHIH, G. C., KAHLER, C. M., CARLSON, R. W., RAHMAN, M. M. & STEPHENS, D. S. 2001. gmhX, a novel gene required for the incorporation of L-glycero-D-manno-heptose into lipooligosaccharide in Neisseria meningitidis. *Microbiology (Reading),* 147**,** 2367-2377.

SIGURLASDOTTIR, S., WASSING, G. M., ZUO, F., ARTS, M. & JONSSON, A. B. 2019. Deletion of D-Lactate Dehydrogenase A in Neisseria meningitidis Promotes Biofilm Formation Through Increased Autolysis and Extracellular DNA Release. *Front Microbiol,* 10**,** 422.

SRIKHANTA, Y. N., DOWIDEIT, S. J., EDWARDS, J. L., FALSETTA, M. L., WU, H. J., HARRISON, O. B., FOX, K. L., SEIB, K. L., MAGUIRE, T. L., WANG, A. H., MAIDEN, M. C., GRIMMOND, S. M., APICELLA, M. A. & JENNINGS, M. P. 2009. Phasevarions mediate random switching of gene expression in pathogenic Neisseria. *PLoS Pathog,* 5**,** e1000400.

STANGER, F. V., BURMANN, B. M., HARMS, A., ARAGAO, H., MAZUR, A., SHARPE, T., DEHIO, C., HILLER, S. & SCHIRMER, T. 2016. Intrinsic regulation of FIC-domain AMP-transferases by oligomerization and automodification. *Proc Natl Acad Sci U S A,* 113**,** E529-37.

TETTELIN, H., SAUNDERS, N. J., HEIDELBERG, J., JEFFRIES, A. C., NELSON, K. E., EISEN, J. A., KETCHUM, K. A., HOOD, D. W., PEDEN, J. F. & DODSON, R. J. 2000. Complete genome sequence of *Neisseria meningitidis* serogroup B strain MC58. *Science*, 287**,** 1809-1815.

THOMPSON, S. A., WANG, L. L., WEST, A. & SPARLING, P. F. 1993. Neisseria meningitidis produces iron-regulated proteins related to the RTX family of exoproteins. *J Bacteriol*, 175**,** 811-8.

TURNER, D. P., MARIETOU, A. G., JOHNSTON, L., HO, K. K., ROGERS, A. J., WOOLDRIDGE, K. G. & ALA'ALDEEN, D. A. 2006. Characterization of MspA, an immunogenic autotransporter protein that mediates adhesion to epithelial and endothelial cells in Neisseria meningitidis. *Infect Immun,* 74**,** 2957-64.

WARREN, M. J., RODDAM, L. F., POWER, P. M., TERRY, T. D. & JENNINGS, M. P. 2004. Analysis of the role of pglI in pilin glycosylation of *Neisseria meningitidis*. *FEMS Immunology & Medical Microbiology*, 41**,** 43-50.

WRIGHT, J. C., HOOD, D. W., RANDLE, G. A., MAKEPEACE, K., COX, A. D., LI, J., CHALMERS, R., RICHARDS, J. C. & MOXON, E. R. 2004. lpt6, a gene required for addition of phosphoethanolamine to inner-core lipopolysaccharide of Neisseria meningitidis and Haemophilus influenzae. *J Bacteriol,* 186**,** 6970-82.

YAN, N. 2013. Structural advances for the major facilitator superfamily (MFS) transporters. *Trends in biochemical sciences*, 38**,** 151-159.

ZHANG, D., IYER, L. M. & ARAVIND, L. 2011. A novel immunity system for bacterial nucleic acid degrading toxins and its recruitment in various eukaryotic and DNA viral systems. *Nucleic Acids Res,* 39**,** 4532-52.

Supplementary Table 3. List of strains from PubMLST database used in the Phaser analysis.

PubMLST ID	Isolate name	Country of isolation	Collection date	Serogroup group	Sequence Type	Clonal Complex
7	7891	Finland	1975	A	5	ST-5 complex
24	S4355	Denmark	1974	A	5	ST-5 complex
30	14	Germany	1999	cnl	53	ST-53 complex
82	11-004	China	1984	A	5	ST-5 complex
84	IAL2229	Brazil	1976	A	5	ST-5 complex
128	F6124	Chad	1988	A	5	ST-5 complex
210	H1964	UK	1987	A	5	ST-5 complex
237	H44/76	Norway	1976	B	32	ST-32 complex
238	153	China	1966	A	5	ST-5 complex
239	154	China	1966	A	6	ST-5 complex
240	MC58	UK	1983	B	74	ST-32 complex
340	196/87	Norway	1987	C	32	ST-32 complex
343	500	Italy	1984	C	11	ST-11 complex
349	38VI	USA	1964	B	11	ST-11 complex
369	M597	Israel	1988	C	11	ST-11 complex
391	90/18311	UK [Scotland]	1990	C	11	ST-11 complex
400	BZ 83	The Netherlands	1984	B	34	ST-32 complex
403	BZ 147	The Netherlands	1963	B	48	ST-41/44 complex
408	BZ 169	The Netherlands	1985	B	32	ST-32 complex

Isolate ID	Strain name	Country of isolation	Collection date	Serogroup group	Sequence Type	Clonal Complex
409	BZ 198	The Netherlands	1986	B	41	ST-41/44 complex
415	EG 329	Germany	1985	B	32	ST-32 complex
419	NG 6/88	Norway	1988	B	13	ST-269 complex
430	NG 080	Norway	1981	B	32	ST-32 complex
431	NG144/82	Norway	1982	B	32	ST-32 complex
434	NG PB24	Norway	1985	B	32	ST-32 complex
441	8680	Chile	1987	B	32	ST-32 complex
451	14/1455	Russia	1970	A	5	ST-5 complex
468	BRAZ10	Brazil	1976	C	11	ST-11 complex
507	MA-5756	Spain	1985	C	11	ST-11 complex
597	92001	China	1992	A	7	ST-5 complex
644	L93/4286	UK [England]	1993	C	11	ST-11 complex
645	204/92	Cuba	1992	B	33	ST-32 complex
646	400	Austria	1991	B	40	ST-41/44 complex
647	AK50	Greece	1992	B	41	ST-41/44 complex
648	M-101/93	Iceland	1993	B	41	ST-41/44 complex
649	50/94	Norway	1994	B	45	ST-41/44 complex
650	M40/94	Chile	1994	B	41	ST-41/44 complex
651	931905	The Netherlands	1993	B	41	ST-41/44 complex
652	N45/96	Norway	1996	B	41	ST-41/44 complex
653	91/40	New Zealand	1991	B	42	ST-41/44 complex
660	71/94	Norway	1994	Y	23	ST-23 complex
662	2837	UK	1997	C	50	ST-11 complex
670	2840	UK	1997	C	50	ST-11 complex

Isolate ID	Strain name	Country of isolation	Collection date	Serogroup group	Sequence Type	Clonal Complex
671	2844	UK	1997	C	50	ST-11 complex
672	2847	UK	1997	C	50	ST-11 complex
684	0259/93	Czech Republic	1993	C	11	ST-11 complex
698	FAM18	USA	1983	C	11	ST-11 complex
972	0066/93	Czech Republic	1993	E	53	ST-53 complex
984	0083/93	Czech Republic	1993	E	53	ST-53 complex
1168	BM33	Greece	1996	B	33	ST-32 complex
1169	BM45	Greece	1996	C	11	ST-11 complex
1178	W72	Greece	1997	C	211	ST-11 complex
1181	W138	Greece	1998	B	11	ST-11 complex
1597	0206/93	Czech Republic	1993	E	122	ST-53 complex
1639	0252/93	Czech Republic	1993	E	53	ST-53 complex
1649	0269/93	Czech Republic	1993	E	53	ST-53 complex
1654	0277/93	Czech Republic	1993	cnl	123	ST-53 complex
1955	0011/93	Czech Republic	1993	B	740	ST-41/44 complex
2216	0190/93	Czech Republic	1993	B	998	ST-269 complex
2222	0314/93	Czech Republic	1993	cnl	124	ST-53 complex
2233	0531/93	Czech Republic	1993	B	1015	ST-32 complex
2281	M7089	USA	2000	W	11	ST-11 complex
2290	M7124	Saudi Arabia	2000	W	11	ST-11 complex
4145	0148/00	Czech Republic	2000	B	2994	ST-11 complex
5171	0085/00	Czech Republic	2000	B	3537	ST-11 complex
6986	0131/95	Czech Republic	1995	B	4993	ST-11 complex
7891	0179/04	Czech Republic	2004	B	5756	ST-11 complex
7892	0263/04	Czech Republic	2004	B	5757	ST-11 complex

Isolate ID	Strain name	Country of isolation	Collection date	Serogroup group	Sequence Type	Clonal Complex
8169	0410/93	Czech Republic	1993	cnl	53	ST-53 complex
8177	0422/93	Czech Republic	1993	cnl	53	ST-53 complex
8181	0426/93	Czech Republic	1993	cnl	53	ST-53 complex
8682	0030/01	Czech Republic	2001	B	11	ST-11 complex
10436	0290/94	Czech Republic	1994	W	247	ST-11 complex
10479	0233/96	Czech Republic	1996	B	11	ST-11 complex
10487	0288/96	Czech Republic	1996	B	11	ST-11 complex
10499	0467/96	Czech Republic	1996	B	11	ST-11 complex
10500	0492/96	Czech Republic	1996	B	11	ST-11 complex
10705	N 25/99	Norway	1999	B	44	ST-41/44 complex
10735	N 56/99	Norway	1999	B	45	ST-41/44 complex
10756	8/00	Norway	2000	B	43	ST-41/44 complex
10758	10/00	Norway	2000	B	110	ST-41/44 complex
10812	64/00	Norway	2000	B	146	ST-41/44 complex
11389	2379	South Africa	2005	B	6697	ST-41/44 complex
13420	0106/07	Czech Republic	2007	B	11	ST-11 complex
14480	M5178	USA	1998	B	32	ST-32 complex
14627	M20918	USA	2009	A	47,897,980	ST-5 complex
14765	0002/93	Czech Republic	1993	C	11	ST-11 complex
15032	0186/93	Czech Republic	1993	C	11	ST-11 complex
15035	0188/93	Czech Republic	1993	C	11	ST-11 complex
15152	0263/93	Czech Republic	1993	discrepancy	11	ST-11 complex
15154	0264/93	Czech Republic	1993	C	11	ST-11 complex
15174	0284/93	Czech Republic	1993	C	11	ST-11 complex
15176	0287/93	Czech Republic	1993	C	11	ST-11 complex

Isolate ID	Strain name	Country of isolation	Collection date	Serogroup group	Sequence Type	Clonal Complex
15238	0343/93	Czech Republic	1993	C	11	ST-11 complex
15242	0351/93	Czech Republic	1993	C	11	ST-11 complex
15249	0379/93	Czech Republic	1993	B	118	ST-32 complex
15261	0400/93	Czech Republic	1993	C	11	ST-11 complex
15313	0469/93	Czech Republic	1993	B	118	ST-32 complex
15315	0489/93	Czech Republic	1993	C	11	ST-11 complex
15316	0490/93	Czech Republic	1993	C	11	ST-11 complex
15319	0500/93	Czech Republic	1993	C	11	ST-11 complex
15325	0512/93	Czech Republic	1993	C	11	ST-11 complex
15330	0520/93	Czech Republic	1993	C	11	ST-11 complex
15336	0529/93	Czech Republic	1993	C	11	ST-11 complex
15342	0535/93	Czech Republic	1993	C	11	ST-11 complex
15344	0538/93	Czech Republic	1993	C	11	ST-11 complex
15623	0091/04	Czech Republic	2004	B	5756	ST-11 complex
15630	0140/04	Czech Republic	2004	B	5756	ST-11 complex
18968	M10 240474	UK [England]	2010	B	269	ST-269 complex
18969	M10 240478	UK [England]	2010	B	1194	ST-41/44 complex
19023	M10 240473	UK [England]	2010	B	1161	ST-269 complex
19024	M10 240480	UK [England]	2010	B	1194	ST-41/44 complex
19026	M10 240482	UK [England]	2010	B	3754	ST-41/44 complex
19027	M10 240484	UK [England]	2010	B	1097	ST-41/44 complex
19028	M10 240485	UK [England]	2010	B	275	ST-269 complex
19029	M10 240487	UK [England]	2010	B	1049	ST-269 complex
19030	M10 240489	UK [England]	2010	B	461	ST-461 complex
19031	M10 240490	UK [England]	2010	B	1161	ST-269 complex

Isolate ID	Strain name	Country of isolation	Collection date	Serogroup group	Sequence Type	Clonal Complex
19032	M10 240498	UK [Wales]	2010	B	41	ST-41/44 complex
19260	WUE 2594	Germany	1991	A	5	ST-5 complex
19261	alpha710	Germany	2012	B	136	ST-41/44 complex
19263	NZ-05/33	New Zealand	2005	B	42	ST-41/44 complex
19264	M04-240196	UK	2004	B	269	ST-269 complex
19265	M01-240355	UK	2001	B	213	ST-213 complex
19266	M01-240149	UK	2001	B	41	ST-41/44 complex
19359	M13399	Unknown	2005	B	2976	ST-269 complex
19362	CU385	Unknown	1980	B	33	ST-32 complex
19366	M10699	USA	2003	B	32	ST-32 complex
19367	M11791	USA	2003	Y	23	ST-23 complex
19368	M13220	Philippines	2005	A	7	ST-5 complex
19370	M14900	USA	2006	Y	1625	ST-23 complex
19372	M15293	USA	2006	NG	32	ST-32 complex
19378	M18575	Burkina Faso	2003	A	2859	ST-5 complex
19958	M10 240476	UK [England]	2010	B	9812	ST-213 complex
19959	M10 240499	UK [England]	2010	B	275	ST-269 complex
19960	M10 240500	UK [England]	2010	B	41	ST-41/44 complex
19961	M10 240502	UK [England]	2010	B	340	ST-41/44 complex
19962	M10 240503	UK [England]	2010	Y	23	ST-23 complex
19963	M10 240505	UK [England]	2010	Y	1655	ST-23 complex
19964	M10 240507	UK [England]	2010	Y	183	ST-23 complex
19967	M10 240512	UK [England]	2010	B	41	ST-41/44 complex
19969	M10 240515	UK [England]	2010	B	269	ST-269 complex
19970	M10 240520	UK [England]	2010	Y	1655	ST-23 complex

Isolate ID	Strain name	Country of isolation	Collection date	Serogroup group	Sequence Type	Clonal Complex
19971	M10 240521	UK [England]	2010	B	3802	ST-41/44 complex
19972	M10 240522	UK [England]	2010	B	41	ST-41/44 complex
19973	M10 240525	UK [England]	2010	B	2931	ST-32 complex
19974	M10 240527	UK [England]	2010	B	269	ST-269 complex
19975	M10 240528	UK [England]	2010	B	7833	ST-269 complex
19976	M10 240530	UK [England]	2010	Y	9813	ST-23 complex
19977	M10 240531	UK [England]	2010	Y	1655	ST-23 complex
19978	M10 240532	UK [England]	2010	B	41	ST-41/44 complex
19979	M10 240534	UK [England]	2010	Y	1655	ST-23 complex
19980	M10 240536	UK [England]	2010	Y	1655	ST-23 complex
19981	M10 240540	UK [England]	2010	Y	9814	ST-23 complex
19982	M10 240546	UK [England]	2010	B	213	ST-213 complex
19983	M10 240547	UK [England]	2010	B	749	ST-32 complex
19984	M10 240548	UK [England]	2010	B	340	ST-41/44 complex
19985	M10 240549	UK [Wales]	2010	B	41	ST-41/44 complex
19986	M10 240550	UK [England]	2010	B	1163	ST-269 complex
19987	M10 240553	UK [Wales]	2010	B	1161	ST-269 complex
19988	M10 240556	UK [England]	2010	B	9815	ST-41/44 complex
19989	M10 240558	UK [Wales]	2010	B	1992	ST-41/44 complex
19991	M10 240566	UK [England]	2010	B	213	ST-213 complex
19992	M10 240568	UK [England]	2010	B	213	ST-213 complex
19994	M10 240570	UK [England]	2010	B	5357	ST-41/44 complex
19995	M10 240572	UK [England]	2010	B	1161	ST-269 complex
19996	M10 240578	UK [England]	2010	B	34	ST-32 complex
19998	M10 240580	UK [England]	2010	Y	1655	ST-23 complex

Isolate ID	Strain name	Country of isolation	Collection date	Serogroup group	Sequence Type	Clonal Complex
20000	M10 240582	UK [England]	2010	B	34	ST-32 complex
20002	M10 240585	UK [England]	2010	B	46	ST-41/44 complex
20003	M10 240586	UK [England]	2010	B	485	ST-41/44 complex
20004	M10 240587	UK [England]	2010	B	9879	ST-41/44 complex
20005	M10 240589	UK [England]	2010	B	40	ST-41/44 complex
20006	M10 240590	UK [England]	2010	Y	23	ST-23 complex
20007	M10 240591	UK [England]	2010	B	461	ST-461 complex
20008	M10 240592	UK [England]	2010	B	6782	ST-41/44 complex
20009	M10 240595	UK [England]	2010	B	213	ST-213 complex
20010	M10 240597	UK [England]	2010	B	41	ST-41/44 complex
20011	M10 240598	UK [England]	2010	B	41	ST-41/44 complex
20012	M10 240602	UK [England]	2010	B	1161	ST-269 complex
20013	M10 240604	UK [Northern Ireland]	2010	B	8384	ST-41/44 complex
20014	M10 240605	UK [England]	2010	B	41	ST-41/44 complex
20015	M10 240606	UK [England]	2010	B	213	ST-213 complex
20017	M10 240611	UK [England]	2010	B	749	ST-32 complex
20018	M10 240612	UK [England]	2010	B	269	ST-269 complex
20019	M10 240613	UK [England]	2010	B	1161	ST-269 complex
20021	M10 240616	UK [England]	2010	W	1224	ST-22 complex
20022	M10 240617	UK [England]	2010	B	213	ST-213 complex
20023	M10 240618	UK [England]	2010	E	2435	ST-60 complex
20024	M10 240619	UK [England]	2010	B	213	ST-213 complex
20025	M10 240621	UK [Wales]	2010	B	213	ST-213 complex
20027	M10 240623	UK [England]	2010	B	1161	ST-269 complex
20028	M10 240624	UK [England]	2010	B	275	ST-269 complex

Isolate ID	Strain name	Country of isolation	Collection date	Serogroup group	Sequence Type	Clonal Complex
20029	M10 240626	UK [England]	2010	B	4401	ST-269 complex
20030	M10 240627	UK [England]	2010	B	6083	ST-32 complex
20031	M10 240631	UK [England]	2010	C	467	ST-269 complex
20032	M10 240632	UK [England]	2010	Y	1655	ST-23 complex
20033	M10 240633	UK [England]	2010	Y	1655	ST-23 complex
20034	M10 240634	UK [Northern Ireland]	2010	B	42	ST-41/44 complex
20036	M10 240638	UK [England]	2010	B	275	ST-269 complex
20039	M10 240641	UK [England]	2010	W	184	ST-22 complex
20041	M10 240643	UK [England]	2010	Y	23	ST-23 complex
20042	M10 240644	UK [England]	2010	Y	3651	ST-22 complex
20043	M10 240645	UK [England]	2010	B	283	ST-269 complex
20044	M10 240649	UK [England]	2010	B	275	ST-269 complex
20046	M10 240651	UK [England]	2010	B	9881	ST-213 complex
20047	M10 240652	UK [England]	2010	B	275	ST-269 complex
20049	M10 240659	UK [England]	2010	B	41	ST-41/44 complex
20050	M10 240661	UK [England]	2010	B	1161	ST-269 complex
20051	M10 240662	UK [England]	2010	B	9882	ST-41/44 complex
20052	M10 240665	UK [England]	2010	B	213	ST-213 complex
20055	M10 240669	UK [England]	2010	B	1992	ST-41/44 complex
20056	M10 240670	UK [England]	2010	B	269	ST-269 complex
20058	M10 240674	UK [England]	2010	B	41	ST-41/44 complex
20064	M10 240682	UK [Northern Ireland]	2010	B	269	ST-269 complex
20067	M10 240685	UK [England]	2010	Y	1655	ST-23 complex
20068	M10 240687	UK [England]	2010	B	60	ST-60 complex

Isolate ID	Strain name	Country of isolation	Collection date	Serogroup group	Sequence Type	Clonal Complex
20071	M10 240693	UK [England]	2010	B	1430	ST-60 complex
20074	M10 240700	UK [England]	2010	Y	6463	ST-23 complex
20075	M10 240701	UK [England]	2010	B	213	ST-213 complex
20077	M10 240703	UK [England]	2010	B	9884	ST-213 complex
20078	M10 240704	UK [England]	2010	B	269	ST-269 complex
20081	M10 240708	UK [England]	2010	B	1195	ST-269 complex
20082	M10 240709	UK [England]	2010	B	479	ST-269 complex
20083	M10 240711	UK [England]	2010	B	275	ST-269 complex
20084	M10 240712	UK [England]	2010	B	213	ST-213 complex
20085	M10 240713	UK [England]	2010	B	9829	ST-269 complex
20086	M10 240716	UK [England]	2010	B	461	ST-461 complex
20087	M10 240717	UK [England]	2010	B	9830	ST-213 complex
20088	M10 240718	UK [England]	2010	B	9885	ST-60 complex
20089	M10 240719	UK [England]	2010	B	34	ST-32 complex
20090	M10 240720	UK [England]	2010	B	479	ST-269 complex
20095	M10 240728	UK [England]	2010	B	9840	ST-269 complex
20096	M10 240729	UK [England]	2010	B	1049	ST-269 complex
20099	M10 240732	UK [England]	2010	Y	23	ST-23 complex
20101	M10 240737	UK [England]	2010	W	1281	ST-22 complex
20102	M10 240742	UK [England]	2010	B	8955	ST-213 complex
20103	M10 240743	UK [England]	2010	B	269	ST-269 complex
20104	M10 240745	UK [England]	2010	Y	1655	ST-23 complex
20110	M10 240751	UK [England]	2010	B	213	ST-213 complex
20111	M10 240752	UK [England]	2010	B	1161	ST-269 complex
20112	M10 240753	UK [England]	2010	B	34	ST-32 complex

Isolate ID	Strain name	Country of isolation	Collection date	Serogroup group	Sequence Type	Clonal Complex
20114	M10 240756	UK [England]	2010	W	2114	ST-22 complex
20119	M10 240766	UK [England]	2010	B	479	ST-269 complex
20120	M10 240767	UK [Wales]	2010	B	34	ST-32 complex
20121	M10 240768	UK [England]	2010	B	2873	ST-269 complex
20122	M10 240770	UK [England]	2010	B	1195	ST-269 complex
20124	M10 240772	UK [England]	2010	B	1161	ST-269 complex
20126	M10 240774	UK [Wales]	2010	B	1161	ST-269 complex
20128	M10 240776	UK [England]	2010	Y	1655	ST-23 complex
20129	M10 240777	UK [England]	2010	Y	23	ST-23 complex
20130	M10 240778	UK [Wales]	2010	B	9187	ST-213 complex
20131	M10 240779	UK [England]	2010	B	269	ST-269 complex
20132	M10 240781	UK [England]	2010	Y	3651	ST-22 complex
20133	M10 240783	UK [England]	2010	B	1161	ST-269 complex
20135	M10 240786	UK [England]	2010	Y	9831	ST-23 complex
20136	M10 240787	UK [England]	2010	Y	1655	ST-23 complex
20138	M10 240789	UK [Wales]	2010	B	283	ST-269 complex
20139	M10 240790	UK [England]	2010	B	259	ST-32 complex
20141	M10 240794	UK [England]	2010	B	32	ST-32 complex
20143	M10 240798	UK [England]	2010	Y	1655	ST-23 complex
20144	M10 240799	UK [England]	2010	B	213	ST-213 complex
20146	M10 240804	UK [England]	2010	Y	1655	ST-23 complex
20149	M10 240808	UK [England]	2010	B	1092	ST-269 complex
20153	M10 240814	UK [Wales]	2010	B	1161	ST-269 complex
20159	M10 240822	UK [England]	2010	W	1224	ST-22 complex
20161	M10 240824	UK [England]	2010	B	32	ST-32 complex

Isolate ID	Strain name	Country of isolation	Collection date	Serogroup group	Sequence Type	Clonal Complex
20162	M10 240825	UK [England]	2010	B	9818	ST-60 complex
20164	M11 240000	UK [England]	2011	Y	23	ST-23 complex
20165	M11 240001	UK [England]	2011	B	1946	ST-461 complex
20169	M11 240005	UK [England]	2011	Y	23	ST-23 complex
20170	M11 240006	UK [Wales]	2011	B	8049	ST-32 complex
20171	M11 240007	UK [England]	2011	Y	1655	ST-23 complex
20176	M11 240013	UK [England]	2011	B	9890	ST-32 complex
20177	M11 240014	UK [England]	2011	B	9891	ST-461 complex
20179	M11 240016	UK [Wales]	2011	B	1096	ST-32 complex
20183	M11 240021	UK [England]	2011	B	259	ST-32 complex
20189	M11 240027	UK [England]	2011	B	60	ST-60 complex
20190	M11 240028	UK [England]	2011	B	60	ST-60 complex
20191	M11 240029	UK [England]	2011	W	1286	ST-22 complex
20193	M11 240031	UK [England]	2011	Y	1655	ST-23 complex
20197	M11 240036	UK [Wales]	2011	Y	1655	ST-23 complex
20198	M11 240037	UK [England]	2011	B	34	ST-32 complex
20203	M11 240042	UK [England]	2011	B	1946	ST-461 complex
20204	M11 240043	UK [England]	2011	Y	9842	ST-23 complex
20207	M11 240046	UK [England]	2011	B	213	ST-213 complex
20209	M11 240048	UK [England]	2011	B	60	ST-60 complex
20210	M11 240050	UK [England]	2011	B	213	ST-213 complex
20211	M11 240052	UK [Wales]	2011	B	461	ST-461 complex
20220	M11 240061	UK [England]	2011	B	9833	ST-213 complex
20224	M11 240065	UK [England]	2011	Y	1655	ST-23 complex
20233	M11 240074	UK [Wales]	2011	B	213	ST-213 complex

Isolate ID	Strain name	Country of isolation	Collection date	Serogroup group	Sequence Type	Clonal Complex
20238	M11 240082	UK [England]	2011	B	290	ST-32 complex
20239	M11 240083	UK [England]	2011	B	461	ST-461 complex
20245	M11 240096	UK [England]	2011	Y	1655	ST-23 complex
20250	M11 240109	UK [England]	2011	B	2931	ST-32 complex
20252	M11 240111	UK [England]	2011	B	213	ST-213 complex
20254	M11 240113	UK [England]	2011	B	2666	ST-213 complex
20256	M11 240116	UK [England]	2011	Y	1655	ST-23 complex
20257	M11 240117	UK [England]	2011	B	9821	ST-60 complex
20259	M11 240121	UK [England]	2011	B	213	ST-213 complex
20264	M11 240126	UK [England]	2011	Y	23	ST-23 complex
20270	M11 240134	UK [England]	2011	B	213	ST-213 complex
20271	M11 240137	UK [England]	2011	B	213	ST-213 complex
20275	M11 240147	UK [England]	2011	B	213	ST-213 complex
20281	M11 240157	UK [England]	2011	Y	10138	ST-23 complex
20286	M11 240166	UK [England]	2011	Y	23	ST-23 complex
20287	M11 240167	UK [England]	2011	B	9327	ST-60 complex
20292	M11 240175	UK [England]	2011	B	33	ST-32 complex
20295	M11 240181	UK [England]	2011	B	213	ST-213 complex
20297	M11 240183	UK [England]	2011	B	32	ST-32 complex
20302	M11 240192	UK [Wales]	2011	Y	1655	ST-23 complex
20306	M11 240206	UK [England]	2011	B	461	ST-461 complex
20309	M11 240210	UK [England]	2011	Y	23	ST-23 complex
20313	M11 240214	UK [England]	2011	B	1946	ST-461 complex
20314	M11 240215	UK [England]	2011	Y	1655	ST-23 complex
20315	M11 240216	UK [England]	2011	Y	1655	ST-23 complex

Isolate ID	Strain name	Country of isolation	Collection date	Serogroup group	Sequence Type	Clonal Complex
20317	M11 240227	UK [England]	2011	Y	6463	ST-23 complex
20318	M11 240231	UK [England]	2011	W	184	ST-22 complex
20328	M11 240246	UK [England]	2011	E	60	ST-60 complex
20335	M11 240258	UK [England]	2011	Y	23	ST-23 complex
20337	M11 240261	UK [England]	2011	W	184	ST-22 complex
20338	M11 240262	UK [England]	2011	A	4789	ST-5 complex
20339	M11 240263	UK [England]	2011	Y	1655	ST-23 complex
20341	M11 240266	UK [England]	2011	B	213	ST-213 complex
20342	M11 240268	UK [England]	2011	B	213	ST-213 complex
20346	M11 240277	UK [England]	2011	Y	1655	ST-23 complex
20347	M11 240278	UK [England]	2011	Y	1655	ST-23 complex
20350	M11 240282	UK [England]	2011	B	213	ST-213 complex
20351	M11 240283	UK [England]	2011	Y	23	ST-23 complex
20353	M11 240285	UK [England]	2011	B	33	ST-32 complex
20357	M11 240290	UK [England]	2011	B	9895	ST-213 complex
20361	M11 240296	UK [England]	2011	B	34	ST-32 complex
20362	M11 240297	UK [England]	2011	B	213	ST-213 complex
20363	M11 240298	UK [England]	2011	Y	1655	ST-23 complex
20365	M11 240302	UK [England]	2011	W	184	ST-22 complex
20382	M11 240324	UK [England]	2011	C	32	ST-32 complex
20389	M11 240337	UK [England]	2011	B	213	ST-213 complex
20391	M11 240339	UK [England]	2011	B	60	ST-60 complex
20393	M11 240342	UK [England]	2011	B	32	ST-32 complex
20395	M11 240344	UK [England]	2011	B	213	ST-213 complex
20398	M11 240348	UK [England]	2011	B	213	ST-213 complex

Isolate ID	Strain name	Country of isolation	Collection date	Serogroup group	Sequence Type	Clonal Complex
20399	M11 240349	UK [England]	2011	W	22	ST-22 complex
20403	M11 240356	UK [England]	2011	B	32	ST-32 complex
20409	M11 240366	UK [Northern Ireland]	2011	B	33	ST-32 complex
20422	M11 240387	UK [England]	2011	W	184	ST-22 complex
20427	M11 240392	UK [England]	2011	B	290	ST-32 complex
20428	M11 240394	UK [England]	2011	B	5103	ST-60 complex
20438	M11 240406	UK [England]	2011	B	32	ST-32 complex
20441	M11 240412	UK [England]	2011	B	1430	ST-60 complex
20457	M11 240440	UK [England]	2011	B	60	ST-60 complex
20477	H44/76	Norway	1976	B	32	ST-32 complex
20564	Nm6938	Canada	2001	W	22	ST-22 complex
20565	Nm2732	Canada	2007	W	22	ST-22 complex
20740	M09 240044	UK	2013	B	32	ST-32 complex
20741	M09 240064	UK	2013	B	7788	ST-60 complex
20753	M09 240242	UK	2013	B	461	ST-461 complex
20759	M09 240296	UK	2013	B	213	ST-213 complex
20763	M09 240440	UK	2013	B	2388	ST-213 complex
20769	M09 240659	UK	2013	B	213	ST-213 complex
20771	M09 240681	UK	2013	B	213	ST-213 complex
20790	M10 240127	UK	2013	B	60	ST-60 complex
20793	M10 240140	UK	2013	B	461	ST-461 complex
20899	PM83TS	UK	2013	B	60	ST-60 complex
20900	PM83B	UK	2013	B	60	ST-60 complex
20903	PM27TS	UK	2013		1281	ST-22 complex
20904	PM27B	UK	2013	W	1281	ST-22 complex

Isolate ID	Strain name	Country of isolation	Collection date	Serogroup group	Sequence Type	Clonal Complex
20912	PM22B	UK	2013	B	2100	ST-213 complex
20921	PM18CSF	UK	2013	B	213	ST-213 complex
20922	PM18TS	UK	2013	B	213	ST-213 complex
20923	PM19TS	UK	2013	B	213	ST-213 complex
20924	PM19B	UK	2013	B	213	ST-213 complex
20925	PM24TS	Ireland	2013	B	5482	ST-213 complex
20926	PM24B	Ireland	2013	B	5482	ST-213 complex
20971	PM84CSF	Isle of Man	2008	B	60	ST-60 complex
20972	PM84TS	Isle of Man	2013	B	60	ST-60 complex
21092	M11 240443	UK [England]	2011	B	461	ST-461 complex
21112	M11 240471	UK [England]	2011	B	60	ST-60 complex
21132	M11 240497	UK [England]	2011	W	1281	ST-22 complex
21139	M11 240508	UK [England]	2011	W	184	ST-22 complex
21156	M11 240716	UK [England]	2011	W	3795	ST-22 complex
21175	M11 240741	UK [England]	2011	W	1286	ST-22 complex
21193	M11 240780	UK [England]	2011	W/Y	3651	ST-22 complex
21228	M11 240988	UK [England]	2011	B	461	ST-461 complex
21246	M11 241031	UK [England]	2011	B	461	ST-461 complex
21271	M11 241064	UK [England]	2011	Y	3651	ST-22 complex
21280	M11 241075	UK [Wales]	2011	B	60	ST-60 complex
21290	M12 240008	UK [England]	2012	W	184	ST-22 complex
21300	M12 240019	UK [England]	2012	B	461	ST-461 complex
21349	M12 240088	UK [England]	2012	W	1224	ST-22 complex
21352	M12 240093	UK [England]	2012	E	4146	ST-60 complex
21371	M12 240121	UK [England]	2012	W	184	ST-22 complex

Isolate ID	Strain name	Country of isolation	Collection date	Serogroup group	Sequence Type	Clonal Complex
21380	M12 240132	UK [England]	2012	W	184	ST-22 complex
21396	M12 240158	UK [England]	2012	B	10287	ST-60 complex
21432	M12 240222	UK [England]	2012	B	10292	ST-60 complex
21454	M12 240251	UK [England]	2012	W	1281	ST-22 complex
21576	SA_serogroup W_NM4	South Africa	2012	W	184	ST-22 complex
26067	959000095	Sweden	1995	W	22	ST-22 complex
26253	T97	UK	2011	cnl	53	ST-53 complex
26263	V259	UK	2011	cnl	53	ST-53 complex
26264	V302	UK	2012	E	60	ST-60 complex
26267	T98	UK	2011	cnl	53	ST-53 complex
26271	BB66	UK	2012	cnl	53	ST-53 complex
26273	BB58	UK	2012	W	184	ST-22 complex
26274	v191	UK	2012	cnl	53	ST-53 complex
26275	BB242	UK	2012	W	22	ST-22 complex
26278	BB122	UK	2012	E	10454	ST-60 complex
26283	R121	UK	2011	cnl	2441	ST-53 complex
26287	V129	UK	2011	W	22	ST-22 complex
26297	T36	UK	2011	E	10457	ST-60 complex
26303	T287	UK	2011	Y	114	ST-22 complex
26306	R279	UK	2011	E	60	ST-60 complex
26308	x268	UK	2012	E	10343	ST-60 complex
26314	z54	UK	2012	E	4146	ST-60 complex
26320	R136	UK	2011	cnl	53	ST-53 complex
26325	x83	UK	2012	cnl	53	ST-53 complex
26326	T304	UK	2011	cnl	53	ST-53 complex

Isolate ID	Strain name	Country of isolation	Collection date	Serogroup group	Sequence Type	Clonal Complex
26327	R122	UK	2011	E	10454	ST-60 complex
26329	BB130	UK	2012	E	4146	ST-60 complex
26332	BB111	UK	2012	B	461	ST-461 complex
26336	v216	UK	2012	cnl	53	ST-53 complex
26344	R243	UK	2011	cnl	53	ST-53 complex
26349	BB279	UK	2012	E	60	ST-60 complex
26350	T3	UK	2011	cnl	53	ST-53 complex
26352	BB36	UK	2012	E	10457	ST-60 complex
26355	R263	UK	2011	cnl	53	ST-53 complex
26360	R221	UK	2011	E	466	ST-60 complex
26365	z191	UK	2012	cnl	53	ST-53 complex
26370	R129	UK	2011	W	22	ST-22 complex
26372	V304	UK	2012	cnl	53	ST-53 complex
26381	R210	UK	2011	X	5799	ST-22 complex
26382	X136	UK	2011	cnl	53	ST-53 complex
26383	R216	UK	2011	cnl	53	ST-53 complex
26390	BB210	UK	2012	X	5799	ST-22 complex
26394	T121	UK	2011	cnl	2441	ST-53 complex
26399	R155	UK	2011	cnl	53	ST-53 complex
26402	R83	UK	2011	cnl	53	ST-53 complex
26406	T122	UK	2011	E	10454	ST-60 complex
26408	BB287	UK	2012	Y	114	ST-22 complex
26409	R222	UK	2011	E	10343	ST-60 complex
26411	R36	UK	2011	E	10457	ST-60 complex
26414	R4	UK	2011	E	60	ST-60 complex

Isolate ID	Strain name	Country of isolation	Collection date	Serogroup group	Sequence Type	Clonal Complex
26415	T191	UK	2011	cnl	53	ST-53 complex
26418	x222	UK	2012	E	10343	ST-60 complex
26419	T119	UK	2011	cnl	53	ST-53 complex
26425	R256	UK	2011	E	466	ST-60 complex
26432	T210	UK	2011	X	5799	ST-22 complex
26434	T302	UK	2011	E	60	ST-60 complex
26444	R97	UK	2011	cnl	53	ST-53 complex
26445	Z119	UK	2012	cnl	53	ST-53 complex
26450	BB159	UK	2012	E	4146	ST-60 complex
26451	r119	UK	2011	cnl	53	ST-53 complex
26457	R191	UK	2011	cnl	53	ST-53 complex
26464	Z221	UK	2012	E	466	ST-60 complex
26466	X119	UK	2012	cnl	53	ST-53 complex
26474	T216	UK	2011	cnl	53	ST-53 complex
26476	R58	UK	2011	W	184	ST-22 complex
26498	T279	UK	2011	E	60	ST-60 complex
26500	BB83	UK	2012	cnl	53	ST-53 complex
26515	BB54	UK	2012	E	4146	ST-60 complex
26518	v119	UK	2012	cnl	53	ST-53 complex
26519	T53	UK	2011	E	10454	ST-60 complex
26520	T4	UK	2011	E	60	ST-60 complex
26522	x121	UK	2012	cnl	2441	ST-53 complex
26529	BB228	UK	2012	cnl	53	ST-53 complex
26530	V210	UK	2012	X	5799	ST-22 complex
26839	12005_2011	Ireland	2011	B	8949	ST-461 complex

Isolate ID	Strain name	Country of isolation	Collection date	Serogroup group	Sequence Type	Clonal Complex
26847	12018_2011	Ireland	2011	B	1946	ST-461 complex
26888	12029_2012	Ireland	2012	B	10548	ST-22 complex
26900	12004_2013	Ireland	2013	W	22	ST-22 complex
26922	10215v3	UK	2013	W	22	ST-22 complex
26923	10215v4	UK	2013		11141	ST-22 complex
26954	80241v5	UK	2013	W	184	ST-22 complex
26955	80305v4	UK	2013	W	184	ST-22 complex
26956	80305v5	UK	2013	W	184	ST-22 complex
26976	90104v5	UK	2013	W	3651	ST-22 complex
26985	90130v1	UK	2013	W	184	ST-22 complex
27782	M12 240347	UK [England]	2012	B	461	ST-461 complex
27814	M12 240704	UK [England]	2012	B	7261	ST-461 complex
27839	M12 240763	UK [England]	2012	B	1946	ST-461 complex
27843	M12 240778	UK [England]	2012	B	1946	ST-461 complex
27849	M12 240787	UK [Wales]	2012	B	1946	ST-461 complex
27864	M12 240819	UK [England]	2012	B	461	ST-461 complex
27871	M12 240844	UK [England]	2012	B	8223	ST-461 complex
27887	M12 240873	UK [Wales]	2012	B	10705	ST-461 complex
27907	M13 240006	UK [England]	2013	B	461	ST-461 complex
27914	M13 240021	UK [England]	2013	B	461	ST-461 complex
27927	M13 240045	UK [England]	2013	B	1946	ST-461 complex
27933	M13 240057	UK [England]	2013	B	5983	ST-461 complex
27934	M13 240063	UK [England]	2013	B	461	ST-461 complex
27938	M13 240074	UK [England]	2013	B	461	ST-461 complex
27941	M13 240078	UK [England]	2013	B	1946	ST-461 complex

Isolate ID	Strain name	Country of isolation	Collection date	Serogroup group	Sequence Type	Clonal Complex
28017	M13 240248	UK [England]	2013	B	1946	ST-461 complex
28043	M13 240406	UK [England]	2013	B	1946	ST-461 complex
29291	15003	South Africa	2004	A	7	ST-5 complex
29304	27937	South Africa	2010	A	7	ST-5 complex
29312	5957	South Africa	2006	cnl	53	ST-53 complex
29434	139	South Africa	2005	A	7	ST-5 complex
29469	2730	UK	2014	A	5	ST-5 complex
30269	M99 243594	UK	1999	A	5	ST-5 complex
30466	NMA510612	China	2006	A	7	ST-5 complex
30697	12011-10	Ireland	2010	B	461	ST-461 complex
30708	12021-13	Ireland	2013	B	7261	ST-461 complex
31208	NM10853	UK [England]	2013	cnl	53	ST-53 complex
31215	LIM534	France	2006	A	4789	ST-5 complex
34572	75643	Brazil	1975	A	5	ST-5 complex
34573	75689	Brazil	1975	A	5	ST-5 complex
34578	88050	Chad	1988	A	5	ST-5 complex
34583	96023	Niger	1996	A	5	ST-5 complex
34584	96024	Niger	1996	A	5	ST-5 complex
34587	97008	Niger	1997	A	5	ST-5 complex
34589	97018	Mali	1997	A	580	ST-5 complex
34590	97020	Mali	1997	A	580	ST-5 complex
34594	98005	Niger	1998	A	5	ST-5 complex
34595	98008	France	1998	discrepancy	461	ST-461 complex
34597	2000063	Niger	2000	A	7	ST-5 complex
34598	2000080	Algeria	2000	A	7	ST-5 complex

Isolate ID	Strain name	Country of isolation	Collection date	Serogroup group	Sequence Type	Clonal Complex
34599	2001212	Burkina Faso	2001	A	7	ST-5 complex
34600	2002007	Niger	2002	A	7	ST-5 complex
34604	2003022	Niger	2003	A	7	ST-5 complex
34606	2004085	Niger	2004	A	7	ST-5 complex
34607	2004090	Niger	2004	A	7	ST-5 complex
34610	2007056	Burkina Faso	2007	A	2859	ST-5 complex
34654	NM3642	Bangladesh	2003	A	4789	ST-5 complex
34655	NM3652	Bangladesh	2006	A	8428	ST-5 complex
34663	NM604	USA	2000	A	7	ST-5 complex
34665	NM607	USA	2001	A	7	ST-5 complex
34808	ERR052799	Burkina Faso	2006	A	2859	ST-5 complex
34811	ERR052816	Burkina Faso	2007	A	2859	ST-5 complex
34818	ERR052800	Burkina Faso	2006	A	2859	ST-5 complex
34822	ERR052787	Burkina Faso	2006	A	2859	ST-5 complex
34825	ERR052767	Ghana	2004	A	7	ST-5 complex
34836	ERR052737	Ghana	2001	A	7	ST-5 complex
34837	ERR052779	Ghana	2004	A	7	ST-5 complex
35330	12.1192.H	UK [Scotland]	2012	B	1946	ST-461 complex
35345	13.2973.F	UK [Scotland]	2013	B	461	ST-461 complex
35346	13.3065.D	UK [Scotland]	2013	B	461	ST-461 complex
35427	M13 240534	UK [England]	2013	B	1946	ST-461 complex
35436	M13 240554	UK [England]	2013	B	461	ST-461 complex
35448	M13 240581	UK [England]	2013	B	1946	ST-461 complex
35480	M13 240644	UK [England]	2013	B	461	ST-461 complex

**Isolate ID**	**Strain name**	**Country of isolation**	**Collection date**	**Serogroup group**	**Sequence Type**	**Clonal Complex**
35577	M14 240083	UK [Northern Ireland]	2014	B	11865	ST-461 complex
35580	M14 240088	UK [England]	2014	B	461	ST-461 complex
35612	M14 240133	UK [Wales]	2014	B	1946	ST-461 complex
35713	M14 240317	UK [England]	2014	B	1946	ST-461 complex
36135	12010_2012	Ireland	2012	cnl	53	ST-53 complex
38889	P27	Italy	2012	cnl	53	ST-53 complex
38943	P106	Italy	2013	cnl	53	ST-53 complex
38946	P109	Italy	2013	cnl	53	ST-53 complex
38953	P117	Italy	2013	cnl	53	ST-53 complex
38954	P118	Italy	2013	cnl	11167	ST-53 complex
38956	P120	Italy	2013	cnl	11133	ST-53 complex

Supplementary Table 4. List of 144 putative hypervirulence associated genes (HVAGs).

NEIS code	BLASTp ID	Length (bp)	GC percentage	Genomic Island #	Function	Reference
NEIS0023	Putative zonular occludens toxin-like protein	1188	44.53	1 (MDA φ)	Phage	(Bille et al., 2008)
NEIS0024	Integral membrane protein	285	44.91			
NEIS0025	TspB protein	1560	47.69			
NEIS0027	Integral membrane protein	303	43.67			
NEIS0028	Integral membrane protein	231	54.63			
NEIS0029	Hypothetical protein	201	53.84			
NEIS0030	Hypothetical protein	312	53.58			
NEIS0031	phage replication initiation factor	1209	52.23			
NEIS0080	Bacteriocin transporter	237	38.4	2 (BGI-1)	Toxin	(Allunans et al., 2008)
NEIS0081	Hypothetical protein	228	33.77			
NEIS0083	Membrane protein	189	22.87			
NEIS0084	Membrane protein	138	26.81			
NEIS0085	Secretion protein	1266	37.44			
NEIS0086	Processing peptidase	2088	34.24			
NEIS0089	Hypothetical protein	147	34.69			
NEIS0169	Abi-like family protein/CAAX protease	723	35.41	3 (Abi-like)	Metabolism	
NEIS0193	Hypothetical protein	390	28.72	4	Hypothetical	
NEIS0231	SMI1 / KNR4 family protein	540	45.93	6A (SM1 I/KNR)	Cell wall synthesis	
NEIS0232	Hypothetical protein	216	30.56			
NEIS0233	Hypothetical protein	360	27.5			
NEIS0249	NmFic	576	46.01	8A (NmFic)	Cell wall synthesis	

NEIS0250	Hypothetical protein	342	39.77			
NEIS0267	Membrane protein	297	22.22	10	Hypothetical	
NEIS0295	DNA methylase	2589	38.32	11	Restriction Modification	(Claus et al., 2005)
NEIS0298	Hypothetical protein	372	33.33			
NEIS0380	PglI	1875	53.61	14 (PglI)	Membrane	(Warren et al., 2004)
NEIS2376	Maf immunity protein	336	33.64	23A (CDI)	Toxin	Proximal core gene is NEIS0222<br>Structure of locus is similar to MGI-3 (Jamet et al., 2015)
NEIS0678	Vsr	447	45.86	25A (Vsr)	Restriction Modification	
NEIS0680	type II restriction enzyme NmeDIP	1056	42.99			
NEIS0447	Hemagglutnin	375	34.13	21A/BTspA/B)	Toxin	Proximal core gene is NEIS0441 (Arenas et al., 2013)
NEIS0449	Hemagglutnin	264	31.82			
NEIS0451	Hemagglutnin	384	25.52			
NEIS0453	Hemagglutnin	489	32.23			
NEIS0454	Hemagglutnin	273	32.33			
NEIS0589	Maf immunity protein	288	26.69	23B		
NEIS0771	cytosine-specific DNA methyltransferase	1236	33.58	26	Restriction Modification	
NEIS0772	type II restriction endonuclease	1119	31.59			
NEIS0840	Phage related protein	918	52.94	29	Phage	
NEIS0855	Hypothetical protein	387	48.32			
NEIS0879	Plasmid toxin PemK	348	51.72			
NEIS0880	Transcriptional regulator	237	55.69			
NEIS0881	Death on curing proten	492	41.06		Toxin	
NEIS0887	HlyD family secretion protein	1263	36.9	30	Other	
NEIS0888	ABC transporter	2097	40.3			

NEIS0892	Hypothetical protein	117	27.35			
NEIS0893	ParB	204	35.29			
NEIS0989	Phage integrase	264	54.35	32A (P2-like)	Phage	
NEIS0992	Phage integrase	639	62.33			
NEIS0993	Phage protein	876	54.41			
NEIS0995	DNA phage binding protein	297	53.69			
NEIS0996	Phage associated protein	156	51.92			
NEIS0997	Phage repressor	378	41.27			
NEIS0998	Hypothetical protein (Phage related)	246	41.46			
NEIS0999	Hypothetical protein (Phage related)	93	44.09			
NEIS1000	Hypothetical protein (Phage related)	912	37.94			
NEIS1001	Hypothetical protein (Phage related)	483	32.51			
NEIS1002	Hypothetical protein (Phage related)	504	56.75			
NEIS1005	Hypothetical protein (Phage related)	411	50.36			
NEIS2552	Putative phage associated protein	213	57.28			
NEIS1042	Hypothetical protein	180	46.67	33A (IHT-E)	Phage	(Masignani et al., 2001)
NEIS1044	Transcriptional regulator	900	38.89			
NEIS1045	Transcriptional regulator	252	38.49			
NEIS1046	Phage transposase	1980	54.34			
NEIS1048	Phage transposase	915	50.82			
NEIS1049	Hypothetical protein (Phage related)	291	48.45			
NEIS1050	Hypothetical protein (Phage related)	126	44.44			
NEIS1051	Hypothetical protein (Phage related)	174	52.87			

NEIS1052	Hypothetical protein (Phage related)	168	52.38			
NEIS1053	Hypothetical protein (Phage related)	228	35.53			
NEIS1054 (gam)	host-nuclease inhibitor protein	519	52.22			
NEIS1057	Phage tail protein	561	61.68			
NEIS1058	Tail protein	1974	58.21			
NEIS1059	Hypothetical protein (Phage related)	606	59.24			
NEIS1060	DNA helicase	297	50.92			
NEIS1083	Hypothetical protein	354	36.72	35	Hypothetical	
NEIS1158	Type I restriction enzyme	327	41.28	38	Restriction Modification	
NEIS1193	Type III restriction-modification system endonuclease protein	2940	48.37	39 (ModB)	Restriction Modification	(Srikhanta et al., 2009)
NEIS1194	Type III restriction/modification system methyltransferase	1975	45.11			
NEIS1357	Addiction module protein	1128	50.62	41	Toxin	
NEIS1424	hypothetical protein	330	47.58	42	Hypothetical	
NEIS1803	hypothetical protein	336	38.69	52	Hypothetical	
NEIS1886	Helicase	660	39.39	54	Metabolism	
NEIS1946 (HpuA)	haemoglobin-haptoglobin utilisation protein (HpuA)	1026	52.53	56 (HpuA/B)	Iron acquistion	(Lewis et al., 1997)
NEIS1947 (HpuB)	haemoglobin-haptoglobin utilisation protein (HpuB)	2433	55.03			
NEIS2011 (LgtG)	LgtG	1059	56.37	59 (LgtG)	Membrane	(Banerjee et al., 1998), (Kahler et al., 2005)
NEIS2012 (Lpt6)	Lpt6	1653	53.96	60 (Lpt6)	Membrane	(Wright et al., 2004, Kahler et al., 2005)
NEIS2091	MafB protein	348	35.92	62 (MafI-MGI-3)	Toxin	(Jamet et al., 2015)
NEIS2092	Maf immunity protein	324	24.07			
NEIS2093	hypothetical protein	345	28.12			
NEIS1584	Death on curing proten	387	42.64	45	Toxin	

NEIS1585	Prevent host death protein	246	39.36			
NEIS0364	Hypothetical protein	549	30.6	HI 1	Phage	
NEIS0365	Hypothetical protein	564	30.32			
NEIS1861	Replication initiation factor	1209	40.45	HI 2	Phage	
NEIS1866	TspB protein	1506	48.14			
NEIS1862	Hypothetical protein	312	41.03			
NEIS1863	Hypothetical protein	201	36.32			
NEIS1864	Hypothetical protein	231	44.59			
NEIS1865	Hypothetical protein	303	42.9			
NEIS1867	Hypothetical protein	285	44.91			
NEIS1868	Hypothetical protein	1188	44.7			
NEIS2451	Hypothetical protein	222	34.68	HI 3	Phage	Proximal core gene is NEIS1719
NEIS2452	Hypothetical protein	312	41			
NEIS2453	Hypothetical protein	201	36.3			
NEIS2459	Hypothetical protein	201	36	HI 4	Phage	Proximal core gene is NEIS1469 (LbpB)
NEIS2460	Hypothetical protein	312	41.03			
NEIS2461	Hypothetical protein	222	34.68			
NEIS2836	Plasmid maintenance system killer protein	279	48.02	HI 5	Toxin	Proximal core gene is NEIS1059
NEIS2837 (ybaQ)	Transcriptional regulator	312	48.07			
NEIS0049	ctrG	957	33.33	Cps GI	capsule	(Harrison et al., 2013)
NEIS0050	cssE	1383	28.78			
NEIS0051	csc	1479	31.51			
NEIS0052	cssC	1050	40.95			
NEIS0053	cssB	687	40.17			
NEIS0054	cssA	1134	38.1			

NEIS0055	ctrA	1176	46.68			
NEIS0056	ctrB	1164	46.99			
NEIS0057	ctrC	798	42.73			
NEIS0058	ctrD	651	46.54			
NEIS0066	ctrE	2115	51.77			
NEIS0067	ctrF	1260	48.73			
NEIS0068	Hypothetical protein	1407	57.5			
NEIS0209	Hypothetical protein	501	49.3		Hypothetical	
NEIS0223	Maf immunity protein	336	27.5	MafI-MGI-2	Toxin	(Jamet et al., 2015)
NEIS0393	Hypothetical protein	402	31.09		Hypothetical	
NEIS0445	Hypothetical protein	510	25.49		Hypothetical	
NEIS0955	Hypothetical protein	288	29.86		Hypothetical	
NEIS1012	Hypothetical protein	546	36.08		Hypothetical	
NEIS1268	Hypothetical protein	288	35.76		Hypothetical	
NEIS1715	TspB protein	1620	47.04	TspB	Other	(Arenas et al., 2013)
NEIS1969	Neisseria adhesin A	1197	48.2	NadA	Other	(Capecchi et al., 2005)
NEIS2064	ATP-binding protein	1050	39.33		Other	Proximal core gene is NEIS1625
NEIS2380	Hypothetical protein	240	45.83		Hypothetical	Proximal core gene is NEIS0684
NEIS2404	Hypothetical protein	174	56.32		Hypothetical	Proximal core gene is NEIS1002
NEIS2406	Bacteriophage transposase (Mu phage)	1986	52.92		Phage	Proximal core gene is NEIS1045
NEIS2463	Antitoxin Phd_YefM, type II toxin-antitoxin system	249	39.57	Type II toxin-antitoxin	Toxin	Proximal core gene is NEIS1584
NEIS2536	Hypothetical protein	210	55.23		Hypothetical	Proximal core gene is NEIS1060
NEIS2537	Baseplate J-like protein (P2 bacteriophage)	612	59.8		Phage	Proximal core gene is NEIS1054

NEIS2551	Hypothetical protein	282	52.48		Hypothetical	Proximal core gene is NEIS0986
NEIS2583	Hypothetical protein	240	52.5		Hypothetical	Proximal core gene is NEIS1547 (GpxA)
NEIS2778	Maf B related protein	1239	45.27		Toxin	(Jamet et al., 2015)
NEIS2789	Hypothetical protein	426	49.29		Hypothetical	Proximal core gene is NEIS0269
NEIS2854	Methyltransferase D12	345			Restriction Modification	Located in the cps locus (Clemence et al., 2019)
NEIS2910	Modification methylase	1002	39.83		Restriction Modification	Located in the cps locus (Clemence et al., 2019)

REFERENCES for Supplementary TABLE 4.

ALLUNANS, J., KRISTIANSEN, K. I., ASSALKHOU, R. & BJORAS, M. 2008. Bacteriocins (meningocins) in Norwegian isolates of Neisseria meningitidis: possible role in the course of a meningococcal epidemic. *APMIS,* 116, 333-44.

ARENAS, J., SCHIPPER, K., VAN ULSEN, P., VAN DER ENDE, A. & TOMMASSEN, J. 2013. Domain exchange at the 3' end of the gene encoding the fratricide meningococcal two-partner secretion protein A. *BMC Genomics,* 14, 622.

BANERJEE, A., WANG, R., ULJON, S. N., RICE, P. A., GOTSCHLICH, E. C. & STEIN, D. C. 1998. Identification of the gene (lgtG) encoding the lipooligosaccharide beta chain synthesizing glucosyl transferase from Neisseria gonorrhoeae. *Proc Natl Acad Sci U S A,* 95, 10872-7.

BILLE, E., URE, R., GRAY, S. J., KACZMARSKI, E. B., MCCARTHY, N. D., NASSIF, X., MAIDEN, M. C. & TINSLEY, C. R. 2008. Association of a bacteriophage with meningococcal disease in young adults. *PLoS One,* 3, e3885.

CAPECCHI, B., ADU-BOBIE, J., DI MARCELLO, F., CIUCCHI, L., MASIGNANI, V., TADDEI, A., RAPPUOLI, R., PIZZA, M. & ARICO, B. 2005. Neisseria meningitidis NadA is a new invasin which promotes bacterial adhesion to and penetration into human epithelial cells. *Mol Microbiol,* 55, 687-98.

CLAUS, H., MAIDEN, M. C., WILSON, D. J., MCCARTHY, N. D., JOLLEY, K. A., URWIN, R., HESSLER, F., FROSCH, M. & VOGEL, U. 2005. Genetic analysis of meningococci carried by children and young adults. *Journal of Infectious Diseases,* 191, 1263-1271.

CLEMENCE, M. E. A., HARRISON, O. B. & MAIDEN, M. C. J. 2019. Neisseria meningitidis has acquired sequences within the capsule locus by horizontal genetic transfer. *Wellcome Open Res,* 4, 99.

HARRISON, O. B., CLAUS, H., JIANG, Y., BENNETT, J. S., BRATCHER, H. B., JOLLEY, K. A., CORTON, C., CARE, R., POOLMAN, J. T., ZOLLINGER, W. D., FRASCH, C. E., STEPHENS, D. S., FEAVERS, I., FROSCH, M., PARKHILL, J., VOGEL, U., QUAIL, M. A., BENTLEY, S. D. & MAIDEN, M. C. 2013. Description and nomenclature of Neisseria meningitidis capsule locus. *Emerg Infect Dis,* 19, 566-73.

JAMET, A., JOUSSET, A. B., EUPHRASIE, D., MUKORAKO, P., BOUCHARLAT, A., DUCOUSSO, A., CHARBIT, A. & NASSIF, X. 2015. A new family of secreted toxins in pathogenic *Neisseria* species. *PLoS Pathog,* 11, e1004592.

KAHLER, C. M., DATTA, A., TZENG, Y. L., CARLSON, R. W. & STEPHENS, D. S. 2005. Inner core assembly and structure of the lipooligosaccharide of Neisseria meningitidis: capacity of strain NMB to express all known immunotype epitopes. *Glycobiology,* 15, 409-19.

LEWIS, L. A., GRAY, E., WANG, Y. P., ROE, B. A. & DYER, D. W. 1997. Molecular characterization of hpuAB, the haemoglobin–haptoglobin-utilization operon of *Neisseria meningitidis*. *Molecular microbiology,* 23, 737-749.

MASIGNANI, V., GIULIANI, M. M., TETTELIN, H., COMANDUCCI, M., RAPPUOLI, R. & SCARLATO, V. 2001. Mu-like prophage in serogroup B Neisseria meningitidis coding for surface-exposed antigens. *Infection and immunity,* 69, 2580-2588.

SRIKHANTA, Y. N., DOWIDEIT, S. J., EDWARDS, J. L., FALSETTA, M. L., WU, H. J., HARRISON, O. B., FOX, K. L., SEIB, K. L., MAGUIRE, T. L., WANG, A. H., MAIDEN, M. C., GRIMMOND, S. M., APICELLA, M. A. & JENNINGS, M. P. 2009. Phasevarions mediate random switching of gene expression in pathogenic Neisseria. *PLoS Pathog,* 5, e1000400.

WARREN, M. J., RODDAM, L. F., POWER, P. M., TERRY, T. D. & JENNINGS, M. P. 2004. Analysis of the role of pglI in pilin glycosylation of *Neisseria meningitidis*. *FEMS Immunology & Medical Microbiology,* 41, 43-50.

WRIGHT, J. C., HOOD, D. W., RANDLE, G. A., MAKEPEACE, K., COX, A. D., LI, J., CHALMERS, R., RICHARDS, J. C. & MOXON, E. R. 2004. lpt6, a gene required for addition of phosphoethanolamine to inner-core lipopolysaccharide of Neisseria meningitidis and Haemophilus influenzae. *J Bacteriol,* 186, 6970-82.

Supplementary Table 5. Results of the Phaser analysis of the meningococcal genomes.

See methods for the definitions for simple sequence repeats (SSR), INDEL and the calculation of percentage INTACT.
Zero percent = gene is present but phased off in all instances. Grey boxes= not present in the genome.

NEIS code	BLASTp ID	Function	Core/ accessory	SSR/ INDEL	cc53	cc32	cc5	cc213	cc41/44	cc22	cc11	cc60	cc461	cc269	cc23
						Percentage of isolates INTACT									
NEIS1614 (IspA)	Membrane protein IspA	Cell division	Core	INDEL	97.96%	97.96%	38.00%	97.96%	97.96%	93.88%	97.96%	97.96%	97.96%	97.96%	97.96%
NEIS2018	Growth regulator	Cell division	Core	SSR	95.92%	95.92%	2.00%	95.92%	34.00%	95.92%	95.92%	24.00%	95.92%	95.92%	95.92%
NEIS0949	Transposase	Insertion element	Core	SSR	97.96%	44.00%	97.96%	97.96%	97.96%	58.00%	97.96%	97.96%	70.00%	97.96%	97.96%
NEIS1402	Transposase	Insertion element	Core	INDEL	97.96%	97.96%	97.96%	97.96%	97.96%	22.00%	97.96%	97.96%	72.00%	97.96%	97.96%
NEIS0218	Dihyroorotate dehydrogenase	Metabolism	Core	INDEL	93.88%	93.88%	93.88%	93.88%	93.88%	93.88%	93.88%	18.00%	93.88%	93.88%	93.88%
NEIS0235	Fused MFS/spermidine synthase	Metabolism	Core	SSR	97.96%	97.96%	4.00%	97.96%	97.96%	93.88%	97.96%	97.96%	97.96%	97.96%	97.96%
NEIS0292 (terB)	Tellurite resistance protein - TerB	Metabolism	Core	SSR	97.96%	97.96%	0.00%	2.00%	4.00%	0.00%	0.00%	97.96%	0.00%	97.96%	97.96%
NEIS0429 (basS)	Two component sensor histidine kinase (BasS)	Metabolism	Core	SSR	97.96%	97.96%	97.96%	0.00%	97.96%	0.00%	97.96%	97.96%	97.96%	97.96%	97.96%
NEIS0553 (potG)	potG - polyamine ABC transporter	Metabolism	Core	INDEL	93.88%	93.88%	93.88%	93.88%	93.88%	93.88%	4.76%	93.88%	93.88%	93.88%	2.00%
NEIS0665	Sodium bile acid symporter family	Metabolism	Core	INDEL	95.92%	95.92%	95.92%	95.92%	95.92%	95.92%	95.92%	44.00%	95.92%	95.92%	95.92%
NEIS0908	Xanthine permease	Metabolism	Core	INDEL	89.80%	89.80%	89.80%	89.80%	89.80%	89.80%	89.80%	89.80%	89.80%	89.80%	4.00%
NEIS0916	d-alanine-poly(phosphoribitol) ligase	Metabolism	Core	INDEL	93.88%	93.88%	60.00%	93.88%	93.88%	93.88%	93.88%	93.88%	93.88%	93.88%	93.88%
NEIS1062	ABC trrnasmembrane region 2	Metabolism	Core	INDEL	0.00%	0.00%	86.00%	0.00%	72.00%	0.00%	39.68%	0.00%	92.00%	92.00%	50.00%
NEIS1074	Amino acid permease	Metabolism	Core	INDEL	4.08%	4.08%	98.00%	98.00%	92.00%	98.00%	96.83%	100.00%	100.00%	100.00%	98.00%
NEIS1588 (PqiA)	PqiA - paraquat inducible protein A	Metabolism	Core	INDEL	95.92%	54.00%	95.92%	95.92%	95.92%	8.00%	1.59%	18.00%	8.00%	95.92%	2.00%
NEIS1630	L-lactate permease	Metabolism	Core	INDEL	95.92%	95.92%	95.92%	95.92%	95.92%	95.92%	95.92%	95.92%	95.92%	32.00%	95.92%
NEIS1689	polyamine ABC transporter substrate-binding protein	Metabolism	Core	SSR	95.92%	95.92%	95.92%	95.92%	95.92%	95.92%	95.92%	28.00%	22.00%	95.92%	95.92%
NEIS1724	hemerythrin domain-containing protein	Metabolism	Core	SSR	95.92%	95.92%	95.92%	95.92%	95.92%	95.92%	95.92%	95.92%	95.92%	80.00%	95.92%
NEIS1965	FecCD transporter permease	Metabolism	Core	INDEL	97.96%	97.96%	97.96%	97.96%	97.96%	97.96%	97.96%	97.96%	97.96%	97.96%	20.00%

NEIS code	BLASTp ID	Function	Core/ accessory	SSR/ INDEL	cc53	cc32	cc5	cc213	cc41/44	cc22	cc11	cc60	cc461	cc269	cc23
NEIS2026	PTS sugar transporter subunit IIA	Metabolism	Core	INDEL	93.88%	93.88%	93.88%	93.88%	16.00%	12.00%	93.88%	93.88%	93.88%	60.00%	93.88%
NEIS2056 (hemK)	hemK	Metabolism	Core	SSR	0.00%	94.00%	0.00%	0.00%	0.00%	98.00%	95.24%	96.00%	88.00%	84.00%	100.00%
NEIS0213 (pglA)	glycosyltransferase	Outer Membrane protein	Core	SSR	28.00%	28.00%	12.24%	12.00%	28.00%	26.00%	11.67%	26.00%	28.00%	44.00%	28.00%
NEIS0349 (fHbP)	factor H binding protein lipoprotein - FhbP antigen	Outer Membrane protein	Core	INDEL	57.14%	78.00%	54.00%	94.00%	70.00%	98.00%	95.24%	88.00%	57.14%	92.00%	42.00%
NEIS1156	glycosyltransferase	Outer Membrane protein	Core	SSR	26.53%	0.00%	38.78%	26.53%	32.00%	38.00%	15.00%	34.00%	24.00%	38.00%	22.00%
NEIS1364 (porA)	Porin	Outer Membrane protein	Core	INDEL	10.20%	10.20%	10.20%	98.00%	90.00%	28.00%	92.06%	100.00%	100.00%	48.00%	100.00%
NEIS1986 (lpt3)	lipooligosaccharide phosphoethanolamine transferase	Outer Membrane protein	Core	INDEL	87.76%	87.76%	87.76%	87.76%	87.76%	87.76%	55.56%	87.76%	87.76%	87.76%	44.00%
NEIS2112	Outer membrane protein OMP85 family	Outer Membrane protein	Core	INDEL	95.92%	95.92%	95.92%	95.92%	95.92%	95.92%	95.92%	2.00%	6.00%	95.92%	95.92%
NEISp2020 (porB)	Class III outer membrane porin	Outer Membrane protein	Core	INDEL	0.00%	0.00%	0.00%	0.00%	0.00%	82.00%	90.48%	0.00%	0.00%	0.00%	0.00%
NEIS2408	Phage protein - UDP-N-acetylmuramoylalanyl-D-glutamyl-2, 6-diaminopimelate--D-alanyl-D-alanine ligase domain protein	Phage	Core	INDEL	97.96%	97.96%	97.96%	97.96%	97.96%	2.00%	97.96%	97.96%	97.96%	97.96%	97.96%
NEIS0202	DNA methyltransferase	Restriction modification	Core	SSR	2.04%	0.00%	86.00%	0.00%	88.00%	94.00%	93.65%	0.00%	0.00%	0.00%	100.00%
NEIS1201	Methyltransferse domain containing protein	Restriction modification	Core	SSR	2.04%	98.00%	98.00%	96.00%	96.00%	98.00%	95.24%	100.00%	100.00%	98.00%	98.00%
NEIS1302	Class I SAM-dependent methyltransferase	Restriction modification	Core	INDEL	93.88%	93.88%	93.88%	93.88%	93.88%	93.88%	93.88%	93.88%	93.88%	48.00%	93.88%
NEIS0233	Predicted immunity protein	Toxin	Core	SSR	93.88%	93.88%	93.88%	93.88%	93.88%	93.88%	58.73%	93.88%	93.88%	93.88%	93.88%
NEIS0309	Maf immunity protein	Toxin	Core	INDEL	0.00%	94.00%	96.00%	96.00%	96.00%	98.00%	96.83%	100.00%	98.00%	98.00%	24.00%
NEIS0332 (relE/parE)	Type II toxin antitoxin system RelE/ParE	Toxin	Core	SSR	0.00%	100.00%	98.00%	98.00%	96.00%	0.00%	96.83%	98.00%	100.00%	100.00%	0.00%
NEIS1799 (mafI)	Maf immunity protein	Toxin	Core	INDEL	0.00%	94.00%	96.00%	96.00%	96.00%	96.00%	96.83%	100.00%	98.00%	100.00%	28.00%
NEIS0124	DNA-directed RNA polymerase subunit beta'	Transcription	Core	INDEL	95.92%	95.92%	95.92%	95.92%	95.92%	95.92%	95.92%	95.92%	16.00%	95.92%	95.92%
NEIS0212	RNA polymerase sigma factor	Transcription	Core	SSR	95.92%	95.92%	95.92%	2.00%	95.92%	95.92%	95.92%	95.92%	95.92%	95.92%	95.92%

NEIS code	BLASTp ID	Function	Core/ accessory	SSR/ INDEL	cc53	cc32	cc5	cc213	cc41/44	cc22	cc11	cc60	cc461	cc269	cc23
NEIS0390	RNA-binding S4 domain-containing protein	Transcription	Core	INDEL	12.24%	12.24%	12.24%	92.00%	74.00%	12.24%	12.24%	82.00%	96.00%	12.24%	98.00%
NEIS0038	RNA binding protein	Uncharacterised protein	Core	INDEL	97.96%	97.96%	97.96%	97.96%	22.00%	97.96%	97.96%	97.96%	97.96%	52.00%	97.96%
NEIS0095	Hypothetical protein	Uncharacterised protein	Core	SSR	97.96%	97.96%	0.00%	2.00%	12.00%	97.96%	9.52%	14.00%	97.96%	97.96%	97.96%
NEIS0180	Putative inner membrane protein	Uncharacterised protein	Core	SSR	95.92%	95.92%	95.92%	95.92%	95.92%	95.92%	95.92%	95.92%	95.92%	6.00%	95.92%
NEIS0308	Hypothetical protein	Uncharacterised protein	Core	SSR	91.84%	91.84%	14.00%	4.00%	91.84%	91.84%	91.84%	91.84%	91.84%	4.00%	80.00%
NEIS0579	DUF4375- domain containing protein	Uncharacterised protein	Core	INDEL	95.92%	95.92%	95.92%	95.92%	4.00%	0.00%	0.00%	2.00%	2.00%	8.00%	0.00%
NEIS0595	Hypothetical protein	Uncharacterised protein	Core	SSR	93.88%	93.88%	93.88%	93.88%	93.88%	93.88%	93.88%	70.00%	93.88%	93.88%	93.88%
NEIS0630	cryptic protein cnp1	Uncharacterised protein	Core	SSR	89.80%	89.80%	89.80%	89.80%	89.80%	89.80%	89.80%	89.80%	89.80%	80.00%	89.80%
NEIS0779	Hypothetical protein	Uncharacterised protein	Core	SSR	6.12%	6.12%	98.00%	98.00%	92.00%	98.00%	6.12%	44.00%	90.00%	98.00%	88.00%
NEIS1072	DUF2134 domain containing protein	Uncharacterised protein	Core	INDEL	97.96%	97.96%	0.00%	97.96%	97.96%	93.88%	97.96%	97.96%	97.96%	97.96%	97.96%
NEIS1147	ATP binding protein	Uncharacterised protein	Core	INDEL	95.92%	95.92%	95.92%	95.92%	95.92%	95.92%	95.92%	20.00%	95.92%	95.92%	95.92%
NEIS1660	Hypothetical protein	Uncharacterised protein	Core	SSR	0.00%	0.00%	0.00%	0.00%	92.00%	0.00%	0.00%	0.00%	0.00%	0.00%	0.00%
NEIS1804	Hypothetical protein	Uncharacterised protein	Core	INDEL	95.92%	95.92%	95.92%	18.00%	95.92%	95.92%	95.92%	95.92%	95.92%	95.92%	95.92%
NEIS1819	Hypothetical protein	Uncharacterised protein	Core	SSR	97.96%	97.96%	6.00%	97.96%	97.96%	93.88%	97.96%	97.96%	97.96%	97.96%	97.96%
NEIS3059	Hypothetical protein	Uncharacterised protein	Core	INDEL	97.96%	97.96%	0.00%	97.96%	97.96%	97.96%	97.96%	97.96%	97.96%	97.96%	97.96%
NEIS3160	Hypothetical protein	Uncharacterised protein	Core	SSR	95.92%	95.92%	0.00%	95.92%	4.00%	95.92%	95.92%	95.92%	95.92%	95.92%	95.92%
NEIS1088	Capsule synthesis protein cap A	Capsule	Accessory	SSR	97.96%	97.96%	97.96%	0.00%		97.96%	97.96%	0.00%	0.00%	32.00%	0.00%
NEIS0209	Transposase	Insertion element	Accessory	INDEL	0.00%	0.00%	96.00%	0.00%	0.00%	0.00%	96.83%	0.00%	0.00%	0.00%	0.00%
NEIS1536	Transposase	Insertion element	Accessory	SSR	0.00%			98.00%	86.00%			26.00%			
NEIS0493	NnrS family protein	Metabolism	Accessory	SSR	2.04%	60.00%	96.00%	96.00%	0.00%	96.00%	90.48%	92.00%		36.00%	100.00%
NEIS1507	MdlB domoan. ABC transmembrane transporter responsible for multidrug transport	Metabolism	Accessory	INDEL	2.04%	2.04%	98.00%	96.00%	78.00%	82.00%	95.24%	96.00%		100.00%	
NEIS1623	apbE family protein (involved in thiamine synthesis)	Metabolism	Accessory	SSR	0.00%	0.00%	96.00%	92.00%	90.00%	96.00%	0.00%	0.00%		98.00%	0.00%
NEIS0962	Chloride channel protein	Metabolism	Accessory	INDEL	97.96%	97.96%	0.00%	97.96%		97.96%	97.96%	97.96%			97.96%
NEIS1265	Sel1 repeat protein (Cytochrome C oxidase)	Metabolism	Accessory	SSR	93.88%	93.88%	93.88%		93.88%		0.00%	93.88%	93.88%	93.88%	93.88%

NEIS code	BLASTp ID	Function	Core/ accessory	SSR/ INDEL	cc53	cc32	cc5	cc213	cc41/44	cc22	cc11	cc60	cc461	cc269	cc23
NEIS1300	Hemerythrin domain-containing protein	Metabolism	Accessory	SSR	95.92%	95.92%	95.92%	95.92%	95.92%	95.92%	95.92%			60.00%	95.92%
NEIS1526	Sensor histidine kinase	Metabolism	Accessory	INDEL	97.96%	97.96%	97.96%	97.96%	16.00%	97.96%	97.96%		97.96%	97.96%	97.96%
NEIS1887	TonB dependant receptor	Metabolism	Accessory	SSR	89.80%	89.80%	2.00%			89.80%	89.80%	56.00%	89.80%		62.00%
NEIS1929	Acyltransferase	Metabolism	Accessory	INDEL	0.00%	0.00%	98.00%	0.00%	0.00%		0.00%	0.00%	0.00%	0.00%	0.00%
NEIS2107	long-chain-fatty-acid--CoA ligase	Metabolism	Accessory	SSR	97.96%	97.96%			6.00%	97.96%	97.96%			97.96%	
NEIS2779 (aniA)	Nitrite reductase	Metabolism	Accessory	SSR	93.88%	93.88%	93.88%	93.88%	93.88%		31.75%		10.00%	18.00%	6.00%
NEIS2846 (pglN2)	Glycosyltransferase	Outer Membrane protein	Accessory	INDEL	2.04%	2.04%		2.04%	56.00%	82.00%		88.00%	2.04%		98.00%
NEIS1668 (tspB)	TspB	Outer Membrane protein	Accessory	SSR	95.92%		95.92%	95.92%				95.92%	26.00%		
NEIS1859 (autA)	autotransporter outer membrane beta-barrel domain-containing protein	Outer Membrane protein	Accessory	SSR	91.84%	91.84%	91.84%	91.84%	6.00%	91.84%	91.84%		91.84%	91.84%	91.84%
NEIS2573	Repetetive large surface protein	Outer Membrane protein	Accessory	SSR	0.00%		96.00%						100.00%		
pilS	pilin	Outer Membrane protein	Accessory	INDEL	79.59%	36.00%					0.00%				
NEIS1180	DNA cytosine methyltransferase	Restriction modification	Accessory	SSR	0.00%				90.00%			30.00%			82.00%
NEIS2391	Type I restriction endonuclease subunit R	Restriction modification	Accessory	SSR	97.96%	97.96%	0.00%		97.96%	97.96%		97.96%	97.96%	97.96%	97.96%
NEIS2566 (cas9)	cas9 endonuclease	Restriction modification	Accessory	SSR	95.92%			2.00%							95.92%
NEIS0456	Hemagglutinin/hemolysin like protein	Toxin	Accessory	INDEL	95.92%	95.92%	95.92%	95.92%		95.92%	95.92%	14.00%	95.92%		
NEIS1404	DUF560 domain-containing protein	Uncharacterised protein	Accessory	INDEL	97.96%	97.96%	97.96%		16.00%	97.96%	97.96%	97.96%	10.00%	52.00%	97.96%
NEIS2531	Hypothetical protein	Uncharacterised protein	Accessory	SSR	97.96%	97.96%	0.00%		97.96%	97.96%	0.00%	97.96%	0.00%	0.00%	0.00%
NEIS0112	ATP binding protrin	Uncharacterised protein	Accessory	INDEL	0.00%	0.00%	94.00%	98.00%	0.00%	98.00%	0.00%	0.00%		96.00%	98.00%
NEIS0368	Hypothetical protein	Uncharacterised protein	Accessory	SSR	2.04%		2.04%	42.00%	2.04%	2.04%	2.04%	86.00%	88.00%	2.04%	88.00%
NEIS0458	Hypothetical protein	Uncharacterised protein	Accessory	SSR	91.84%	91.84%	91.84%	4.00%	12.00%	91.84%	91.84%	91.84%	91.84%		91.84%
NEIS0594	Hypothetical protein	Uncharacterised protein	Accessory	SSR	91.84%		91.84%	91.84%				68.00%	91.84%		
NEIS0604	Supressor of fused domain protein (SUFU)	Uncharacterised protein	Accessory	SSR	85.71%			85.71%	85.71%	85.71%	85.71%		85.71%	56.00%	

NEIS code	BLASTp ID	Function	Core/ accessory	SSR/ INDEL	cc53	cc32	cc5	cc213	cc41/44	cc22	cc11	cc60	cc461	cc269	cc23
NEIS0607	Hypothetical protein	Uncharacterised protein	Accessory	SSR	93.88%	93.88%	93.88%	93.88%	93.88%	93.88%	93.88%	93.88%	0.00%	93.88%	
NEIS0838	Hypothetical protein	Uncharacterised protein	Accessory	INDEL	0.00%	0.00%			96.00%		96.83%			92.00%	
NEIS1014	Hypothetical protein	Uncharacterised protein	Accessory	INDEL	97.96%		97.96%	97.96%	97.96%	10.00%	97.96%	97.96%	97.96%		97.96%
NEIS1161	Autotransporter	Uncharacterised protein	Accessory	SSR	93.88%	78.00%	93.88%	2.00%	68.00%	93.88%	58.73%	93.88%	93.88%		93.88%
NEIS1334	Alcohol dehydrogenase	Uncharacterised protein	Accessory	SSR	2.04%	58.00%	2.04%		2.04%	98.00%	2.04%		98.00%		
NEIS1458 (YbhB/YbcL)	YbhB/YbcL family Raf kinase inhibitor-like protein	Uncharacterised protein	Accessory	INDEL	95.92%	95.92%	95.92%	0.00%	95.92%		95.92%		95.92%	48.00%	95.92%
NEIS1793	Supressor of fused domain protein (SUFU)	Uncharacterised protein	Accessory	SSR	0.00%	0.00%	72.00%	98.00%	90.00%	98.00%	96.83%	82.00%	42.00%	52.00%	38.00%
NEIS1801	Hypothetical protein	Uncharacterised protein	Accessory	SSR	10.20%		10.20%		90.00%		95.24%			98.00%	96.00%
NEIS1875	Hypothetical protein	Uncharacterised protein	Accessory	SSR	93.88%	0.00%			93.88%	93.88%	93.88%	93.88%		54.00%	
NEIS2097	Hypothetical protein	Uncharacterised protein	Accessory	SSR	95.92%			95.92%		95.92%	95.92%	95.92%	2.00%	95.92%	95.92%
NEIS2367	Hypothetical protein	Uncharacterised protein	Accessory	SSR	95.92%							95.92%	2.00%		95.92%
NEIS2381	DUF333 domain containing protein	Uncharacterised protein	Accessory	SSR	97.96%	97.96%	97.96%	97.96%	97.96%	26.00%	97.96%	97.96%	97.96%		0.00%
NEIS2383	Hypothetical protein	Uncharacterised protein	Accessory	SSR	0.00%	95.00%	0.00%		90.00%					98.00%	
NEIS2387	Hypothetical protein	Uncharacterised protein	Accessory	INDEL	0.00%	94.00%	84.00%		0.00%	98.00%		92.00%		100.00%	100.00%
NEIS2405	Hypothetical protein	Uncharacterised protein	Accessory	INDEL	0.00%	100.00%	0.00%	0.00%	0.00%	0.00%	0.00%	0.00%	0.00%		0.00%
NEIS2520	Hypothetical protein	Uncharacterised protein	Accessory	INDEL	89.80%									44.00%	18.00%
NEIS2743 (cnl)		Uncharacterised protein	Accessory	SSR	93.88%						65.08%			64.00%	0.00%
NEIS3025	Hypothetical protein	Uncharacterised protein	Accessory	INDEL	0.00%				84.00%						
NEIS3053	Hypothetical protein	Uncharacterised protein	Accessory	INDEL	93.88%		0.00%		56.00%		0.00%				
NEIS3060	Hypothetical protein	Uncharacterised protein	Accessory	SSR	0.00%	100.00%		0.00%	0.00%		0.00%	0.00%	0.00%	98.00%	
NEIS3091	Hypothetical protein	Uncharacterised protein	Accessory	SSR	95.92%	95.92%	95.92%	95.92%	95.92%		95.92%	95.92%	38.00%	48.00%	95.92%
NEIS3112	Hypothetical protein	Uncharacterised protein	Accessory	INDEL	0.00%				80.00%		41.27%	84.00%	96.00%		
NEIS3119	Hypothetical protein	Uncharacterised protein	Accessory	SSR	95.92%				10.00%			95.92%			
